# MLL-AF4 Spreading Identifies Binding Sites that Are Distinct from Super-Enhancers and that Govern Sensitivity to DOT1L Inhibition in Leukemia

**DOI:** 10.1016/j.celrep.2016.12.054

**Published:** 2017-01-10

**Authors:** Jon Kerry, Laura Godfrey, Emmanouela Repapi, Marta Tapia, Neil P. Blackledge, Helen Ma, Erica Ballabio, Sorcha O’Byrne, Frida Ponthan, Olaf Heidenreich, Anindita Roy, Irene Roberts, Marina Konopleva, Robert J. Klose, Huimin Geng, Thomas A. Milne

**Affiliations:** 1 MRC, Molecular Haematology Unit, NIHR Oxford Biomedical Research Centre Programme, University of Oxford, John Radcliffe Hospital, Oxford OX3 9DS, UK; 2 Computational Biology Research Group, Weatherall Institute of Molecular Medicine, University of Oxford, John Radcliffe Hospital, Oxford OX3 9DS, UK; 3 Laboratory of Chromatin Biology and Transcription, Department of Biochemistry, University of Oxford, Oxford OX1 3QU, UK; 4 Department of Leukemia, The University of Texas MD Anderson Cancer Center, Houston, TX 77030, USA; 5 Department of Paediatrics, University of Oxford, Children’s Hospital, John Radcliffe, Oxford OX3 9DU, UK; 6 Wolfson Childhood Cancer Research Centre, Northern Institute for Cancer Research, Newcastle University, Newcastle Upon Tyne NE1 7RU, UK; 7 Department of Laboratory Medicine, University of California, San Francisco, San Francisco, CA 94143, USA

**Keywords:** MLL, MLL-AF4, DOT1L, H3K79me2, leukemia, epigenetic therapy, drug combination therapy, epigenetic spreading

## Abstract

Understanding the underlying molecular mechanisms of defined cancers is crucial for effective personalized therapies. Translocations of the mixed-lineage leukemia (*MLL*) gene produce fusion proteins such as MLL-AF4 that disrupt epigenetic pathways and cause poor-prognosis leukemias. Here, we find that at a subset of gene targets, MLL-AF4 binding spreads into the gene body and is associated with the spreading of Menin binding, increased transcription, increased H3K79 methylation (H3K79me2/3), a disruption of normal H3K36me3 patterns, and unmethylated CpG regions in the gene body. Compared to other H3K79me2/3 marked genes, MLL-AF4 spreading gene expression is downregulated by inhibitors of the H3K79 methyltransferase DOT1L. This sensitivity mediates synergistic interactions with additional targeted drug treatments. Therefore, epigenetic spreading and enhanced susceptibility to epidrugs provides a potential marker for better understanding combination therapies in humans.

## Highlights

• MLL-AF4 binding requires an unmethylated CpG (uCpG) island and Menin
• MLL-AF4 and Menin can spread into the gene body of some targets
• Spreading targets are highly transcribed and have an aberrant chromatin signature
• Spreading of MLL-AF4 is a predictor of sensitivity to DOT1L inhibitors

## Graphical Abstract

**Figure:**
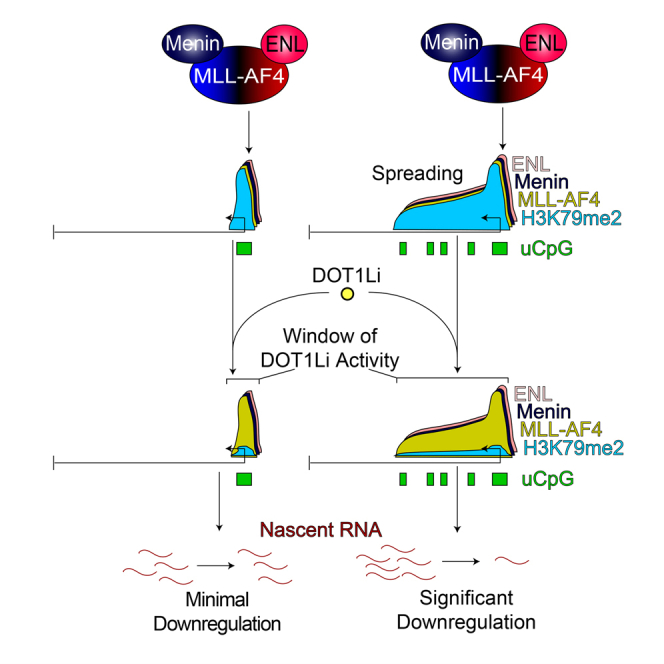

## Teaser

Translocations of the MLL gene produce fusion proteins such as MLL-AF4 that cause poor-prognosis leukemias. Kerry et al. show that MLL-AF4 can spread into the gene body of some target genes. Spreading targets have an aberrant chromatin signature and are sensitive to DOT1L inhibitors.

## Introduction

Translocations of the mixed-lineage leukemia (*MLL*) gene produce over 120 different MLL fusion proteins (MLL-FPs) that cause aggressive acute leukemias, the most common one being the *MLL-AF4* fusion (Ballabio and Milne, 2012, Meyer et al., 2013). Despite much progress in the treatment of childhood leukemias, infants carrying *MLL* rearrangements have a very poor prognosis (Pui et al., 2011); thus, improving therapies for MLL-FP patients remains an unmet need. Because MLL-FPs are considered to be the main drivers of leukemogenesis, their function regulating downstream target genes is key to understanding MLL-rearranged (MLLr) leukemias and for designing targeted therapies.

**Figure 1:**
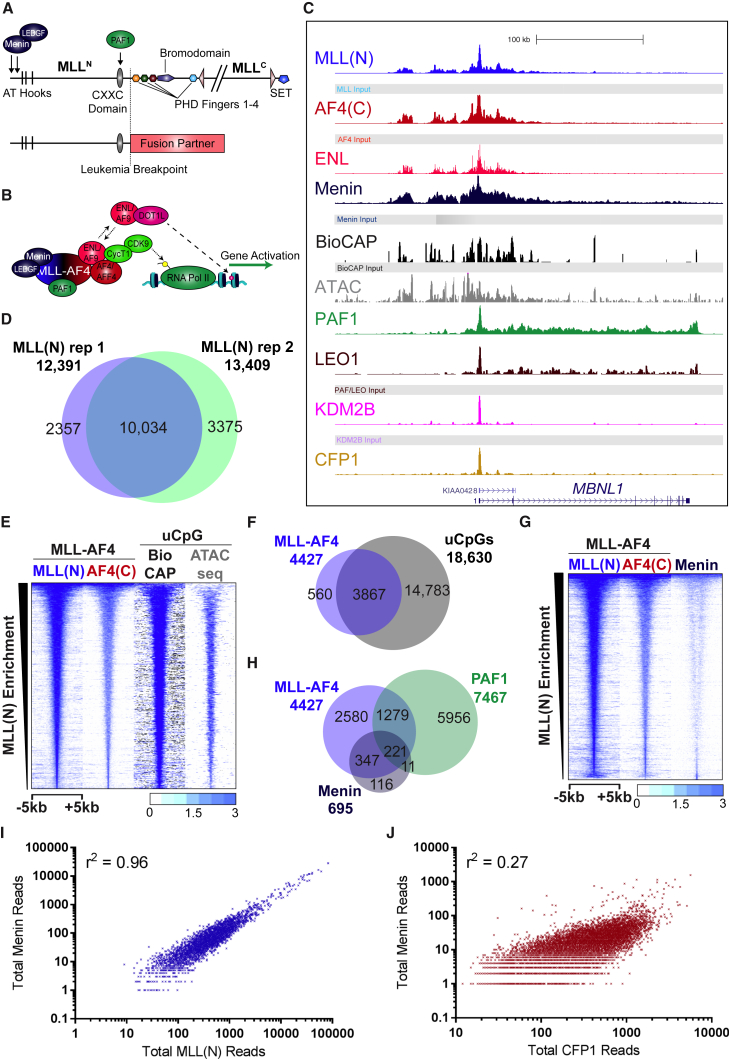
MLL-AF4 Is Recruited Exclusively to uCpG Regions Bound by Menin (A) Schematic showing MLL and MLL fusion protein interaction sites. (B) Schematic showing the MLL-AF4 core complex. (C) Example ChIP-seq, Bio-CAP-seq, and ATAC-seq tracks in SEM cells. (D) Venn diagram showing overlap between two biological replicates of MLL(N) ChIP-seq. (E) Heatmap showing ChIP-seq, Bio-CAP-seq, and ATAC-seq reads at all 4,427 MLL-AF4 binding sites in SEM cells. Scale bar represents tags per base pair (bp) per 10^7^ reads. (F) Venn diagram showing overlap between MLL-AF4 binding sites and uCpG regions (Bio-CAP-seq and ATAC-seq) in SEM cells. (G) Heatmap showing MLL(N), AF4(C), and Menin ChIP-seq reads at all MLL-AF4 binding sites in SEM cells. Scale bar as in (E). (H) Venn diagram showing overlap between MLL-AF4, PAF1, and Menin binding sites in SEM cells. (I and J) Scatterplot showing a strong correlation (r^2^ = 0.96) between MLL(N) and Menin ChIP-seq signal at all MLL-AF4 peaks (I) in SEM cells and a weak correlation between Menin and CFP1 (r^2^ = 0.27) at all CFP1 peaks (J) in SEM cells. See also Figure S1.

MLL-FPs retain several domains (Figure 1A) including a CXXC domain that binds specifically to unmethylated CpG (uCpG) DNA (Birke et al., 2002), interaction sites with the multiple endocrine neoplasia type 1 (Menin) (Yokoyama et al., 2005) and lens epithelium-derived growth factor (LEDGF) proteins (Yokoyama and Cleary, 2008), and an interaction with the polymerase-associated factor protein complex (PAFc) (Milne et al., 2010, Muntean et al., 2010). Recruitment of MLL-FPs to gene targets is thought to be controlled by Menin, LEDGF, and PAFc interactions as well as CXXC binding to uCpGs (Milne et al., 2010, Muntean et al., 2010, Okuda et al., 2014, Yokoyama and Cleary, 2008, Yokoyama et al., 2005). Supporting this, a minimal MLL-FP containing just the PWWP domain of LEDGF, the CXXC domain of MLL, and the transactivation domain of the fusion partner can transform bone marrow progenitors and recapitulate MLL-FP binding at a few select genes (Okuda et al., 2014). However, a minimal CXXC domain can be recruited to the *HoxA9* locus in the absence of a Menin/LEDGF interaction (Milne et al., 2010), although others have suggested that the CXXC domain has no role in recruitment and instead protects uCpG sites from methylation (Risner et al., 2013). Recent data also suggest that Menin is unimportant for wild-type MLL (Borkin et al., 2015, Li et al., 2013), whereas LEDGF is required for MLL but not MLL-FP recruitment (Zhu et al., 2016). Thus, it still remains an open question exactly how MLL-FPs are recruited to particular gene targets.

MLL-FP recruitment is associated with increased histone 3 lysine 79 di- and tri-methylation (H3K79Me2/3) at target genes, an epigenetic mark associated with gene activation (Bernt et al., 2011, Guenther et al., 2008, Krivtsov et al., 2008, Milne et al., 2005). H3K79Me2/3 levels are controlled by the disruptor of telomeric silencing 1-like (DOT1L) protein (Jones et al., 2008). In MLL-FP leukemias, DOT1L directly interacts with AF9 or ENL (Biswas et al., 2011, Mueller et al., 2007), and can be mis-targeted to MLL-FP-bound genes where it is associated with inappropriate activation of gene expression (Milne et al., 2005) (Figure 1B). A recent study analyzing MLL-ENL binding suggests that there are two distinct classes of binding: proximal (5′) or distal (3′) to the transcription start site, with proximal binding being particularly sensitive to DOT1L inhibition (Garcia-Cuellar et al., 2016). MLL-AF4 can also bind in broad regions of up to 100 kb that correlate with large domains of H3K4me3 (Guenther et al., 2008) and MLL-AF9 transformed mouse bone marrow cells display H3K79me2 peaks with a similar wide spatial distribution (Bernt et al., 2011). Despite all this work, there is no current consensus on whether the main activity of MLL-FPs is the recruitment of DOT1L or whether different binding patterns of MLL-FPs are associated with distinct functional outcomes.

Here, we reveal a strong co-dependent relationship between MLL-AF4 and Menin binding at a small number of target genes containing uCpGs. At a subset of these gene targets, we observe MLL-AF4 and Menin spreading that is bookended by uCpGs. These spreading targets are distinct from super-enhancers, are associated with high levels of gene transcription, have an aberrant H3K79me2/H3K36me3 signature, and are predictive of a poor overall survival in patients with acute lymphoblastic leukemia (ALL). These gene targets also display a remarkable dependence on H3K79me2 and the fusion protein for their sustained expression in leukemia. Together, this work shows that MLL-FP spreading occurs at genes important in MLL leukemogenesis and has the potential to act as a biomarker for therapeutic response.

## Results

### MLL-AF4 Binds Exclusively to a Subset of uCpGs

Using MLL(N) and AF4(C) chromatin immunoprecipitation sequencing (ChIP-seq) in the human MLL-AF4 SEM cell line (Figure 1C), we identified 4,427 peaks and a gene set of 2,597 unique genes (Table S1). MLL(N) ChIP-seq replicates had 81% peaks in common (Figure 1D), which identified 96.4% promoter-bound MLL(N) gene targets from our original ChIP-seq dataset (Table S1, “Overlaps”). This gave us high confidence in the reproducibility of our gene target identification. To test the specificity of the MLL-AF4 target set, we performed MLL-AF4 small interfering RNA (siRNA) knockdowns coupled with nascent RNA sequencing (RNA-seq) (Figure S1A) and MLL-AF4 ChIP-seq (Figure S1B). Our MLL-AF4 gene target set was significantly downregulated at most genes (Figure S1A, p < 0.01, Mann-Whitney U test) and lost MLL-AF4 ChIP-seq signal at 85% of target gene promoters (Figures S1B and S1C). Among the 15% of MLL-AF4 gene targets with no reduced ChIP-seq signal, one-third showed a significant change in gene expression following MLL-AF4 knockdown (Figure S1C). This suggests that these targets are also directly regulated by MLL-AF4, even though they consist primarily of promoters with a low MLL(N) ChIP-seq signal (Figure S1D).

If the CXXC domain is essential for MLL-AF4 recruitment, we would expect all MLL-AF4 binding sites to occur at regions of uCpGs. To test this, we used a biotinylated CXXC affinity purification (Bio-CAP) assay (Blackledge et al., 2012) for high-sensitivity detection of regions of uCpG dinucleotides in SEM cells, combined with an assay for transposase-accessible chromatin sequencing (ATAC-seq) (Buenrostro et al., 2013) to identify regions of open chromatin (Figure 1C). Similar to results using non-methylated CpG/methylated-CpG island recovery assay sequencing (CIRA/MIRA-seq) with MLL-AF6 (Okuda et al., 2014), all MLL-AF4 binding occurred at open uCpG regions (Figure 1E) with the highest uCpG enrichment occurring at the center of MLL-AF4 binding sites (Figure S1E). However, MLL-AF4 binding occurred at only 20% of uCpG sites (Figure 1F), indicating that open uCpG sites alone are not sufficient for MLL-AF4 recruitment. The Venn diagram (Figure 1F) shows a few MLL-AF4 sites that do not overlap with uCpGs, but it is clear from the heatmap that all MLL-AF4 binding sites occur at uCpG sites. The discrepancy is likely due to the reduced sensitivity of peak-calling programs used for the Venn diagram analysis.

To determine whether other CXXC domain-containing proteins (“CXXC proteins” from now on) are also restricted to only a proportion of uCpG sites, we performed ChIP-seq for CFP1 (a CXXC protein member of the SET1 complex associated with gene activation), and KDM2B (a CXXC protein involved in the recruitment of the polycomb group repressive complex [PRC] [Farcas et al., 2012]), in SEM cells (Figure 1C). In contrast to MLL-AF4, CFP1 and KDM2B bound more ubiquitously to uCpG sites, being found at 50% and 89% of all uCpG sites, respectively (Figure S1F). Because the CXXC domains of MLL-AF4, CFP1, and KDM2B are highly related (Long et al., 2013), the differences in the number of bound uCpGs may be due to other protein interactions influencing recruitment.

### Genome-wide Recruitment of Menin Mirrors that of MLL-AF4

To investigate whether MLL-AF4-specific interactions contribute to uCpG binding, we analyzed two complexes thought to be involved in MLL-FP recruitment: Menin/LEDGF and PAFc (Figures 1A–1C). Except for a very few Menin binding sites (Figure 1G, very bottom of heatmap), we found that almost all MLL-AF4 binding sites overlap with detectable Menin binding (Figure 1G). Many MLL-AF4 binding sites had only low levels of detectable Menin (Figure 1G), and thus strict peak-calling parameters produce an MLL-AF4/Menin overlap at only a subset of binding sites (Figure 1H). However, when MLL-AF4 binding sites were separated into either high or low Menin binding, we saw a direct relationship between levels of Menin binding and levels of MLL-AF4 binding (Figure S1G). Furthermore, a direct comparison of MLL(N) and Menin ChIP-seq reads at MLL-AF4 binding sites showed a significantly strong positive correlation (r^2^ = 0.96) (Figure 1I), whereas neither KDM2B nor CFP1 binding correlated with Menin (Figures 1J and S1H). Thus, an association with Menin represents a feature that may serve to restrict MLL-AF4 recruitment to a particular subset of uCpG sites.

ChIP-seq on two members of PAFc, PAF1 and LEO1 (Figure 1C), overlapped with less than one-half of MLL-AF4 binding sites, and 4,892 (78%) of PAFc binding sites had no MLL-AF4 binding (Figure S1I). Thus, compared to Menin binding, there is very little evidence for an MLL-AF4:PAF1 association genome-wide (Figure 1H), but it is possible that PAFc is necessary for recruitment only at select sites.

### The Menin:MLL-AF4 Interaction Is Sufficient for Recruitment

**Figure 2:**
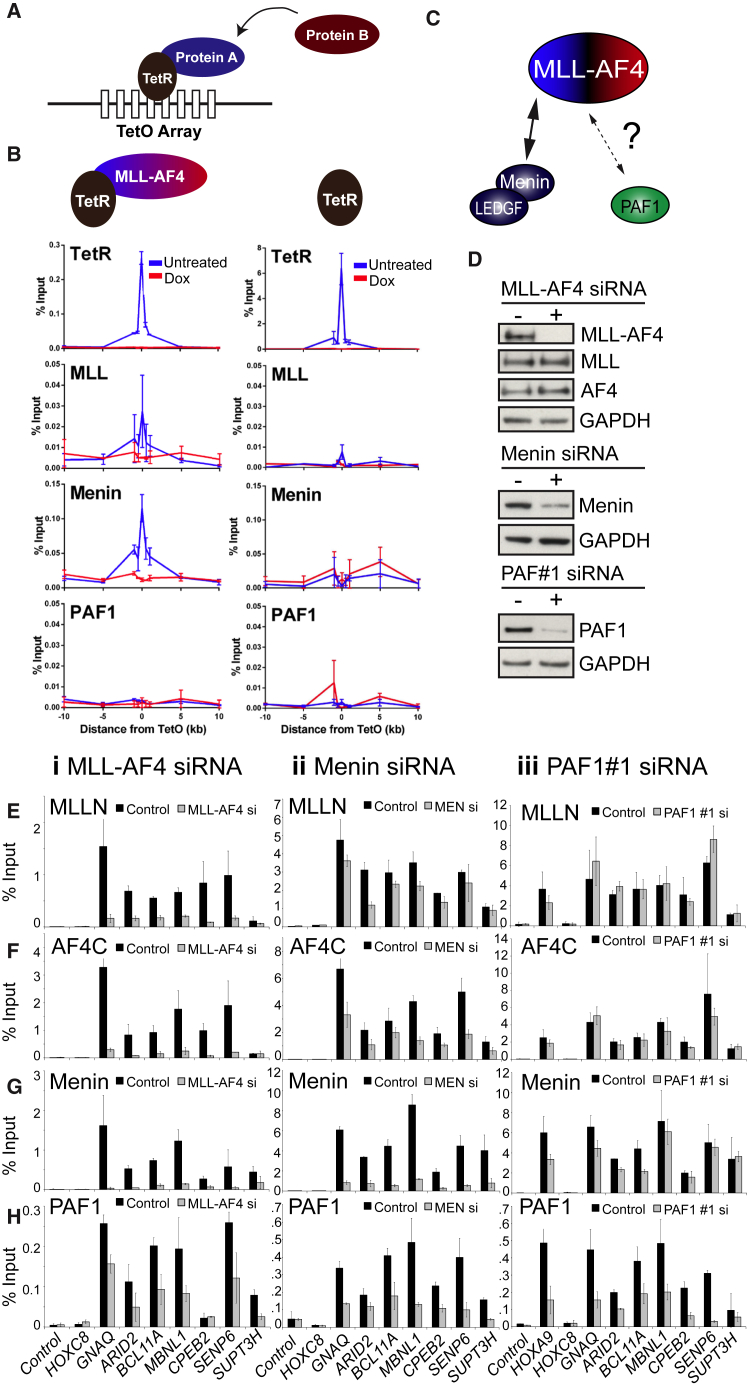
The MLL-AF4:Menin Interaction Is Sufficient but Not Necessary for Recruitment (A) The Tet-repressor (TetR) recruitment system. An array of Tet-operator (TetO) sequences was centrally inserted into a BAC lacking known promoter, enhancer, or uCpG features, and the BAC was inserted into chromosome 8 of mouse embryonic stem cells (mESCs) (Blackledge et al., 2014). Proteins of interest fused to the TetR can be anchored at the TetO array. The TetR-TetO interaction can be disrupted with doxycycline treatment, allowing one to test whether recruitment of a specific protein is dependent on the continuous presence of a particular TetR fusion. (B) ChIP-qPCR showing the binding of TetR-MLL-AF4 (using FS2 [TetR] and MLL(N) antibody), Menin, and PAF1 in TetO mESCs transfected with TetR-MLL-AF4 (left panel) and in TetR-only control mESCs (right panel). Error bars represent the SD of two biological replicates. Red line, with doxycycline. (C) The TetR experiments indicate that there is a strong interaction between MLL-AF4 and Menin and an undetectable interaction between MLL-AF4 and PAF1. (D) SEM cells were treated with MLL-AF4, Menin, or PAF1 siRNAs, and individual representative western blots from the experiments in E–H are shown. (E–H) MLL-N (E), AF4-C (F), Menin (G), and PAF1 (H) ChIP in control (black bars) and siRNA-treated (gray bars) SEM cells as follows: column i, MLL-AF4 siRNA; column ii, Menin siRNA; and column iii, PAF1 siRNA. Note that the control samples are the same between PAF1#1 siRNA and PAF1#2 siRNA (see Figure S2H) experiments as these were performed in parallel. Error bars represent the SD of at least three biological replicates. See also Figure S2.

To directly test the functionality of the interactions between Menin, MLL-AF4, and potentially PAFc, we used a Tet-repressor (TetR) system (see Figure 2A legend) previously designed to investigate the recruitment of PRC proteins (Blackledge et al., 2014). Using ChIP-qPCR, we detected binding of Menin but not PAF1 in the presence of TetR-MLL-AF4 but not the TetR-only control (Figure 2B, left versus right panel), and recruitment was lost upon treatment with doxycycline (Figure 2B, left panel, red line). Reciprocal experiments using TetR-Menin- and TetR-PAF1-expressing mESC lines transiently transfected with MLL-AF4 produced equivalent results (Figures S2A and S2B). Despite being able to recruit other members of PAFc, TetR-PAF1 was not sufficient to recruit MLL-AF4 (Figures S2B and S2C). Expression of different constructs was confirmed with either western blot or qPCR (Figures S2D and S2E). It has been recently shown that knockdowns of LEDGF sometimes lead to an increase in MLL-FP binding (Zhu et al., 2016). Similar to Zhu et al., we noticed a slight increase in MLL(N) ChIP at TetO in the presence of *Ledgf* siRNA (Figures S2F and S2G), although we were only able to achieve a 30% *Ledgf* knockdown at the RNA level (Figure S2F).

Our results so far support previous models suggesting that Menin recruits MLL-FPs (Yokoyama et al., 2005, Yokoyama et al., 2010), and contrasts with previous reports that suggest that PAFc can recruit MLL-FPs (Milne et al., 2010, Muntean et al., 2010) (Figure 2C). The TetR assay does not establish directionality of these interactions; thus, it is also possible that MLL-FPs can recruit Menin (Caslini et al., 2007), or that the two proteins co-stabilize each other, as has recently been suggested for LEDGF and wild-type MLL (Zhu et al., 2016). In addition, it is possible that a relatively weak MLL-AF4:PAFc interaction is stabilized by other interactions when it occurs at active genes. To explore these issues further, we performed MLL-AF4, PAF1, or Menin siRNA knockdowns in SEM cells (Figures 2D–2H). MLL-AF4 knockdowns have a strong effect on the binding of Menin to gene targets (Figure 2Gi) and a moderate but detectable effect on PAF1 binding (Figure 2Hi). Menin knockdowns reduce both MLL-AF4 and PAF1 binding to gene targets (Figures 2E–2Hii), whereas two different PAF1 siRNAs produce a similar result in that they reduce Menin binding slightly but have little effect on MLL-AF4 except at the *HOXA9* locus (Figures 2E–2Hiii and S2H). Together, these data show that there is a complex co-recruitment relationship between MLL-AF4 and Menin, and that PAF1 does not have a major role in recruiting MLL-AF4 to most gene targets. However, MLL-AF4 either directly or indirectly, has a role in maintaining stable PAF1 binding at specific gene targets. To analyze MLL-AF4 function in further detail, we next tried to determine whether MLL-AF4 displayed distinct binding profiles at different subsets of genes.

### Spreading of MLL-AF4 Marks a Subset of Highly Expressed Genes

**Figure 3:**
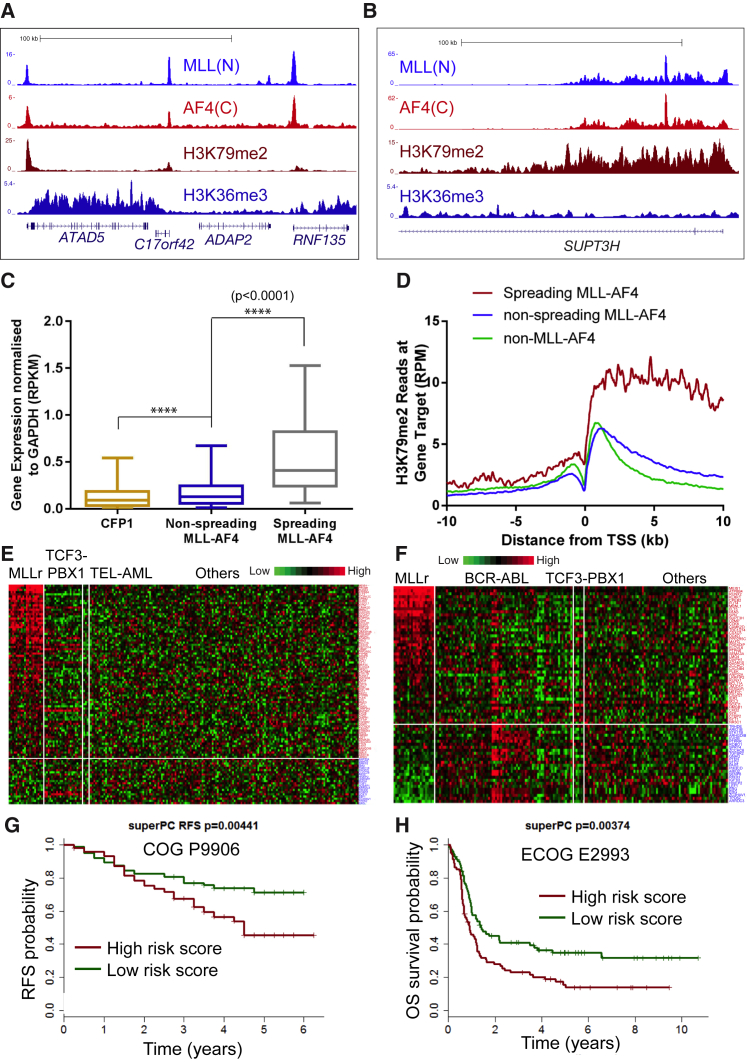
MLL-AF4 Spreading Marks a Subset of Highly Expressed Genes (A and B) Example ChIP-seq tracks showing promoter-restricted (A) or spreading (B) of MLL-AF4, H3K79me2, and H3K36me3 in SEM cells. (C) Box-and-whisker plot showing the median and interquartile (IQ) range of gene expression of spreading MLL-AF4 gene targets (n = 149) compared to non-spreading MLL-AF4 targets (n = 2,878) and CFP1 targets (n = 6,147). Gene expression, normalized to *GAPDH* expression, is derived from four biological replicates of nascent RNA-seq in SEM cells. ^∗∗∗∗^p < 0.0001, two-tailed Mann-Whitney U test. (D) Composite binding plot of H3K79me2 ChIP-seq reads at the TSS of gene targets of spreading MLL-AF4 (red), non-spreading MLL-AF4 (blue), and non-MLL-AF4 targets that are marked by H3K79me2 (green). (E and F) Heatmap expression data showing overexpression of 79% (E, COG P9906 patients [Harvey et al., 2010]) or 64% (F, ECOG 2993 patients [Geng et al., 2012]) of SEM spreading targets in MLL patients (MLLr) compared to the ALL patient subsets indicated. (G and H) Super-PC analysis (Bair and Tibshirani, 2004) using the spreading-gene target list showing relapse-free survival (RFS) of ALL patients (G, COG P9906 [Harvey et al., 2010]) and overall survival (OS) of ALL patients (H, ECOG 2993 [Geng et al., 2012]) classified by either high- or low-risk scores computed using the spreading MLL-AF4 gene targets in a super-PC model; see Supplemental Experimental Procedures, Survival Analysis, for details. See also Figure S3.

An analysis of MLL-AF4 binding profiles revealed two patterns of binding. The majority of MLL-AF4 binding sites displayed narrow binding at the promoter and a normal pattern of H3K79me2 and H3K36me3 (Figure 3A). We also occasionally observed MLL-AF4 spreading greater than 4 kb into the gene body without exceeding the end of the gene, and this was associated with H3K79me2 spreading and a reduction or loss of H3K36me3 throughout the gene body (Figure 3B). Spreading was observed at 149 (3.4%) MLL-AF4 gene target isoforms (117 unique gene targets) in SEM cells (Figure S3A; Table S2). A Gene Ontology analysis revealed that spreading occurred at genes involved in hematopoiesis as well as lymphocyte activation and differentiation, showing that it could have a role in leukemia initiation or maintenance (Figure S3B). We confirmed that spreading was specific to MLL-AF4 using *MLL-AF4* siRNA knockdowns followed by ChIP-qPCR in regions of spreading at specific targets (Figure S3C). Spreading is reminiscent of broad MLL-AF4 binding domains at sites of broad H3K4me3 (Guenther et al., 2008), although we found that there was less than a 50% overlap between our spreading dataset and the MLL-AF4 target set originally identified by Guenther et al. (Figure S3D).

To test whether MLL-AF4 spreading was a marker of significant functional activity, we analyzed nascent RNA-seq data and found that spreading MLL-AF4 targets showed significantly higher expression compared to targets of non-spreading MLL-AF4, or active gene targets bound by CFP1 (p < 0.0001, two-tailed Mann-Whitney U test; Figure 3C). Spreading targets were also highly enriched for H3K79me2 at the 5′ end of the gene compared to non-spreading MLL-AF4 or non-MLL-AF4 targets (Figure 3D). The increased enrichment of H3K79me2 along with its spread into the gene body, strongly suggests that the H3K79me2 pattern observed is a consequence of spreading MLL-AF4.

Because broad MLL-FP binding domains have been observed previously (Bernt et al., 2011, Guenther et al., 2008), one possibility is that spreading identifies bona fide MLL-AF4 target genes, whereas non-spreading peaks represent wild-type MLL and AF4 co-bound sites. To test this, we separated the MLL-AF4 siRNA nascent RNA-seq and ChIP-seq datasets (Figures S1A–S1D) into spreading and non-spreading target sets. We found that almost all spreading and non-spreading targets are bound by MLL-AF4, but spreading targets are more likely to be downregulated by a loss of MLL-AF4 (Figures S3E and S3F). A recently generated FLAG tagged MLL-Af4 ChIP-seq experiment in CD34^+^ cord blood cells (Lin et al., 2016) allowed us to unambiguously identify MLL-Af4 binding sites in a primary transformed cell. FLAG-MLL-Af4 ChIP-seq identified almost 3,000 MLL-Af4 gene targets, similar to the number we obtained in SEM cells (Lin et al., 2016). FLAG-MLL-Af4 binding could be divided into both spreading and non-spreading targets, about 40%–50% of which overlapped with MLL-AF4 targets in SEM cells (Figures S3G–S3I). Taken together, this suggests that MLL-AF4 can display both spreading and non-spreading binding patterns, but spreading gene targets are less common and are more significantly associated with a dependence on MLL-AF4 for their activation.

In order to better understand the significance of our spreading target set, we analyzed the expression profile of SEM spreading targets in two different patient cohorts and found that 64%–79% of SEM spreading targets are overexpressed in MLLr ALL patients (Figures 3E and 3F). Using a super-PC analysis (Bair and Tibshirani, 2004), we also found that there is a signature within the spreading target set that is predictive of a poor prognosis in patients (Figures 3G and 3H). Thus, MLL-AF4 spreading targets also have clinical significance in patients.

### Spreading Is Common among MLL Fusion Proteins but Not Wild-Type MLL

**Figure 4:**
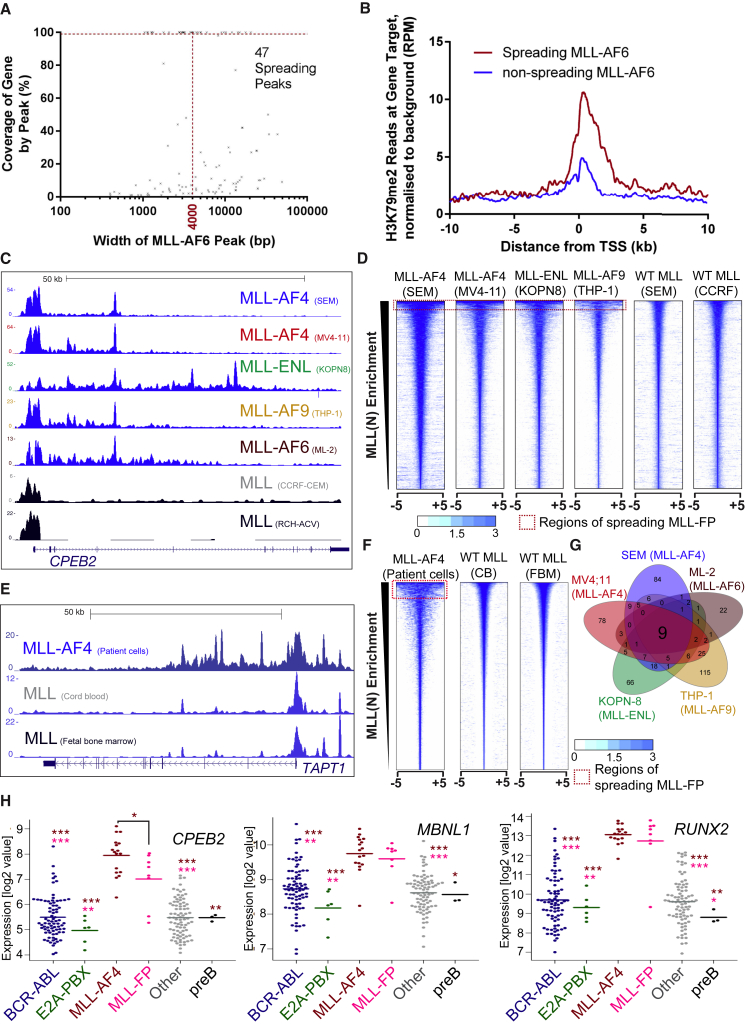
MLL-FP Spreading Occurs in Multiple in MLL Leukemias (A) Spreading MLL-AF6 peaks were defined as peaks that extend greater than 4 kb from the TSS into the gene body without going beyond the end of the gene. Using these criteria, 47 spreading MLL-AF6 peaks were identified in ML-2 cells (Table S2). (B) Composite binding plot of H3K79me2 ChIP-seq reads at the TSS of gene targets of spreading MLL-AF6 (red) and non-spreading MLL-AF6 (blue) in ML-2 cells. (C) Example ChIP-seq tracks of MLL(N) in MLL-FP and germline MLL cell lines. (D) Heatmaps of MLL(N) ChIP-seq reads from different MLL-FP cell lines as well as wild-type MLL in SEM cells and in non-MLLr cell lines. Red dotted line indicates spreading across a 10-kb window. Scale bar represents tags per base pair per 10^7^ reads. (E) Example ChIP-seq tracks of MLL(N) showing spreading in MLL-AF4 patient cells (top) compared to wild-type MLL in mononuclear cells derived from cord blood (middle) and fetal bone marrow (bottom). (F) Heatmaps showing MLL(N) ChIP-seq reads from the experiments in (E); scale and red line as in (D). (G) Venn diagram showing the overlap between gene targets of spreading MLL(N) ChIP-seq several MLLr cell lines. (H) *CPEB2*, *MBNL1*, and *RUNX2* are overexpressed in MLL-AF4 and other MLL-FP patients compared to different patient samples and normal pre-B cells. Each dot indicates an individual patient sample. Data are taken from an ECOG E2993 clinical trial (Geng et al., 2012). Dark red asterisk (^∗^) indicates a significant difference compared to MLL-AF4, and pink asterisk (^∗^) indicates a significant difference compared to the MLL-FP group (which includes MLL-ENL [6], MLL-AF9 [1], and MLL-EPS15 [1]). ^∗∗∗^p < 0.001, ^∗∗^p < 0.01, ^∗^p < 0.05. A two-tailed Wilcoxon test was used to calculate p values, and p values for the different comparisons are listed in Table S5. See also Figures S4 and S5.

Because spreading is an important feature of MLL-AF4 binding, we investigated how common spreading is for other MLL-FPs. MLL(N) ChIP-seq in the MLL-AF6 cell line ML-2 detects the fusion protein unambiguously due to a deletion of the wild-type *MLL* allele. Spreading for MLL-AF6 was observed at 47 (43.1%) gene target isoforms (Figure 4A; Table S3), and similar to MLL-AF4, these spreading targets displayed a significant increase in H3K79Me2 compared to non-spreading MLL-AF6 targets (Figure 4B). The high percentage of spreading peaks within the MLL-AF6 set is due to the low number (109) of total MLL-AF6 binding events in ML-2 cells. Using MLL(N) ChIP-seq, spreading was also observed in MV4;11 (MLL-AF4), KOPN-8 (MLL-ENL), and THP-1 (MLL-AF9) MLLr cell lines (Figures 4C and 4D; Table S1). ER-tagged MLL-ENL (Garcia-Cuellar et al., 2016), biotin-tagged MLL-AF9 (Bernt et al., 2011), MLL-AF4 in MV4;11 cells (Zhu et al., 2016), MLL-AF4 in patient cells (this study), and FLAG-MLL-Af4 in CD34^+^ cells (Lin et al., 2016) also displayed spreading (Figures 4E, 4F, and S4A–S4E). The spreading pattern of MLL-AF4 in SEM cells often closely resembles the spreading pattern of MLL-AF4 in patient cells or in FLAG-MLL-Af4 cells at common gene targets (Figures S4D and S4E), suggesting there may be a common mechanism for spreading among these diverse samples.

Importantly, wild-type MLL spreading is not observed in non-MLLr leukemia cell lines (RCH-ACV or CCRF-CEM), at wild-type MLL binding sites in SEM cells (Figures 4C and 4D), or for wild-type MLL(C) in MV4;11 cells (Zhu et al., 2016) (Figure S4C). MLL-AF4 displays spreading in primary patient cells, but there was no wild-type MLL spreading in the relevant normal human hematopoietic cells from either cord blood (CB) or second-trimester fetal bone marrow (FBM) (Figures 4E, 4F, S4A, and S4D). These results show spreading is specifically associated with MLL-FPs.

### Some Individual Spreading Gene Targets Have Altered Gene Expression, Reduced DNA Methylation Patterns, and Individual Poor Prognoses in Patients

To better understand the clinical significance of individual spreading targets, we analyzed nine common targets from five MLLr leukemia cell lines (Figure 4G; Tables S1 and S4). High expression of *ARID2*, *JMJD1C*, *MBNL1*, *MEF2C*, or *RUNX2* alone is associated with at least one indicator of poor prognosis in ALL patients (Table S4), and *CPEB2*, *MBNL1*, *RUNX2*, and *ZEB2* are all specifically overexpressed in MLL-FP leukemias (Figures 4H and S5A; Table S5). Interestingly, *RUNX2*, *MBNL1*, *JMJD1C*, *SENP6*, *MEF2C*, and *ZEB2* are also hypomethylated in MLL-FP samples compared to either normal cells or other leukemias (Figure S5B; not shown). Although these data show that some MLL-FP spreading targets can individually have an important role in human leukemias, there does not seem to be a single key set of spreading targets that are necessarily found in all MLL-FP samples. However, taken in total, the data show that MLL-FP spreading is an indicator of particularly significant MLL-FP activity.

### Spreading Correlates with Menin and MLL-AF4 Complex Components

**Figure 5:**
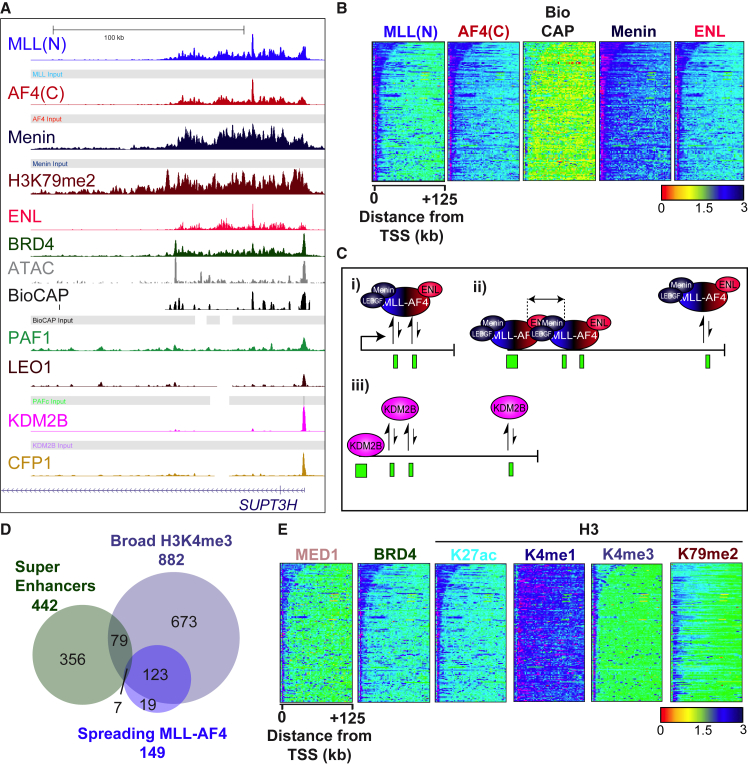
Spreading Correlates with Members of the Menin:LEDGF and Super-Elongation Complexes (A) Example ChIP-seq tracks at *SUPT3H* in SEM cells. (B) Heatmap of MLL-AF4, Bio-Cap, Menin, and ENL signal at all 149 spreading MLL-AF4 targets, ordered by length of spreading peak. Scale bar represents tags per bp per 10^7^ reads. (C) Schematic showing a proposed model for spreading across uCpG regions by MLL-FPs. (i) In the absence of promoter-bound MLL-AF4, CXXC-mediated recruitment of the fusion protein to uCpG-poor regions in the gene body are not stabilized. (ii) Stable CXXC-mediated recruitment to uCpG-rich promoter regions can stabilize nearby MLL-AF4 recruitment at gene body uCpG regions due to common interactions with complex members such as Menin and ENL, whereas distal recruitment events remain unstable. (iii) Because other CXXC proteins, such as KDM2B, do not interact with complex member such as Menin or ENL, promoter-bound KDM2B is not sufficient to stabilize neighboring CXXC-mediated recruitment to CpG-poor uCpG regions in the gene body. (D) Venn diagram showing the overlap between gene targets of super-enhancers, broad H3K4me3 peaks, and spreading MLL-AF4, in SEM cells. (E) Heatmap showing ChIP-seq reads of the components indicated at all 149 spreading MLL-AF4 gene targets in SEM cells; scale bar as in (B). See also Figure S6.

To better understand MLL-FP spreading, we analyzed whether spreading is related to other MLL-AF4 complex components (Figure 5A). When all MLL-AF4 spreading targets were sorted by length, the ChIP-seq signal of MLL(N) and AF4(C) generated a characteristic curve shape (Figure 5B, panels 1 and 2). Interestingly, MLL-AF4 spreading is punctuated by uCpG sites, with the beginning and end of spreading domains demarcated by uCpG sites (Figures 5A and 5B, panel 3). This indicates a role for the CXXC domain in stabilizing spreading and agrees with the hypomethylation observed at spreading MLL-AF4 targets in patients (Figure S5B). This is an important role for the CXXC domain within the context of MLL-AF4 because neither KDM2B nor CFP1 showed the same spreading pattern, even though they both bind to uCpGs (Figures 5A and S6A). The majority of uCpG regions under spreading peaks were within 1–2 kb of each other and rarely exceeded 4 kb, with 7 kb being the greatest distance observed (Figure S6B). Therefore, the proximity of uCpG sites to each other under the spreading peaks appears to be important and may be a limiting factor in determining the degree of spreading. If true, this also suggests that spreading may be non-random, and only genes with a clustered uCpG landscape downstream of their promoter are amenable to spreading.

Spreading was not simply a result of an association with basal transcription factors because neither RNAPII nor members of PAFc showed the same spreading pattern; instead, they extended beyond the spreading domain to the end of the gene (Figures 1C, 5A, and S6C). Conversely, both Menin and ENL displayed identical spreading patterns to MLL-AF4 (Figures 1C, 5A and 5B, panels 4 and 5). Our observation that Menin knockdowns reduce MLL-AF4 binding at spreading gene targets (Figures 2E–2G) supports the idea that there is a role for Menin in stabilizing spreading. In conclusion, we envisage a model whereby CXXC-mediated weak binding of the fusion protein at low-CG density uCpG sites in the gene body can be stabilized by CXXC-mediated recruitment to the CG-rich uCpGs at promoters (Figure 5C). However, this depends on the weak binding sites occurring in close proximity to the promoter and each other, with Menin or ENL facilitating stabilization through a bridging mechanism (Figure 5C).

### Spreading MLL-AF4 Represents a Subset of Broad H3K4me3 Distinct from Super-Enhancers

Several recent studies have characterized broad binding chromatin domains as markers of functional significance, including super-enhancers (Lovén et al., 2013, Whyte et al., 2013) and broad regions of H3K4me3 (Benayoun et al., 2014). Whereas genes associated with super-enhancers were shown to correlate with increased expression, genes marked by broad H3K4me3 showed an increase in transcriptional consistency, i.e., less variation in transcription rate between replicates as determined by RNA-seq and nascent RNA-seq, as well as an increase in gene expression (Benayoun et al., 2014). Here, we wanted to determine whether MLL-AF4 spreading domains were related to either super-enhancers or broad H3K4me3 peaks.

First, we characterized super-enhancers and broad H3K4me3 domains in SEM cells using the same criteria as the original studies (Figures S6D–S6G). Almost all regions of spreading MLL-AF4 were distinct from super-enhancers, but the majority (87%) of spreading MLL-AF4 gene targets were a subset of broad H3K4me3 gene targets (Figure 5D). Despite being distinct from super-enhancers, spreading MLL-AF4 correlated with MED1 and BRD4 binding as well as H3K27Ac (Figure 5E, panels 1–3). The major difference between MLL-AF4 spreading domains and super-enhancers was the lack of H3K4me1 enrichment; instead, spreading overlaps with H3K4me3 and H3K79me2 (Figure 5E, panels 4–6).

Similar to past work (Benayoun et al., 2014), gene targets of the 5% broadest H3K4me3 peaks in SEM cells showed a significant increase in transcriptional consistency compared to genes marked by the remaining 95% of H3K4me3 peaks (p < 0.0001, Mann-Whitney U test, Figure S6H). As a whole, gene targets of MLL-AF4 showed a significant increase in transcriptional consistency compared to H3K4me3-marked genes (p < 0.0001, Mann-Whitney U test; Figure S6I), suggesting that maintaining gene regulation within narrow limits could be an important property of MLL-AF4 controlled gene expression crucial for the leukemia. However, spreading MLL-AF4 gene targets did not show increased transcriptional consistency compared to all MLL-AF4 gene targets (Figure S6I); thus, this was not a feature specific to spreading. Based on the signature of histone marks and protein associations, spreading MLL-AF4 represents a hybrid of broad H3K4me3 domains and super-enhancers, with transcriptional properties such as high expression (Figure 3C) more similar to super-enhancers.

### Gene Targets of Spreading MLL-AF4 Display Increased Sensitivity to DOT1L Inhibition

**Figure 6:**
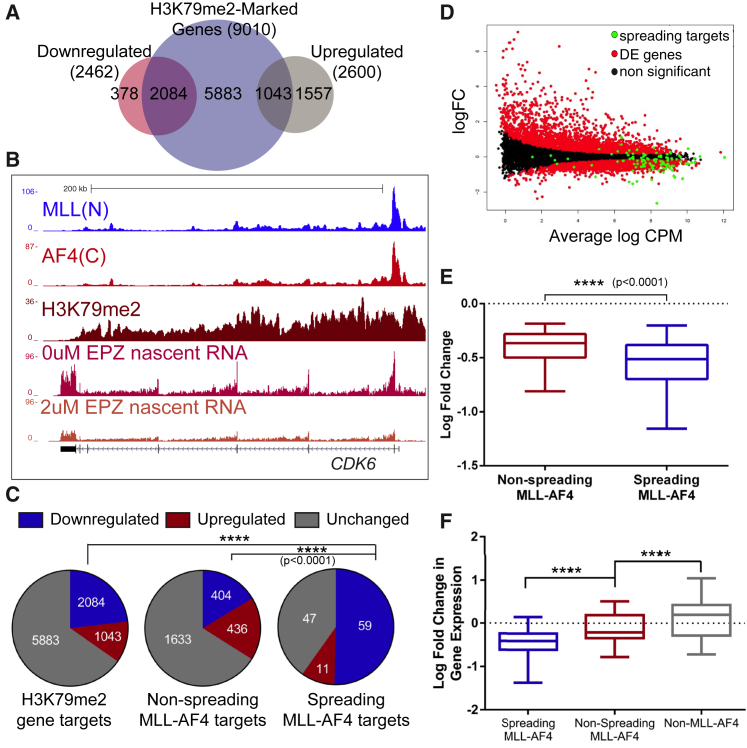
Spreading MLL-AF4 Targets Show Increased Sensitivity to DOT1L Inhibition (A) Venn diagram showing an overlap between H3K79me2-marked genes and upregulated and downregulated genes in SEM cells following treatment with 2 μM EPZ-5676. (B) Example ChIP-seq tracks at *CDK6* and nascent RNA-seq in control (0 μM) and 2 μM EPZ-5676-treated SEM cells. (C) Pie charts showing the proportion of genes that are significantly downregulated (blue), upregulated (red), or remain unchanged (gray), among H3K79me2-marked genes (left), non-spreading MLL-AF4 gene targets (center), and spreading MLL-AF4 gene targets (right), following treatment of SEM cells with 2 μM EPZ-5676. ^∗∗∗∗^p < 0.0001, Fisher’s exact test. (D) Smear plot showing the fold change in gene expression of all genes in SEM cells following treatment with 2 μM EPZ-5676 compared to their expression level (CPM). Black, non-significant change in gene expression; red, differentially expressed gene; green, spreading MLL-AF4 gene targets. (E) Box-and-whisker plot showing the median and IQ range of fold change in expression of all significantly downregulated gene targets of non-spreading MLL-AF4 (red) compared to spreading MLL-AF4 (blue), after 2 μM EPZ-5676 treatment in SEM cells. ^∗∗∗∗^p < 0.0001, Mann-Whitney U test. (F) Box-and-whisker plot showing the median and IQ range of fold change in expression of all significantly affected spreading MLL-AF4 (blue), non-spreading MLL-AF4 (red), and non-MLL-AF4 gene targets following siRNA-mediated knockdown of *MLL-AF4* in SEM cells. ^∗∗∗∗^p < 0.0001, Mann-Whitney U test. See also Figure S7.

If it is possible to target spreading MLL-AF4 target genes, it is likely that this would have a strong and specific effect on the inhibition of leukemia maintenance. Because spreading MLL-AF4 targets are marked with high levels of H3K79me2 (Figure 3D), we wanted to determine whether they are particularly sensitive to the DOT1L inhibitor EPZ-5676 (Daigle et al., 2013). Treatment of SEM cells with 2 μM EPZ-5676 for 7 days produced an almost complete loss of the H3K79me3 mark for all genes tested (Figure S7A). Using nascent RNA-seq, we identified 2,462 downregulated genes, 84% of which were marked by H3K79me2 (Figure 6A) and that included a number of spreading targets (e.g., *CDK6*; Figure 6B). As a group, over 50% of spreading targets were downregulated following EPZ-5676 treatment compared to only 16% of non-spreading MLL-AF4 targets and 23% of H3K79me2-marked genes (p < 0.0001, two-tailed Fisher’s exact test; Figure 6C). Furthermore, spreading MLL-AF4 targets were among those that showed the greatest downregulation, even compared to genes that had similar levels of high expression (Figure 6D). Spreading MLL-AF4 gene targets were also significantly more downregulated in response to EPZ-5676 compared to non-spreading MLL-AF4 gene targets (p < 0.0001, Mann-Whitney U test; Figure 6E). Therefore, spreading MLL-AF4 targets are among the most sensitive to treatment with EPZ-5676 when compared to all other genes. Using a randomly selected group of genes with levels of H3K79me2 similar to those of spreading MLL-AF4 targets (Figure S7B), we also found that spreading MLL-AF4 targets were significantly more downregulated after EPZ-5676 treatment (Figure S7C, top and bottom; p < 0.0001 and p < 0.01, respectively; Mann-Whitney U test). Therefore, genes marked by spreading MLL-AF4 show increased sensitivity to EPZ-5676 through a mechanism not simply determined by high levels of H3K79me2. Interestingly, spreading targets that overlapped with either broad H3K4me3 or super-enhancers were significantly more sensitive to DOT1L inhibition than spreading MLL-AF4 targets alone (Figure S7D). This indicates that there are further subdivisions of activity within spreading targets themselves, something that may explain recent results looking at DOT1L and BRD4 cooperation (Gilan et al., 2016).

As well as being particularly sensitive to a loss of H3K79me2/3, spreading MLL-AF4 gene targets were significantly downregulated compared to non-spreading and non-MLL-AF4 targets by MLL-AF4 siRNA treatment (p < 0.0001, two-tailed Mann Whitney U test; Figure 6F). Therefore, the increased gene expression observed at MLL-AF4 spreading targets is significantly linked to both MLL-AF4 and H3K79me2 and is more likely to be downregulated by DOT1L inhibition.

### Sensitivity of Spreading Gene Targets Provides a Rationale for Combination Therapy Using DOT1L Inhibitors

**Figure 7:**
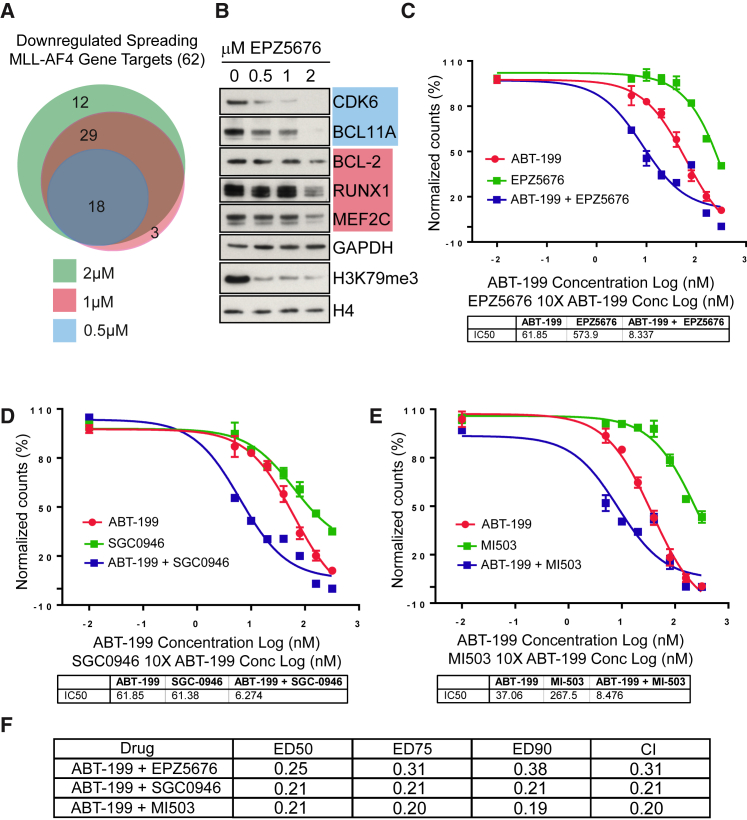
Sensitivity of Spreading Gene Targets Provides a Rationale for Combined Therapy Using DOT1L Inhibitors (A) Venn diagram showing the overlap of spreading MLL-AF4 gene targets that are downregulated as measured by nascent RNA-seq following 0.5 μM (blue), 1 μM (red), and 2 μM (green) EPZ-5676 treatment. (B) Western blot showing the protein expression of several spreading MLL-AF4 targets and controls in the presence of control, 0.5, 1, or 2 μM EPZ-5676 treatments. Blue and red boxes relate to treatment colors in (A) that led to the lowest level of treatment that resulted in reduced gene transcription. (C–E) A cell viability assay of SEMK2 cells treated with a DMSO control, different concentrations of ABT-199 (320, 160, 80, 40, 20, 10, and 5 nM, and DMSO control) alone, or in combination with a 1:10 ratio of either EPZ5676 (C), SGC0946 (D), or MI503 (E) (3,200, 1,600, 800, 400, 200, 100, and 50 nM, and DMSO control). (F) A tabular summary of the combination index for the different drug treatments calculated as in Milella et al. (2002).

It seems unlikely that a single drug alone will be effective in treating MLL-AF4 leukemias. Even among MLL-AF4 spreading targets, some gene targets have an increased sensitivity to a loss of H3K79me2 (Figures 7A and 7B; Table S6). We recently showed that the BCL-2-specific protein inhibitor ABT-199 synergizes with DOT1L inhibitors (Benito et al., 2015), potentially because BCL-2 protein levels are not strongly affected by DOT1L inhibitor concentrations that affect more sensitive targets such as CDK6 or BCL11A (Figure 7B). Because Menin is partly responsible for spreading (see Figures 2E–2G and 5A–5C), the use of Menin inhibitors represents another way to target sensitive MLL-AF4 spreading genes (see Figure S7E). In an extension of our earlier work, we find that there is a strong synergy between ABT-199 and the DOT1L inhibitors EPZ5676 and SGC0946, as well as a strong synergistic interaction between the Menin inhibitor MI-503 (Borkin et al., 2015) and ABT-199 (Figures 7C–7F; combination index [CI] < 1; calculations as described in Milella et al. [2002]). Thus, carefully choosing different drug combinations may increase their effectiveness at disrupting MLL-FP leukemic growth.

## Discussion

In this study, we have found that uCpGs strongly correlate with the highest occupancy of MLL-AF4 binding and that MLL-AF4 and Menin co-stabilize each other’s binding to gene targets. This mirrors recent findings of co-dependent stabilization between wild-type MLL and LEDGF (Zhu et al., 2016), which would suggest an independent role for LEDGF in wild-type MLL function, as previous reports indicate that Menin and wild-type MLL regulate distinct gene targets (Li et al., 2013). Zhu et al. have also shown that loss of LEDGF actually increases MLL-FP recruitment (Zhu et al., 2016), which is partly supported by our *Ledgf* knockdown experiment (Figure S2G). We have also shown that there is no direct connection between MLL-AF4 recruitment and PAFc, suggesting that past observations of MLL-FP dependence on PAFc (Milne et al., 2010, Muntean et al., 2010) may have been due to indirect effects, or perhaps PAFc is only required for binding of MLL-FPs at specific gene targets such as *HOXA9*.

Previous studies have indicated that MLL-FP binding can be associated with broad chromatin domains (Bernt et al., 2011, Guenther et al., 2008) or divided into two classes, with 5′ binding indicating a dependence on H3K79me2 (Garcia-Cuellar et al., 2016). Our results suggest that it is not the presence of MLL-AF4 and H3K79me2/3 that is most predictive of a dependence on H3K79me2, but the presence of MLL-AF4 spreading. Our observations also show that spreading strongly correlates with Menin and ENL binding and occurs across uCpG landscapes in the gene body that are within close proximity to the gene promoter. Unmethylated CpG regions in gene bodies typically display a relatively low CG density, which is possibly why we do not observe other CXXC proteins binding in the gene body. Therefore, spreading of MLL-FPs may be made possible by Menin/ENL-mediated stabilization at gene body uCpG regions where uCpG density is too low for a strong CXXC-uCpG interaction. Although wild-type MLL can also interact with Menin, a co-operation with fusion partner proteins such as ENL may generate complexes that permit MLL-FP dimerization (Mueller et al., 2007, Yokoyama et al., 2010) and through this mechanism create a spreading domain of MLL-FP that is anchored by CXXC-uCpG interactions in close proximity to each other, something that is unavailable to wild-type MLL.

It is unknown whether an ability to spread into the body of particular gene targets drives higher expression and initiates progression of leukemia or whether these gene targets are already highly expressed and the active chromatin landscape is simply a pre-requisite for facilitating spreading. Nevertheless, in the context of these remaining questions, our study has revealed that spreading of MLL-AF4 defines the expression of a subset of genes that are important for leukemia and are characterized by gene activation that is predictive of a poor prognosis. These target genes are particularly sensitive to DOT1L inhibition, which provides a new molecular rationale for the specificity of DOT1L or Menin inhibition in MLL-AF4 leukemias, and the possibility of combining this with drugs that target less sensitive spreading targets such as those that target BCL-2.

## Experimental Procedures

### Cell Lines, Cultures, and Drug Treatment Studies

Cell lines, culture methods, and drug treatment protocols used in this study are listed in Supplemental Experimental Procedures. CB was collected under the auspices of a National Research Ethics Service-approved study with written informed consent. Human fetal bone samples were obtained through the Human Development Biology Resource (http://www.hdbr.org).

### Western Blot Analysis

Western blot analysis was performed as previously described (Wilkinson et al., 2013). Antibodies used for western blot analysis are listed in Supplemental Experimental Procedures.

### ChIP Assays and ChIP-Seq

ChIP and ChIP-seq experiments were performed as described in Supplemental Experimental Procedures and as previously described (Benito et al., 2015, Wilkinson et al., 2013).

### TetR Recruitment System

For the TetR recruitment assay, we used the previously engineered Tet-operon (TetO) mESC line (Blackledge et al., 2014). *MLL-AF4*, *Menin*, and *PAF1* cDNA were inserted downstream of the FS2-TetR coding sequence in the original pCAGFS2TetR vector, by ligation-independent cloning (LIC). Plasmids were transfected into TetO mESCs using Lipofectamine 2000, and clones stably expressing TetR fusions were selected using puromycin (1 μg/mL), or *MLL-AF4* cDNA was transiently transfected into mESCs at 60%–70% confluency using Lipofectamine 2000. Cells were collected 24 hr after transfection.

### Gene Targets and Spreading Peaks

ChIP-seq peaks were called as described in Supplemental Experimental Procedures. Gene targets were defined as any gene where the transcription start site (TSS) overlapped directly with a peak. A peak was classed as spreading if it overlapped the TSS of a gene and extended over 4 kb from the TSS into the gene body without going beyond the transcription end site (TES) of the gene. If a peak exhibited spreading at two different genes (or isoforms with different TSS co-ordinates), both spreading-gene pairs were kept.

### Nascent RNA-Seq

Nascent RNA-seq and gene expression analysis was performed as described in Supplemental Experimental Procedures.

### Survival Analysis

Clinical datasets and survival analyses are detailed in Supplemental Experimental Procedures.

### Statistics

Data were analyzed using Fisher’s exact test, Wilcoxon test, and Mann-Whitney U test, where appropriate. Results were deemed significant if p < 0.05. Unless otherwise indicated, data are shown as mean ± SD. This paper analyzed datasets from GEO: GSE13313, GSE28460, GSE29130, GSE34861, GSE73528, GSE74812, and GSE84116; and ArrayExpress: E-MTAB-3593 (for a detailed list of datasets, see Table S7).

## Author Contributions

Conceptualization, T.A.M. and J.K.; Formal analysis, J.K., E.R., H.G., and H.M.; Investigation, J.K., L.G., M.T., N.P.B., H.M., E.B., T.A.M.; Resources, S.O., F.P., O.H., A.R., I.R.; Writing – Original draft, T.A.M. and J.K.; Writing – Review & Editing, T.A.M., J.K., O.H., A.R., I.R., M.K., R.J.K., H.G.; Visualization, T.A.M. and J.K.; Supervision, T.A.M., M.K., and R.J.K.; Funding Acquisition, T.A.M., O.H., A.R., I.R., R.J.K., M.K.

## Accession Numbers

The accession number for the datasets reported in this paper is GEO: GSE83671.

## Supplemental Information

Document S1. Supplemental Experimental Procedures, Figures S1–S7, Tables S2–S4, S6, and S7

Table S1. MLL-FP Gene Target Lists, Related to Figures 1 and 4

Table S5. P Values for Patient Gene Expression Data, Related to Figures 4 and S5

Document S2. Article plus Supplemental Information
