# Supplementary material for: MLL-AF4 Spreading Identifies Binding Sites that Are Distinct from Super-Enhancers and that Govern Sensitivity to DOT1L Inhibition in Leukemia
Source: Cell Rep. 2017 Jan 10;18(2):482–95. doi: 10.1016/j.celrep.2016.12.054 (PMC5263239; doi:10.1016/j.celrep.2016.12.054)
Supplement: Document S1. Supplemental Experimental Procedures, Figures S1–S7, Tables S2–S4, S6, and S7 [file mmc1.pdf]

Cell Reports, Volume *18*

Supplemental Information

MLL-AF4 Spreading Identifies Binding Sites that Are Distinct from Super-Enhancers and that Govern Sensitivity to DOT1L Inhibition in Leukemia

Jon Kerry, Laura Godfrey, Emmanouela Repapi, Marta Tapia, Neil P. Blackledge, Helen Ma, Erica Ballabio, Sorcha O'Byrne, Frida Ponthan, Olaf Heidenreich, Anindita Roy, Irene Roberts, Marina Konopleva, Robert J. Klose, Huimin Geng, and Thomas A. Milne

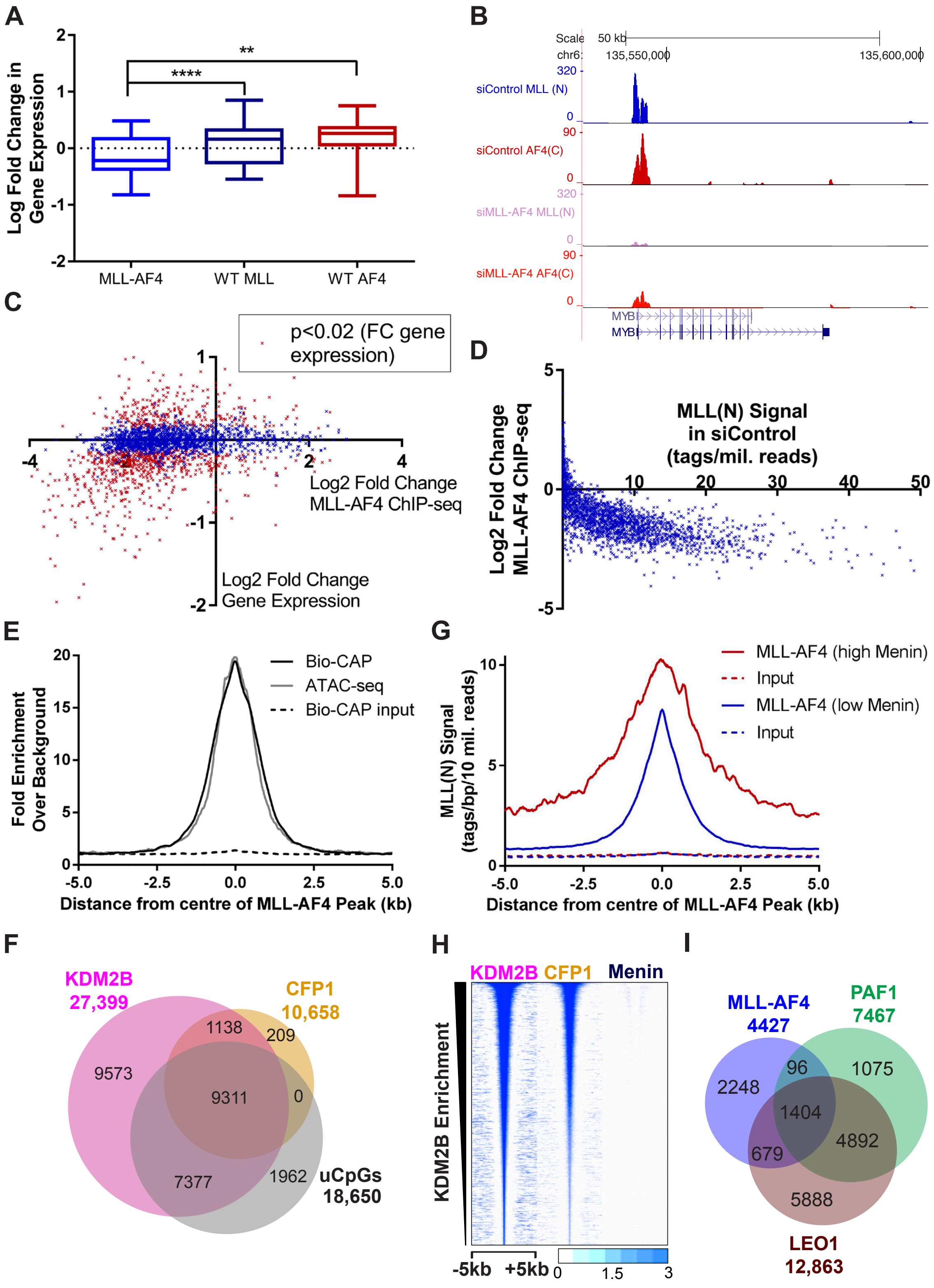

Figure S1, MLL-AF4 recruitment, Related to Figure 1. (**A**) Box and whisker plot showing the median and IQ range of fold change in gene expression, measured by nascent RNA-seq, of all significantly affected gene targets of MLL-AF4 (MLL(N) ChIP-seq peaks that overlap with AF4(C) peaks), wild-type MLL1 (MLL(N) peaks that do not overlap with AF4(C)) and wild-type AF4 (AF4(C) peaks that do not overlap with MLL(N)), following treatment with an MLL-AF4-specific siRNA in SEM cells. (**B**) Example ChIP-seq tracks showing MLL(N) and AF4(C) in SEM cells treated with either control siRNA (top two lanes), or MLL-AF4-specific siRNA (bottom two lanes). (**C**) Scatter plot showing the log2 fold-change in MLL-AF4 ChIP-seq (mean average fold-change of MLL(N) + AF4(C)) and log2 fold change in gene expression following treatment of SEM cells with MLL-AF4-specific siRNA, at all MLL-AF4 gene targets. Red points show genes that had a significant ($p<0.02$) fold-change in gene expression. (**D**) Scatter plots showing how log2 fold-change in MLL-AF4 ChIP (same data as in (C)) is related to the amount of MLL(N) signal at MLL-AF4 gene targets in SEM cells treated with control siRNA. (**E**) Composite binding plot of uCpG (Bio-CAP and ATAC) reads at MLL-AF4 binding sites in SEM cells. (**F**) Venn diagram showing the overlap between KDM2B and CFP1 binding sites with uCpG regions (Bio-CAP/ATAC-seq), in SEM cells. (**G**) Composite binding plot showing MLL(N) ChIP-seq reads at MLL-AF4 binding sites that overlap with high levels of Menin ChIP-seq reads (red) or low levels of Menin ChIP-seq reads (blue), in SEM cells. (**H**) Heat-map showing ChIP-seq reads of KDM2B, CFP1 and Menin at all 27,399 KDM2B binding sites in SEM cells. Scale bar represents tags per bp per 107 reads. (**I**) Venn diagram showing the overlap between MLL-AF4 and PAFc (PAF1 and LEO1) binding sites .

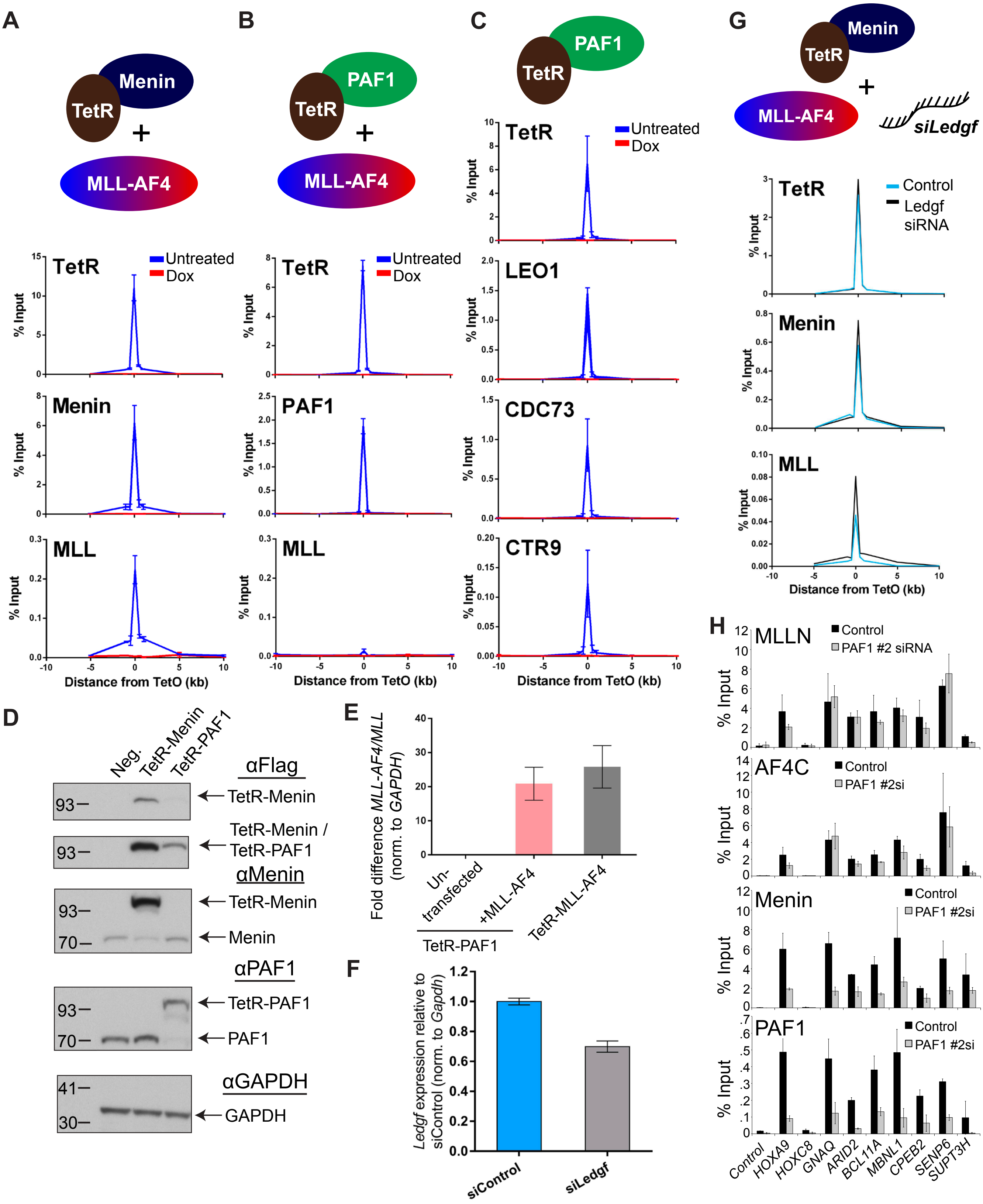

Figure S2, Menin but not PAF1 recruits MLL-AF4, Related to Figure 2. (**A**) ChIP-qPCR showing the binding of Menin and MLL-AF4 (MLL(N)) at the TetO array in TetR-Menin mES cells transfected with MLL-AF4, in the presence (red) or absence (blue) of doxycycline. (**B**) ChIP-qPCR showing the binding of PAF1 and MLL-AF4 in TetR-PAF1 mES cells transfected with MLL-AF4. (**C**) ChIP-qPCR showing the binding of the PAFc members LEO1, CDC73 and CTR9 at the TetO array in TetR-PAF1 mES cells. (**D**) Western blot showing the expression of TetR-Menin and TetR-PAF1 in TOT2N mES cells, stably expressing each protein. (**E**) cDNA levels of the human MLL-AF4 fusion gene after transfection of TOT2N mES cells with either MLL-AF4 or TetR-MLL-AF4, compared to untransfected cells. Error bars represent the standard deviation of 2 biological replicates. (**F**) cDNA levels of mouse Ledgf after transfection of TetR-Menin mES cells with either control siRNA or Ledgf siRNA, normalized to control. Error bars represent the standard deviation of 2 technical replicates. (**G**) ChIP-qPCR showing the binding of TetR-Menin and MLL-AF4 at the TetO array in TetR-Menin mES cells transfected with MLL-AF4 and Ledgf-specific siRNA (related to (F)). (H) MLL(N), AF4(C), Menin and PAF1 ChIP in SEM cells treated with either control (black bars) or PAF1#2 siRNAs (gray bars). Bars represent the average of two independent experiments and error bars = S.D. between experiments.

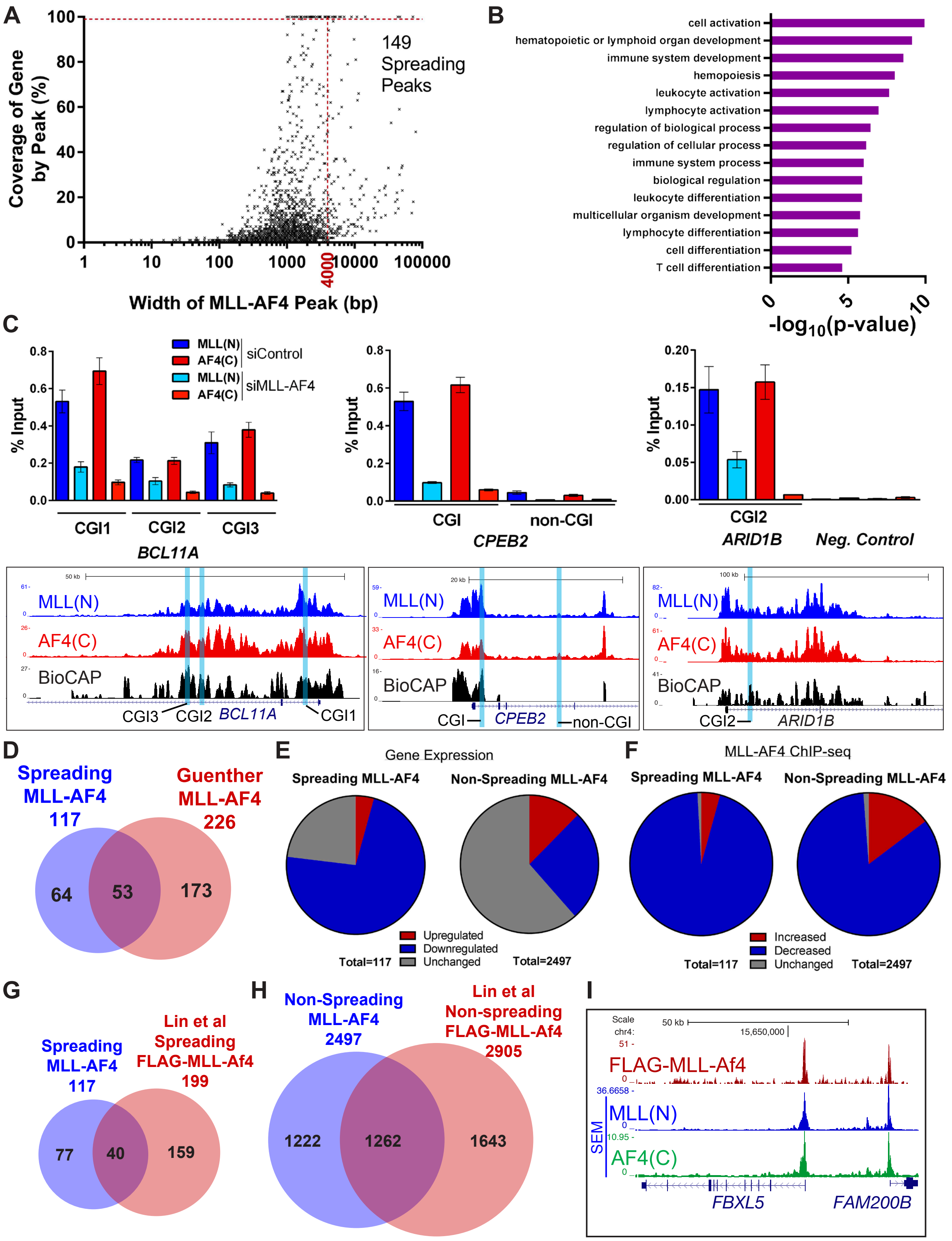

Figure S3, MLL-AF4 binding shows spreading, Related to Figure 3. (**A**) Spreading MLL-AF4 peaks were defined as peaks that extend greater than 4kb from the TSS into the gene body without going beyond the end of the gene. Using these criteria, 149 spreading MLL-AF4 peaks were identified in SEM cells. (**B**) Gene ontology analysis for all gene targets of spreading MLL-AF4 in SEM cells. (**C**) Spreading MLL-AF4 at specific gene targets confirmed using MLL-AF4 siRNA treatment followed by ChIP-qPCR of MLL(N) and AF4(C) (top). ChIP-seq tracks (bottom) show locations of the primers used. (**D**) Venn diagram showing the overlap between spreading MLL-AF4 gene targets in SEM cells and all MLL-AF4 targets identified in SEM cells by Guenther et al. (Guenther et al., 2008). (**E**) Pie charts showing the proportion of spreading MLL-AF4 and non-spreading MLL-AF4 gene targets in SEM cells that are either significantly ($p<0.02$) upregulated (red), downregulated (blue) or unchanged (grey), following treatment with MLL-AF4-specific siRNAs. Data represents nascent RNA-seq from 3 biological replicates. (**F**) Pie charts showing the proportion of MLL-AF4 peaks at spreading and non-spreading gene target promoters in SEM cells that show an increase (red), decrease (blue) or no change (grey) in MLL-AF4 ChIP-seq signal following MLL-AF4c siRNA treatment. (**G-H**) Venn diagrams showing the overlap between spreading and non-spreading MLL-AF4 gene targets in SEM cells compared to a FLAG-MLL-AF4 ChIP-seq dataset from CD34+ cord blood cells (Lin et al., 2016). (**I**) Example ChIP-seq tracks showing non-spreading at FBXL5 and FAM200B in both MLL-AF4 SEM cells and FLAG-MLL-Af4 cord blood cells (Lin et al., 2016).

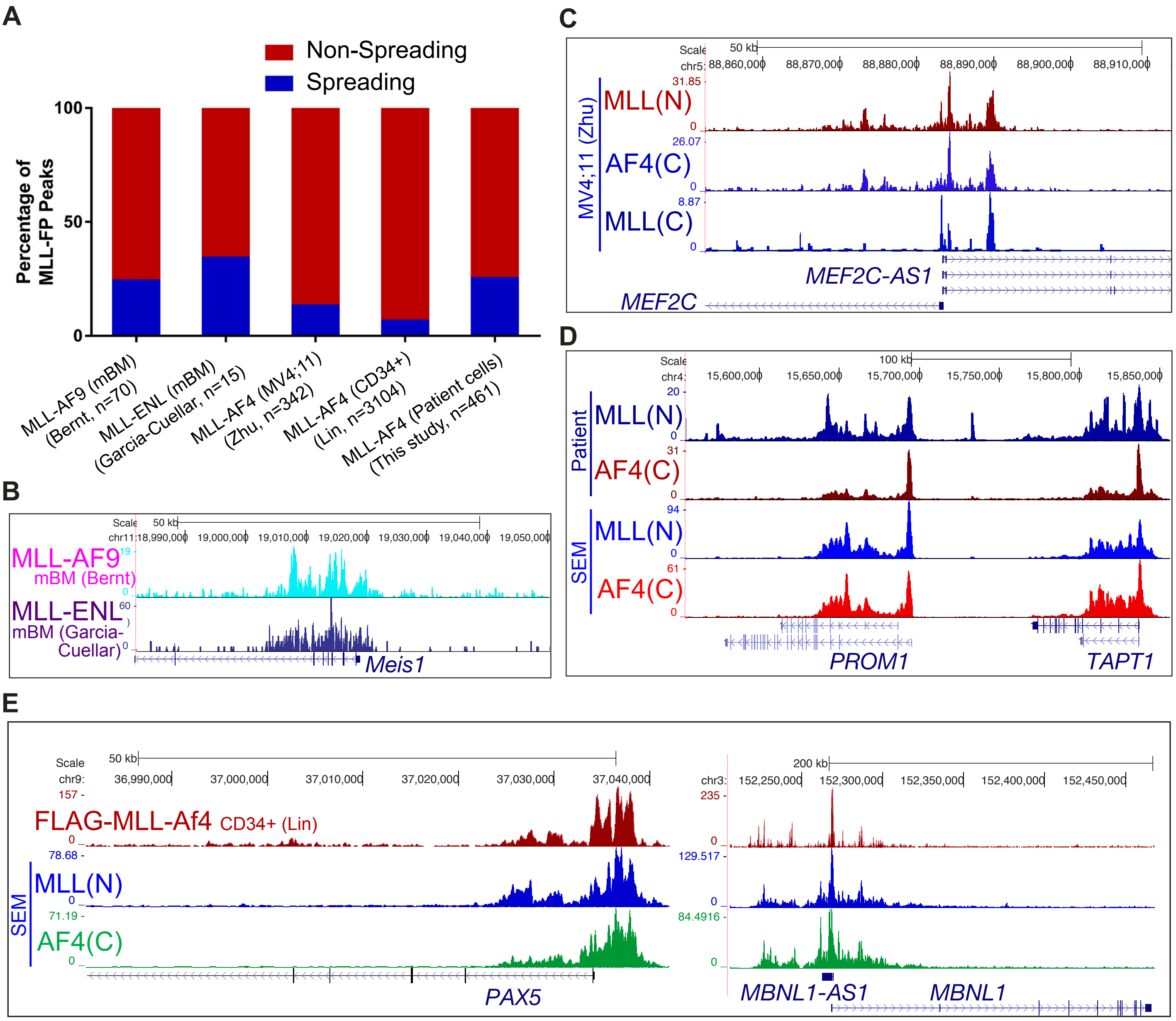

Figure S4, MLL-FPs display spreading but wild type MLL does not, Related to Figure 4. (**A**) Bar plot showing the percentages of spreading and non-spreading peaks within MLL-FP protein peaks in previously published datasets: MLL-AF9 (Bernt et al., 2011), MLL-ENL (Garcia-Cuellar et al., 2016) and MLL-AF4 (Zhu et al., 2016), as well as amongst MLL-AF4 peaks in patient cells. (**B**) Example ChIP-seq tracks showing spreading MLL-AF9 and spreading MLL-ENL at Meis1 in mouse bone marrow cells (datasets same as in (A)). (**C**) Example ChIP-seq tracks showing spreading MLL-AF4 (MLL(N) and AF4(C)) at MEF2C in MV4;11 cells (dataset same as in (A)). MLL(C) was used to show the corresponding lack of spreading by wild-type MLL. (**D**) Example ChIP-seq tracks showing spreading MLL-AF4 (MLL(N) and AF4(C)) at PROM1 and TAPT1 in both MLL-AF4 patient cells and SEM cells. (**E**) Example ChIP-seq tracks comparing spreading MLL-AF4 (MLL(N) and AF4(C)) at PAX5 and MBNL1 in both MLL-AF4 SEM cells and FLAG-MLL-Af4 cord blood cells (Lin et al., 2016).

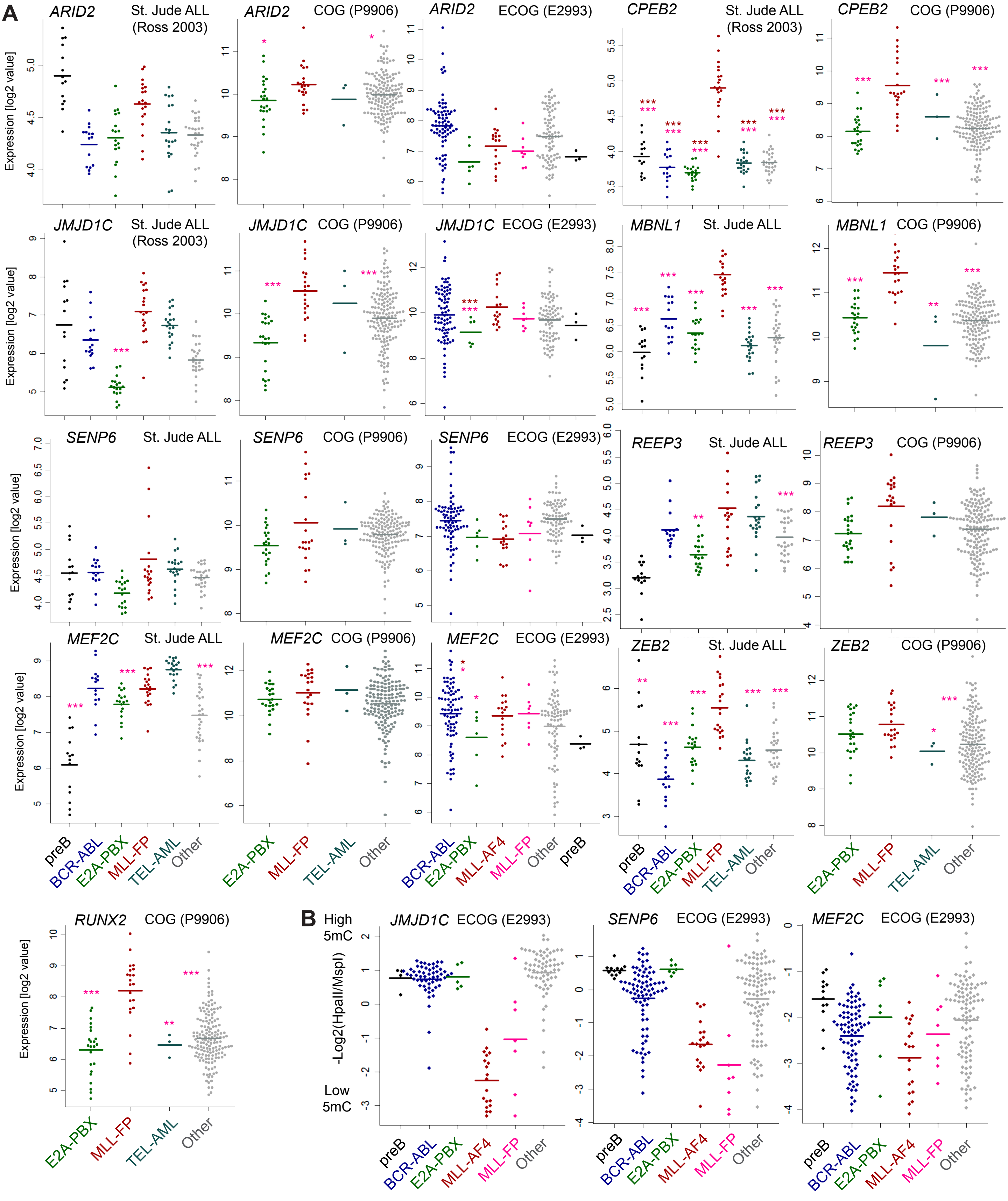

Figure S5, Expression and DNA methylation of spreading targets in patients, Related to Figure 4. (**A**) Expression of the genes indicated in patient samples from three large cohorts of patients with ALL including the Eastern Cooperative Oncology Group (ECOG) Clinical Trial E2993, the Children's Oncology Group (COG) Clinical Trial P9906 and the St. Jude Research Hospital pediatric ALL clinical trial cohort. Details of each trial are listed in supplemental methods. Dark red * indicates a significant difference compared to MLL-AF4, pink * indicates a significant difference compared to the MLL-FP group, no * indicates that the gene is not significantly upregulated in MLL-FP samples. *** = p<0.001, ** = p<0.01, * = p<0.05. All p-values are listed in Table S5. (**B**) DNA methylation data (collected using the HELP assay) for JMJD1C, SENP6 and MEF2C from the (ECOG) Clinical Trial E2993.

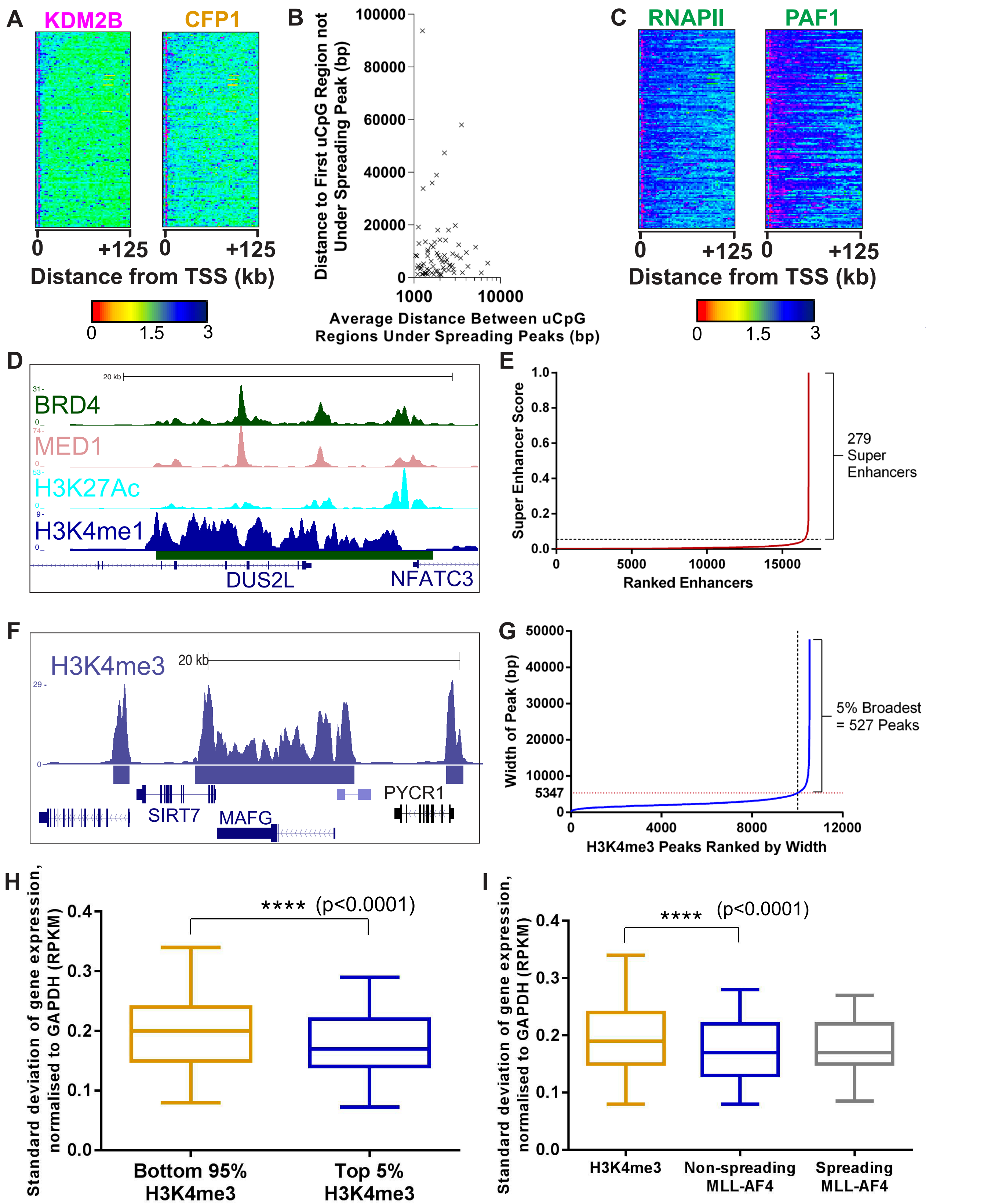

Figure S6, Super enhancers and Broad H3K4Me3 domains, Related to Figure 5. (**A**) Heat-map showing KDM2B (left) and CFP1 (right) ChIP-seq reads at all 149 spreading MLL-AF4 peaks in SEM cells. Scale bar represents tags per bp per 107 reads. (**B**) Scatter plot showing the distance in bp between uCpG regions (Bio-CAP-seq peaks) found under spreading MLL-AF4 peaks against the distance to the first adjacent uCpG region that is not under the spreading peak but found within the body of the same gene. (**C**) Heat-map showing RNA polymerase II (RNAPII) and PAF1 ChIP-seq reads at all 149 spreading MLL-AF4 peaks in SEM cells. Scale bar as in (A). (D) Example ChIP-seq track of a super enhancer at DUS2L in SEM cells. (E) Enhancers characterized by BRD4, MED1, H3K27ac and H3K4me1 in SEM cells were ranked by their super enhancer score. In total, 279 were classed as super enhancers. (F) Example ChIP-seq track of a broad H3K4me3 peak (i.e. one of the top 5% broadest H3K4me3 peaks) spanning SIRT7 and MAFG, in SEM cells. Two normal (non-broad) H3K4me3 peaks are shown either side. (G) All H3K4me3 peaks in SEM were ranked based on length and the top 5% were classified as "broad" H3K4me3. This identified 527 broad H3K4me3 peaks. (H) Box and whisker plot showing the median and IQ range of transcriptional consistency of genes marked by the 5% broadest H3K4me3 peaks (blue) compared to genes marked by the remaining 95% of H3K4me3 peaks (orange). ****=p<0.0001, two-tailed Mann-Whitney U test. (I) Box and whisker plot showing the median and IQ range of transcriptional consistency of gene targets of non-spreading MLL-AF4 (blue,) spreading MLL-AF4 (gray) as well as all genes marked by H3K4me3 (orange). ****=p<0.0001, two-tailed Mann-Whitney U test

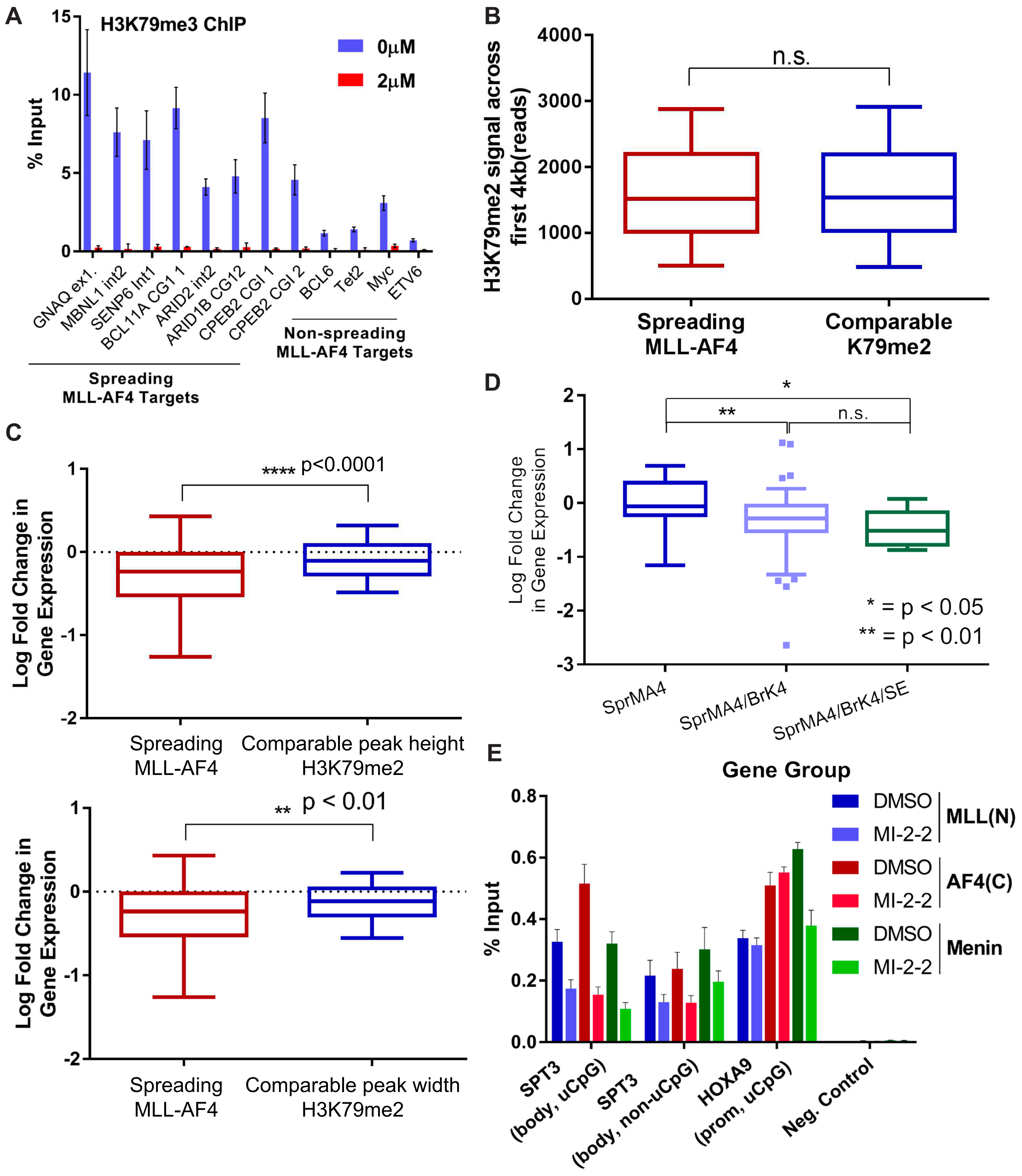

Figure S7, Spreading predicts sensitivity to loss of H3K79me2/3, Related to Figure 6. (**A**) ChIP-qPCR of H3K79me3 at spreading and non-spreading MLL-AF4 gene targets in SEM cells treated with either DMSO control or 2μM EPZ-5676, for 7 days. (**B**) Box and whisker plot showing the median and IQ ranges of H3K79me2 reads across the first 4kb of the gene body of (i) spreading MLL-AF4 targets (n = 117) and (ii) randomly selected genes with comparable levels of H3K79me2 to that of spreading MLL-AF4 targets (n =117). Spreading MLL-AF4 targets were omitted from the selection of genes for group (ii). N.s. = not significant, Mann-Whitney U test. (**C**) Box and whisker plot showing the median and IQ range of fold change in gene expression, measured by nascent RNA-seq, of genes in group (i) and (ii) from (B) following a 7-day treatment of EPZ-5676 (top) and comparing to similar peak width rather than peak height (bottom). ****=p<0.0001,**=p<0.01, Mann-Whitney U test. (**D**) Box and whisker plot showing the median and IQ range of fold change in gene expression, measured by nascent RNA-seq, for gene targets of just spreading MLL-AF4 (SprMA4), both spreading MLL-AF4 and broad H3K4me3 (SprMA4/BrK4), and spreading MLL-AF4, broad H3K4me3 and Super Enhancers (SprMA4/BrK4/SE) following a 7-day treatment with 2μM EPZ-5676. *=p<0.05, **=p<0.01, Mann-Whitney U test. (**E**) ChIP-qPCR of MLL(N), AF4(C) and Menin in SEM cells following treatment with either DMSO or 12.5μM MI-2-2 (Shi et al., 2012) for 48 hours at two loci in the MLL-AF4 spreading domain at SPT3 (one a uCpG region, left, and the other a non-uCpG region, right) and the uCpG promoter of HOXA9. Error bars represent the standard deviation of PCR replicates.

Table S1, (excel file) MLL-FP gene target lists, related to Figure 1 and Figure 4

Table S2, Spreading MLL-AF4 gene targets, Related to Figure 3 and Figure S3A.

Gene Name (117)	Gene Isoforms (149), RefSeq ID
ADAM10	NM_001110
AFF1	NM_001166693
ANP32A	NM_006305
APOLD1	NM_001130415
ARHGDIB	NM_001175
ARID1B	NM_020732
ARID2	NM_152641
ARPP21	NM_001267617; NM_016300; NM_001267619
ARRDC3	NM_020801
ATP8B4	NM_024837; NR_073598
BCL11A	NM_022893
BCL2	NM_000633
C5orf56	NR_045116
CACNB4	NM_001005746; NM_000726
CAMK2D	NM_172127
CCDC162P	NR_028595
CDCA7	NM_031942
CDK14	NM_012395
CDK6	NM_001145306; NM_001259
CELF2	NM_001083591
CHD2	NM_001271
CLEC2B	NM_005127
CLEC2D	NM_001197319
CLECL1	NM_001253750
CNST	NM_001139459
CPEB2	NM_001177383
CTBP2	NM_001083914; NM_001329
CXorf21	NM_025159
CXXC5	NM_016463
CYTIP	NM_004288
DGKD	NM_152879
DIAPH1	NM_005219
EBF1	NM_024007
ELOVL6	NM_024090; NM_001130721
ERG	NM_001136155
EVI2B	NM_006495
FLT3	NM_004119
FUT4	NM_002033
GNAQ	NM_002072
HIPK3	NM_001278163; NM_005734; NM_001278162
HIVEP2	NM_006734
HMGA2	NM_003483

HMGB1	NM_002128
HNRNPF	NM_001098205; NM_004966; NM_001098204
IER2	NM_004907
IGF1R	NM_000875
IKZF1	NM_001220767
JMJD1C	NM_032776; NM_004241
KLRC4-KLRK1	NM_001199805
KLRK1	NM_007360
LAT2	NM_032464
LEF1	NM_016269; NM_001166119
LEF1-AS1	NR_029374
LMO4	NM_006769
LOC728175	NR_040108
LRMP	NM_001204126
LRRFIP2	NM_001134369
MAFG	NM_032711
MAP3K1	NM_005921
MBNL1	NM_207297; NM_207292
MEF2C	NM_001193349; NM_002397
MEIS1	NM_002398
NR3C1	NM_001018076; NM_001204259
NUSAP1	NM_018454
PAN3	NM_175854
PAX5	NM_016734
PDE4DIP	NM_001002811
PHLPP1	NM_194449
PIK3CD	NM_005026
PKM	NM_001206798
PLEK	NM_002664
PPP2R5C	NM_001161726; NM_178586
PPP6R1	NM_014931
PROM1	NM_001145848; NM_001145847
PTEN	NM_000314
PTPN6	NM_002831
PTPRR	NM_001207015; NR_073474; NM_001207016
RCC1	NM_001048199
REEP3	NM_001001330
RHOH	NM_001278368; NM_004310; NM_001278367; NM_001278369
RNF19B	NM_153341
RNF220	NM_018150
ROBO1	NM_133631
RPSAP52	NR_026825
RUNX1	NM_001754; NM_001122607
RUNX2	NM_001015051; NM_001278478; NM_004348
SEMA3A	NM_006080

SENP6	NM_001100409
SMC4	NM_005496; NM_001002800
SOX11	NM_003108
SPEN	NM_015001
SPN	NM_001030288
SPRY4	NM_030964
ST8SIA4	NM_175052; NM_005668
STC2	NM_003714
STK17B	NM_004226
SUPT3H	NM_003599
SYK	NM_001135052; NM_001174168
SYT1	NM_001135805
TAPT1	NM_153365
TAPT1-AS1	NR_027697
TBC1D14	NM_001113361; NM_020773
TGFBR2	NM_001024847
TGIF1	NM_173209; NM_173208; NM_173207
TMPO	NM_001032284
TNRC18	NM_001080495
TPD52	NM_001025253
TRHDE	NM_013381
TRHDE-AS1	NR_026837
TSC22D2	NM_014779
UBASH3B	NM_032873
ZC3H12C	NM_033390
ZC3HAV1	NM_020119
ZCCHC6	NM_001185059
ZCCHC7	NM_032226
ZEB2	NM_014795
ZMYND8	NM_012408

Table S3, Spreading MLL-AF6 gene targets, Related to Figure 4A.

Gene Name (38)	Gene Isoforms (47), RefSeq ID
ADAMTS19	NM_133638
APOLD1	NM_001130415
ARID2	NM_152641
CD69	NM_001781
CDK13	NM_031267
CLEC2A	NM_001130711
CLEC2B	NM_005127
CPEB2	NM_001177382
DACH1	NM_004392
DLX6-AS1	NR_015448
EMB	NM_198449
FAM169A	NR_046462; NM_015566
FOXP1	NM_001012505; NM_001244808
FRY	NM_023037
JMJD1C	NM_032776
LOC646762	NR_024278
MBNL1	NM_021038
MEF2C	NM_001193349; NM_001193350
MYB	NM_001161659
MYO6	NM_004999
NPAS3	NM_001164749
PARP8	NM_001178055; NM_024615
PTPRK	NM_002844; NM_001135648
RBMS1	NM_002897
REEP3	NM_001001330
RNF220	NM_018150
RUNX2	NM_004348; NM_001278478
SATB1	NM_001131010; NM_002971
SENP6	NM_015571
SSPN	NM_005086; NM_001135823
SUPT3H	NM_181356
SYDE2	NM_032184
TAPT1-AS1	NR_027697
TCF4	NM_001243226; NM_001243230
TCTEX1D1	NM_152665
TRPS1	NM_014112
ZEB2	NM_014795
ZNF521	NM_015461

Table S4, Gene targets common to 5 spreading MLL-FPs, Related to Figure 4G.

**Gene Name (9)**	**Gene Isoforms (10), RefSeq ID**	**MRD (COG)**	**OS (COG or ECOG)**	**Relapse**
ARID2	NM_152641		poor	
CPEB2	NM_001177382			
JMJD1C	NM_032776			✔
MBNL1	NM_021038	✔		✔
MEF2C	NM_001193349			
REEP3	NM_001001330			
RUNX2	NM_004348; NM_001278478	✔		✔
SENP6	NM_015571			
ZEB2	NM_014795			

Table S5, (excel file) P-values for patient gene expression data, Related to Figure 4 and Figure S5

Table S6, Spreading MLL-AF4 gene targets downregulated at different concentrations of EPZ-5676, Related to Figure 7A.

1µM unique	2µM unique	1µM & 2µM overlap	05µM, 1µM & 2µM overlap
TPD52	STC2	LEF1	CPEB2
SENP6	CNST	RUNX1	PROM1
LRRFIP2	PHLPP1	SPRY4	AFF1
	RNF220	JMJD1C	ERG
	TNRC18	LEF1-AS1	ARHGDIB
	ANP32A	BCL2	HMGA2
	SOX11	CDCA7	DIAPH1
	PPP6R1	ZMYND8	BCL11A
	PKM	CACNB4	ZC3HAV1
	APOLD1	ZC3H12C	UBASH3B
	PAX5	ELOVL6	ARPP21
	IER2	HIPK3	CTBP2
		TSC22D2	HIVEP2
		SUPT3H	TGFBR2
		TRHDE	ARID1B
		SYT1	CDK6
		PPP2R5C	IKZF1
		SPEN	IGF1R
		HNRNPF	
		MEF2C	
		ARID2	
		TBC1D14	
		CAMK2D	
		CELF2	
		TAPT1	
		TRHDE-AS1	
		MAFG	
		ZCCHC7	
		CDK14	

Table S7, Datasets from previous publications, Related to Figures 1 and 3-6

ChIP-seq

Cell Line	Antibody	Accession Number
SEM	MLLN	GEO: GSE74812
	AF4	
	H3K4me3	
	H3K4me1	
	H3K27ac	
	H3K79me2	
	CFP1	
	ENL	
	ATAC-seq	
	(MLL-AF4 gene list)	GEO: GSE13313
MV4;11	MLLN	GEO: GSE73528
	MLLC	
	AF4C	
Human CD34+	FLAG (MLL-Af4)	GEO: GSE84116
mES	Streptavidin (MLL-AF9)	GEO: GSE29130
	ER (MLL-ENL)	ArrayExpress: E-MTAB-3593

Patient data

Clinical Trial	Patient Type	Accession Number
ECOG E2993	Various ALL subtypes	GEO: GSE34861
COG P9906	Various ALL subtypes	GEO: GSE28460
St Jude's Research Hospital	Various pediatric ALL subtypes	http://www.stjuderesearch.org/site/data/ALL3/

Supplemental Experimental Procedures

Cell lines and culture
SEM, (Greil et al., 1994) ML-2 (Ohyashiki et al., 1986), RCH-ACV (Jack et al., 1986) and KOPN-8 (Matsuo and Drexler, 1998) were purchased from DSMZ (www.cell-lines.de) and cultured in IMDM or RPMI 1640 supplemented with 10% FCS. THP1, MV4-11 and CCRF-CEM cells were purchased from ATCC (www.lgcstandards-atcc.org) and cultured in RPMI 1640 supplemented with 10% FCS. SEM-K2 were kindly provided by Dr. Carolyn Felix (University of Pennsylvania, Philadelphia, PA). SEM-K2 cells are a subclone of SEM cells with identical features (Zweidler-McKay et al., 2005). HEK293T cells were cultured in DMEM supplemented with 10% FCS. Mouse ES cells with a TetO-array (TOT2N mESC) were kindly provided by Dr. Rob Klose (University of Oxford) and were grown in DMEM supplemented by with 10% FCS, LIF and β-mercaptoethanol. TOT2N mESCs were treated with doxycycline for 6 hours when appropriate for the ChIP studies. For the nascent RNA-seq studies, SEM cells were treated with the DOT1L inhibitor EPZ-5676 (Epizyme) for 7 days, with fresh inhibitor added on days 0, 3 and 6.

Drug Treatment Studies
SEMK2 cells were seeded at 40,000 cells /well in a 96 well plate. The cells were treated with a DMSO control, ABT-199 (320, 160, 80, 40, 20, 10, 5 nM and DMSO control) alone, or in combination with a 1:10 ratio of either EPZ5676, SGC0946 or MI503 (3200, 1600, 800, 400, 200, 100, 50 nM and DMSO control). Media was changed once at 72 hr, cells were split and kept at about 40,000 cells/well, CellTiter-Glo® Luminescent Cell Viability Assay was run at 7days. Calcusyn 2.0 software (Biosoft, Great Shelford, UK) was used to calculate IC_{50} values and combination index.

Human progenitor cell isolation
Samples: Mononuclear cells (MNC) from term cord blood (CB) and second trimester human fetal bone marrow (FBM) were used for the experiments. CB was collected under the auspices of a National Research Ethics Service-approved study with written informed consent. Human fetal bone samples were obtained through the Human Developmental Biology Resource (www.hdbr.org).
Sample preparation: FBM was obtained by repeated flushing of the fetal long bones with DMEM (Gibco). CB and FBM samples were red cell and granulocyte depleted by density gradient separation with Ficoll-Paque Premium (GE Healthcare) to isolate MNC. MNC were either fixed fresh or following thawing after cryopreservation.

Patient-derived MLL-AF4 primograft cells
L826 patient cells were obtained from Newcastle Haematological BioBank and as such are covered by a generic approval given by the Newcastle & North Tyneside Ethics Committee (REC reference number: 07/H0906/109+5). The mouse transplantation experiments are covered by a Home Office Project licence PPL 60/4552. The L826 cells were originally derived from a diagnostic ALL patient sample, which have been passed through an mouse prior to ChIP-seq analysis. To that end, L826 patient cells were engrafted in NSG mice and the primograft was harvested from a mouse spleen. Cells were thawed and maintained in RPMI with 20% serum before they were fixed for ChIP-seq experiments.

siRNA experiments
Briefly, using a rectangle pulse EPI 2500 electroporator (Fischer, Heidelberg), 7 X 10^7 SEM cells were subjected to a 10msec 350V (SEM) electroporation in the presence of 300 pmol siRNA. MLLAF4 siRNA sequences were obtained from (Thomas et al., 2005) and are the following: siMA6 (sense, AAGAAAAGCAGACCUACUCCA; antisense, UGGAGUAGGUCUGCUUUUCUUUU), targeting the MLL exon 9 and AF4 exon 4 MLL-AF4 fusion site present in SEM cells. As control siRNAs we used the mismatch control siMM (sense,AAAAGCUGACCUUCUCCAAUG; antisense, CAUUGGAGAAGGUCAGCUUUUCU). For Menin and PAF1 siRNA experiments in SEM cells, we used Ambion Silencer Select Negative Control #2 (catalog AM4613), PAF1#1 (4392420, s29267), PAF1#2 (4392420, s29269) and Menin (4392420, s8682). Electroporation conditions were the same as for MLL-AF4 siRNAs. For testing MLL-AF4 recruitment by Menin and PAF1, MLL-AF4 cDNA was transfected into TetR-Menin and TetR-PAF1 mES cells at 60-70% confluency, either alone or with mouse Ledgf (Psip1) siRNA (Dharmacon SMARTpool, L-056571-01-0005), using Lipofectamine 2000. Cells were collected 24 hours after transfection.

Antibodies used for western blot analysis
αMenin (Bethyl, A300-105A), αGAPDH (Bethyl, A300-641A), αFlag (Sigma, F1804), αPAF1 (Bethyl, A300-172A), αCDK6 (Cell Signaling, 3136), αBCL11A (Bethyl, A300-382A), αBCL2 (Cell Signaling, 2870),

αRUNX1 (Cell Signaling, 4334), αMEF2C (Cell Signaling, 5030), αH3K79me3 (Diagenode, C15410068), αH4 (Abcam, ab7311)

Chromatin immunoprecipitation assays
For ChIP and ChIP-seq, fixed samples of up to 10^8 cells were sonicated on a Covaris (Woburn, MA) according to the manufacturers' recommendations. Ab:chromatin complexes were collected with a mixture of Protein A and Protein G Dynabeads (Life Technologies, Grand Island, NY) by using a magnet and were then washed three times with a solution of 50mM Hepes-KOH, pH 7.6, 500mM LiCl, 1mM EDTA, 1% NP-40, and 0.7% Na-deoxycholate. After a Tris-EDTA wash, samples were eluted, treated with RNAse and proteinase K, and purified by using a Qiagen PCR purification kit. ChIP samples were quantified relative to inputs (Milne et al., 2009). Briefly, the amount of genomic DNA co-precipitated with antibody was calculated as a percentage of total input using the following formula: $\Delta CT = CT$ (input) – CT (ChIP), total percentage = $2^{\Delta CT} \times 5.0\%$. A 50-µL aliquot taken from each of 1 mL of sonicated, diluted chromatin before Ab incubation served as the input, and thus the signal from the input samples represents 5% of the total chromatin used in each ChIP. CT values were determined by choosing threshold values in the linear range of each PCR reaction. TetR fusion proteins were detected in ChIP using an FS2 antibody.

ChIP sequencing
ChIP samples were submitted to the Wellcome Trust Centre for Human Genetics for library preparation (Lamble et al., 2013) and sequencing. Samples were sequenced using a HiSeq 2000, a HiSeq 2500 and 50bp paired-end sequencing. Data were mapped to the *Homo sapiens* hg18 or *Mus musculus* mm10 genome using Bowtie. Conversion to bam files was done using samtools. Duplicate reads were removed and data was normalized to an input track, in SeqMonk. Peaks were called using the probe generator in SeqMonk and filtered to retain only those that showed a >3-fold enrichment over the input track. MLL-AF4 peaks were defined as any MLL(N) peaks that overlapped with at least one AF4(C) peak. Wild-type MLL peaks in SEM cells were defined as any MLL(N) peaks that did not overlap with an AF4(C) peak. Broad H3K4me3 peaks were defined as the top 5% widest peaks (kb) of H3K4me3, as in the original study (Benayoun et al., 2014). Super enhancers were defined using the HOMER (http://homer.salk.edu/homer/index.html) *findPeaks –style super* command, based on the algorithm of the original study (Whyte et al., 2013), with a combined BRD4, MED1, H3K27ac and H3K4me1 dataset. Data for heat maps and meta gene plots were generated using the HOMER *annotatePeaks* command. Heat maps of spreading peaks in Figure 5 were generated by using the Samtools *view –c* command to get the reads under the peaks in 2.5kb bins. The R package *ggplot2* was used to generate the heat map.

Antibodies used for ChIP and ChIP-seq assays
αH3K36me3 (Diagenode, pAb-192-050), αH3K27ac (Diagenode, C15410196), αH3K4me3 (Diagenode, pAb-003-050), αH3K4me1 (Diagnode, pAb-194-050), αH3K79me2 (Active Motif, 39143), αH3K79me3 (Diagenode, pAb-068-050), αMLL1 (Bethyl, A300-086A), αAF4 (abcam, 31812), αKDM2B (gift from Dr Rob Klose (University of Oxford)), αBRD4 (Bethyl, A301-985A), αMED1 (Bethyl, A300-793A), αMenin (Bethyl, A300-105A), αPAF1 (Bethyl, A300-172A), αLEO1 (A300-175A), αCFP1 (Bethyl, A303-161A), αENL (A302-268A), αFS2 (gift from Dr Rob Klose).

Bio-CAP
Bio-CAP was performed as described previously (Blackledge et al., 2012). Briefly, 50µL biotinylated KDM2B CXXC domain (0.5µg/µL, a generous gift from Dr Rob Klose) was diluted with 425µL BC150 and added to 25µL BC150-rinsed magnetic neutravidin beads (Sera-Mag SpeedBeads NeutraAvidin Microparticles, Thermo Scientific). As a control, 25µL beads were also added to 475µL of BC150 alone. Tubes were rotated end-over-end at 4˚C for 1 hour. Beads were washed 3 times with 1M CAP Buffer (1000mM NaCl; 20mM HEPES pH7.9; 0.1% Triton X-100; 12.5% glycerol), using a magnet, and then 100mM CAP Buffer. 100µL DNA (100ng/µL) was diluted with 900µL BC150 (10µL DNA (10%) was diluted with 90µL BC150 for input). 500µL of DNA in BC150 was added to KDM2B-hybridised beads and the other 500µL was added to the beads-only control. Tubes were incubated end-over-end at 4˚C for 1 hour. After incubation, supernatant was removed from beads using a magnet – this was the flow-through material. Beads were washed twice with 100mM CAP Buffer. Material was then eluted from beads using increasing concentrations of salt: 300mM, 500mM, 700mM and 1M. This was done in 50µL CAP buffer at room temperature for 10 minutes, with gentle tapping, twice to give a total volume of 100µL for each elution. DNA was purified using a Qiagen PCR purification kit.

Nascent RNA-seq
10^8 Cells were treated with 500µM 4-thiouridine (4-SU) for 1 hour. Cells were lysed using trizol and RNA was precipitated with ethanol. 4-SU-incorporated RNA was biotinylated by labelling with 1mg/ml Biotin-HPDP for 90 minutes at room temperature. Following chloroform extraction, labelled RNA was separated using magnetic

streptavidin beads. Beads were washed using a magnetic µMACS stand before RNA was eluted in two rounds of elution with 100µL 100mM DTT. RNA was purified using a Qiagen RNeasy MinElute kit. Samples were sequenced using a NextSeq 500 and 40bp paired-end sequencing.

Nascent RNA-seq and Gene Expression Analysis
Following QC analysis with the fastQC package (http://www.bioinformatics.babraham.ac.uk/projects/fastqc), reads were aligned using STAR (Dobin et al., 2013) against the human genome assembly (NCBI build36 (hg18) UCSC transcripts). Reads that were identified as PCR duplicates using Samtools (Li et al., 2009) were discarded. For experiments that did not involve inhibitor or siRNA treatments, gene expression was measured by calculating the nascent RNA-seq reads over exons for each gene and normalizing to reads per sample and kb of exons and subsequently normalized to GAPDH expression. For inhibitor and siRNA experiments, gene expression levels were quantified as read counts using the featureCounts function (Liao et al., 2014) from the Subread package (Liao et al., 2014) with default parameters. The read counts were used for the identification of global differential gene expression between specified populations using the edgeR package (Robinson et al., 2010). RPKM values were also generated using the edgeR package. Genes were considered differentially expressed between populations if they had an adjusted *p*-value (FDR) of less than 0.05.

Transcriptional Variability
Transcriptional variability of a gene was measured as the standard deviation of expression across four biological replicates of nascent RNA-seq

PCR Primers for RT-PCR
The mouse Mll1 Taqman primer-probe set was as follows: Mll1 primer For: CTGAATGACCTCTCTGACTGTGAAGA, Mll1 probe: ACTCTTTCCTATTGGATACCAGTGTTCTCGGG, Mll1 primer Rev: GGCATCTGTGGTGCTCCAGTA. MLL-AF4 SYBR Green primers were as follows: MLL-AF4 For: AGGTCCAGAGCAGAGCAAAC, MLL-AF4 Rev: CGGCCATGAATGGGTCATTTC. Mouse GAPDH SYBR Green primers were as follows: GAPDH For: GTCTCCTGCGACTTCAGC, GAPDH Rev: TCATTGTCATACCAGGAAATGAGC. Mouse Ledgf For: CGATCAAGAGGGTGAAAAGAA, Ledgf Rev: TTGGCCTTTTAGCATGTTCC

PCR Primers for ChIP
Negative control For: GGCTCCTGTAACCAACCACTACC, Negative control Rev: CCTCTGGGCTGGCTTCATTC; HOXA9 For: ATGCTTGTGGTTCTCCTCCAGTTG, HOXA9 Rev: CCGCCGCTCTCATTCTCAGC; HOXC8 For: AGACTTCTTCCACCACGGCAC; HOXC8 Rev: TAAGCGAGCACGGGTTCTGC; BCL11A CGI1 For: ACACCCAGTGCCCAGAATTG, BCL11A CGI1 Rev: CGCGGGTCCTGAGATTCATT; BCL11A CGI2 For: AGGCTGAGTGTTGTGGAGAC, BCL11A CGI2 Rev: CGTTGGAAGCTGCCTTTGTT; BCL11A CGI3 For: GCCAGGCTAACATAGACCTCC, BCL11A CGI3 Rev: GAGGCCAACTCTTCCACTCC; ARID1B CGI2 For: AAGTGCCGTGACCTTCACAT, ARIB1B CGI2 Rev: CCTTGCATTACCTCTGCCCA; CPEB2 CGI For: ATTGCTGGAGAAGGTGCGTG, CPEB2 CGI Rev: TCCCTCATTGGACAGCGAGA; CPEB2 non-CGI For: CATATGGGCCATCCTATTCTCTG, CPEB2 non-CGI Rev: GCTCTGGTTCTAACTCCTAGAAA; BCL6 For: ACAGAGGCTCAAAGGAAAACAA; BCL6 Rev: GCCTTAACTCCACAAGTTTGA; ARID2 For: GGGACTTGCGATTGGTTATTG; ARID2 Rev: CACCAGAGAGCCCTGTATTT; TET2 For: CCTCAAGGCAGCAACTAAGAAC; TET2 Rev: GAGTCACTCCCAGAGGTCCT; GNAQ For: GTCCATCATGGCGTGCT; GNAQ Rev: CGGACGGTACTCACCGA; MBNL1 For: CAGTCCTTTCACTGCATGTTT; MBNL1 Rev: CAGACAGACACTTTGCCATTAC; SENP6 For: GAGAAGGGAGGGTATACTGGAA; SENP6 Rev: TCGCTCATCCCTCGTACTT; ETV6 For: TTCCTGGTGGCTCCTTTAAGAG; ETV6 Rev: ACTGACGTGAATTCCCAGCA; MYC For: GAGCAGCAGAGAAAGGGAGA; MYC Rev: CAGCCGAGCACTCTAGCTCT; SPT3 uCpG For: CATGTTTTACAGTGCCTGGTACG; SPT3 uCpG Rev: AGACTGCCCTCCTACGCTA; SPT3 non-uCpG For: GCTGAACCCAAATTTATATTGCCC; SPT3 non-uCpG Rev: AGCTCTGAGGGTTACTGACCA

Patient Datasets and gene expression microarray data
Microarray gene expression data from three large cohorts of patients with ALL were analyzed. These cohorts included the Eastern Cooperative Oncology Group (ECOG) Clinical Trial E2993 (GEO#: GSE34861) cohort: 191 total samples comprising 78 BCR-ABL1 patients, 6 E2A-PBX1 patients, 25 MLLr patients (t(4;11): 17, other MLLr: 8), and 82 other B-ALL patients; (Geng et al., 2012) the Children's Oncology Group (COG) Clinical Trial P9906 (GEO#: GSE28460) cohort: 207 total samples, 23 E2A-PBX1 patients, 21 MLLr patients,

3 RUNX1-ETV6 patients, 155 other B-ALL patients (trisomy 4 or 10 patients); (Harvey et al., 2010) and the St. Jude Research Hospital pediatric ALL clinical trial cohort: 132 total samples, 15 BCR-ABL1 patients, 18 E2A-PBX1 patients, 20 MLLr patients, 20 RUNX1-ETV6 patients, 17 hyperdiploid patients, 28 other B-ALL patients, 14 T-ALL patients (Ross et al., 2003). This last cohort has no GEO number, but raw data can be downloaded from the following site: http://www.stjuderesearch.org/site/data/ALL3/. The microarray data was normalized with RMA method (Bolstad et al., 2003) using Expression ConsoleTM software (Version 1.1, Affymetrix, Santa Clara, CA) for the Affymetrix arrays HG-U133 plus2 (COG data, n=207) or NimbleScan software (version 2.5, Roche NimbleGen, Madison, WI) for the NimbleGen arrays HG18 60mer expression 385K platform (ECOG data, n=191). The patients in each clinical trial were grouped into subtypes according to their cytogenetic features: BCR-ABL, TCF3-PBX1, MLLr (MLL rearranged), TEL-AML1, or other ALLs which are negative to the above translocations. The downstream microarray analysis was performed using R version 2.14.0 (R Development Core Team. R: A Language and Environment for Statistical Computing. 2009, http://www.R-project.org). The heatmap was generated with Cluster Cluster/TreeView3.0 software (http://bonsai.hgc.jp/~mdehoon/software/cluster/software.htm).

Survival analysis
In order to test if the "spreading" MLL-AF4 targets (n=117) consist of a prognostic biomarker model predictive of ALL survival we used the **s**upervised **p**rincipal **c**omponents (superPC) algorithm developed by Bair and Tibshirani (Bair and Tibshirani, 2004) in two ALL clinical datasets: COG P9906 (n=207) and ECOG E2993 (n=165). Univariate Cox scores, which measure the correlation between gene expression levels and overall survival (OS) or relapse free survival (RFS) of the ALL patients, were computed for each gene in the gene lists (such as a total of N genes). Only genes with absolute Cox scores of greater than a specific threshold were retained for subsequent prediction (such as M genes, $M<=N$). This threshold was determined using a 10-fold cross-validation procedure that evaluated the prognostic significance of different thresholds within the dataset. A principal component analysis (PCA) was performed using the expression levels of the M genes as features. Then the first three principal components were used in a regression model to compute a prognostic score for each patient. According to these predicted prognostic scores, the patients were divided into two groups: 50% high-risk (> the median score) and 50% low-risk (<= the median score). Not necessarily all the genes get used in the list, this analysis just allows us to conclude that there is a signature within the set that is predictive of a poor prognosis. The procedure was applied in both ALL cohorts. The Kaplan-Meier method was used to estimate overall survival (OS) and relapse-free survival (RFS). Log-rank test was used to compare survival differences between patient groups with high or low risk. The R packages 'Superpc' (Bair and Tibshirani, 2004) and 'survival' version 2.35-8 (Therneau, 2015) was used for the survival analysis.

Supplemental References

Bolstad, B.M., Irizarry, R.A., Astrand, M., and Speed, T.P. (2003). A comparison of normalization methods for high density oligonucleotide array data based on variance and bias. Bioinformatics *19*, 185-193.

Dobin, A., Davis, C.A., Schlesinger, F., Drenkow, J., Zaleski, C., Jha, S., Batut, P., Chaisson, M., and Gingeras, T.R. (2013). STAR: ultrafast universal RNA-seq aligner. Bioinformatics *29*, 15-21.

Greil, J., Gramatzki, M., Burger, R., Marschalek, R., Peltner, M., Trautmann, U., Hansen-Hagge, T.E., Bartram, C.R., Fey, G.H., Stehr, K*., et al.* (1994). The acute lymphoblastic leukaemia cell line SEM with t(4;11) chromosomal rearrangement is biphenotypic and responsive to interleukin-7. Br J Haematol *86*, 275-283.

Jack, I., Seshadri, R., Garson, M., Michael, P., Callen, D., Zola, H., and Morley, A. (1986). RCH-ACV: a lymphoblastic leukemia cell line with chromosome translocation 1;19 and trisomy 8. Cancer Genet Cytogenet 19, 261-269.

Lamble, S., Batty, E., Attar, M., Buck, D., Bowden, R., Lunter, G., Crook, D., El-Fahmawi, B., and Piazza, P. (2013). Improved workflows for high throughput library preparation using the transposome-based Nextera system. BMC Biotechnol *13*, 104.

Li, H., Handsaker, B., Wysoker, A., Fennell, T., Ruan, J., Homer, N., Marth, G., Abecasis, G., Durbin, R., and Genome Project Data Processing, S. (2009). The Sequence Alignment/Map format and SAMtools. Bioinformatics *25*, 2078-2079.

Liao, Y., Smyth, G.K., and Shi, W. (2014). featureCounts: an efficient general purpose program for assigning sequence reads to genomic features. Bioinformatics *30*, 923-930.

Matsuo, Y., and Drexler, H.G. (1998). Establishment and characterization of human B cell precursor-leukemia cell lines. Leuk Res *22*, 567-579.

Milne, T.A., Zhao, K., and Hess, J.L. (2009). Chromatin immunoprecipitation (ChIP) for analysis of histone modifications and chromatin-associated proteins. Methods Mol Biol 538, 409-423.

Ohyashiki, K., Ohyashiki, J.H., and Sandberg, A.A. (1986). Cytogenetic characterization of putative human myeloblastic leukemia cell lines (ML-1, -2, and -3): origin of the cells. Cancer Res *46*, 3642-3647.

Robinson, M.D., McCarthy, D.J., and Smyth, G.K. (2010). edgeR: a Bioconductor package for differential expression analysis of digital gene expression data. Bioinformatics *26*, 139-140.

Ross, M.E., Zhou, X., Song, G., Shurtleff, S.A., Girtman, K., Williams, W.K., Liu, H.C., Mahfouz, R., Raimondi, S.C., Lenny, N*., et al.* (2003). Classification of pediatric acute lymphoblastic leukemia by gene expression profiling. Blood *102*, 2951-2959.

Shi, A., Murai, M.J., He, S., Lund, G., Hartley, T., Purohit, T., Reddy, G., Chruszcz, M., Grembecka, J., and Cierpicki, T. (2012). Structural insights into inhibition of the bivalent menin-MLL interaction by small molecules in leukemia. Blood *120*, 4461-4469.

Therneau, T.M. (2015). A Package for Survival Analysis in S. version 2.35-8. http://cranr-projectorg/package=survival.

Thomas, M., Gessner, A., Vornlocher, H.P., Hadwiger, P., Greil, J., and Heidenreich, O. (2005). Targeting MLL-AF4 with short interfering RNAs inhibits clonogenicity and engraftment of t(4;11)-positive human leukemic cells. Blood *106*, 3559-3566.

Zweidler-McKay, P.A., He, Y., Xu, L., Rodriguez, C.G., Karnell, F.G., Carpenter, A.C., Aster, J.C., Allman, D., and Pear, W.S. (2005). Notch signaling is a potent inducer of growth arrest and apoptosis in a wide range of B-cell malignancies. Blood *106*, 3898-3906.
