# Supplementary material for: MLL-AF4 Spreading Identifies Binding Sites that Are Distinct from Super-Enhancers and that Govern Sensitivity to DOT1L Inhibition in Leukemia
Source: Cell Rep. 2017 Jan 10;18(2):482–95. doi: 10.1016/j.celrep.2016.12.054 (PMC5263239; doi:10.1016/j.celrep.2016.12.054)
Supplement: Document S2. Article plus Supplemental Information [file mmc4.pdf]

Cell Reports

Article

MLL-AF4 Spreading Identifies Binding Sites that Are Distinct from Super-Enhancers and that Govern Sensitivity to DOT1L Inhibition in Leukemia

Graphical Abstract

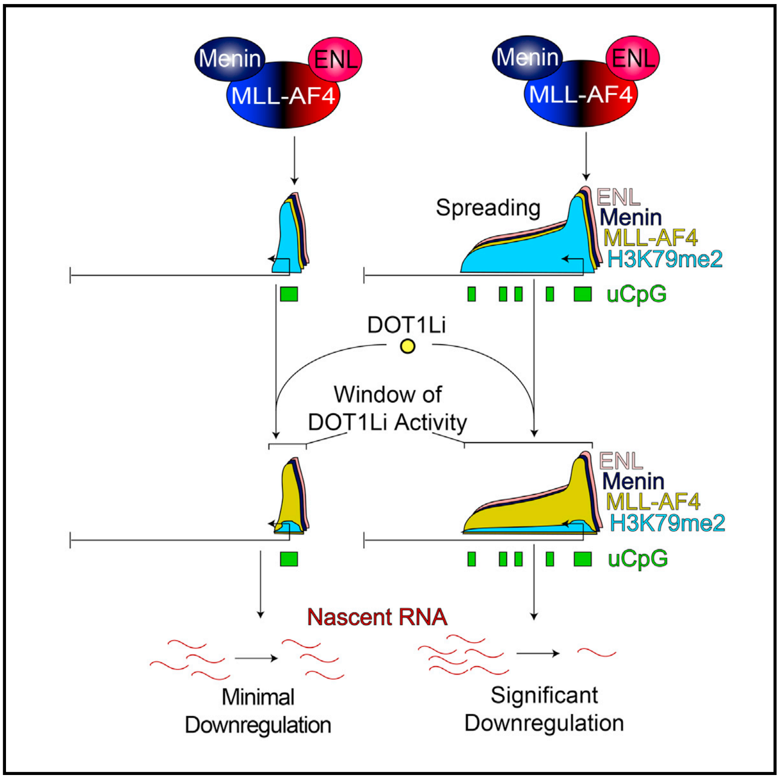

Highlights

- MLL-AF4 binding requires an unmethylated CpG (uCpG) island and Menin
- MLL-AF4 and Menin can spread into the gene body of some targets
- Spreading targets are highly transcribed and have an aberrant chromatin signature
- Spreading of MLL-AF4 is a predictor of sensitivity to DOT1L inhibitors

Authors

Jon Kerry, Laura Godfrey, Emmanouela Repapi, ..., Robert J. Klose, Huimin Geng, Thomas A. Milne

Correspondence

thomas.milne@imm.ox.ac.uk

In Brief

Translocations of the MLL gene produce fusion proteins such as MLL-AF4 that cause poor-prognosis leukemias. Kerry et al. show that MLL-AF4 can spread into the gene body of some target genes. Spreading targets have an aberrant chromatin signature and are sensitive to DOT1L inhibitors.

Accession Numbers

GSE83671

Kerry et al., 2017, Cell Reports *18*, 482–495
January 10, 2017 © 2017 The Author(s).
http://dx.doi.org/10.1016/j.celrep.2016.12.054

CellPress

OPEN ACCESS
CellPress

Cell Reports
Article

MLL-AF4 Spreading Identifies Binding Sites that Are Distinct from Super-Enhancers and that Govern Sensitivity to DOT1L Inhibition in Leukemia

Jon Kerry,[1] Laura Godfrey,[1] Emmanouela Repapi,[2] Marta Tapia,[1] Neil P. Blackledge,[3] Helen Ma,[4] Erica Ballabio,[1] Sorcha O'Byrne,[5] Frida Ponthan,[6] Olaf Heidenreich,[6] Anindita Roy,[5] Irene Roberts,[1,5] Marina Konopleva,[4] Robert J. Klose,[3] Huimin Geng,[7] and Thomas A. Milne[1,8,*]
[1]MRC, Molecular Haematology Unit, NIHR Oxford Biomedical Research Centre Programme
[2]Computational Biology Research Group, Weatherall Institute of Molecular Medicine
University of Oxford, John Radcliffe Hospital, Oxford OX3 9DS, UK
[3]Laboratory of Chromatin Biology and Transcription, Department of Biochemistry, University of Oxford, Oxford OX1 3QU, UK
[4]Department of Leukemia, The University of Texas MD Anderson Cancer Center, Houston, TX 77030, USA
[5]Department of Paediatrics, University of Oxford, Children's Hospital, John Radcliffe, Oxford OX3 9DU, UK
[6]Wolfson Childhood Cancer Research Centre, Northern Institute for Cancer Research, Newcastle University, Newcastle Upon Tyne NE1 7RU, UK
[7]Department of Laboratory Medicine, University of California, San Francisco, San Francisco, CA 94143, USA
[8]Lead Contact
*Correspondence: thomas.milne@imm.ox.ac.uk
http://dx.doi.org/10.1016/j.celrep.2016.12.054

SUMMARY

Understanding the underlying molecular mechanisms of defined cancers is crucial for effective personalized therapies. Translocations of the mixed-lineage leukemia (*MLL*) gene produce fusion proteins such as MLL-AF4 that disrupt epigenetic pathways and cause poor-prognosis leukemias. Here, we find that at a subset of gene targets, MLL-AF4 binding spreads into the gene body and is associated with the spreading of Menin binding, increased transcription, increased H3K79 methylation (H3K79me2/3), a disruption of normal H3K36me3 patterns, and unmethylated CpG regions in the gene body. Compared to other H3K79me2/3 marked genes, MLL-AF4 spreading gene expression is downregulated by inhibitors of the H3K79 methyltransferase DOT1L. This sensitivity mediates synergistic interactions with additional targeted drug treatments. Therefore, epigenetic spreading and enhanced susceptibility to epidrugs provides a potential marker for better understanding combination therapies in humans.

INTRODUCTION

Translocations of the mixed-lineage leukemia (*MLL*) gene produce over 120 different MLL fusion proteins (MLL-FPs) that cause aggressive acute leukemias, the most common one being the *MLL-AF4* fusion (Ballabio and Milne, 2012; Meyer et al., 2013). Despite much progress in the treatment of childhood leukemias, infants carrying *MLL* rearrangements have a very poor prognosis (Pui et al., 2011); thus, improving therapies for MLL-FP patients remains an unmet need. Because MLL-FPs are considered to be the main drivers of leukemogenesis, their function regulating downstream target genes is key to understanding MLL-rearranged (MLLr) leukemias and for designing targeted therapies.

MLL-FPs retain several domains (Figure 1A) including a CXXC domain that binds specifically to unmethylated CpG (uCpG) DNA (Birke et al., 2002), interaction sites with the multiple endocrine neoplasia type 1 (Menin) (Yokoyama et al., 2005) and lens epithelium-derived growth factor (LEDGF) proteins (Yokoyama and Cleary, 2008), and an interaction with the polymerase-associated factor protein complex (PAFc) (Milne et al., 2010; Muntean et al., 2010). Recruitment of MLL-FPs to gene targets is thought to be controlled by Menin, LEDGF, and PAFc interactions as well as CXXC binding to uCpGs (Milne et al., 2010; Muntean et al., 2010; Okuda et al., 2014; Yokoyama and Cleary, 2008; Yokoyama et al., 2005). Supporting this, a minimal MLL-FP containing just the PWWP domain of LEDGF, the CXXC domain of MLL, and the transactivation domain of the fusion partner can transform bone marrow progenitors and recapitulate MLL-FP binding at a few select genes (Okuda et al., 2014). However, a minimal CXXC domain can be recruited to the *HoxA9* locus in the absence of a Menin/LEDGF interaction (Milne et al., 2010), although others have suggested that the CXXC domain has no role in recruitment and instead protects uCpG sites from methylation (Risner et al., 2013). Recent data also suggest that Menin is unimportant for wild-type MLL (Borkin et al., 2015; Li et al., 2013), whereas LEDGF is required for MLL but not MLL-FP recruitment (Zhu et al., 2016). Thus, it still remains an open question exactly how MLL-FPs are recruited to particular gene targets.

MLL-FP recruitment is associated with increased histone 3 lysine 79 di- and tri-methylation (H3K79Me2/3) at target genes, an epigenetic mark associated with gene activation (Bernt

 © 2017 The Author(s).
This is an open access article under the CC BY license (http://creativecommons.org/licenses/by/4.0/).

OPEN ACCESS
CellPress

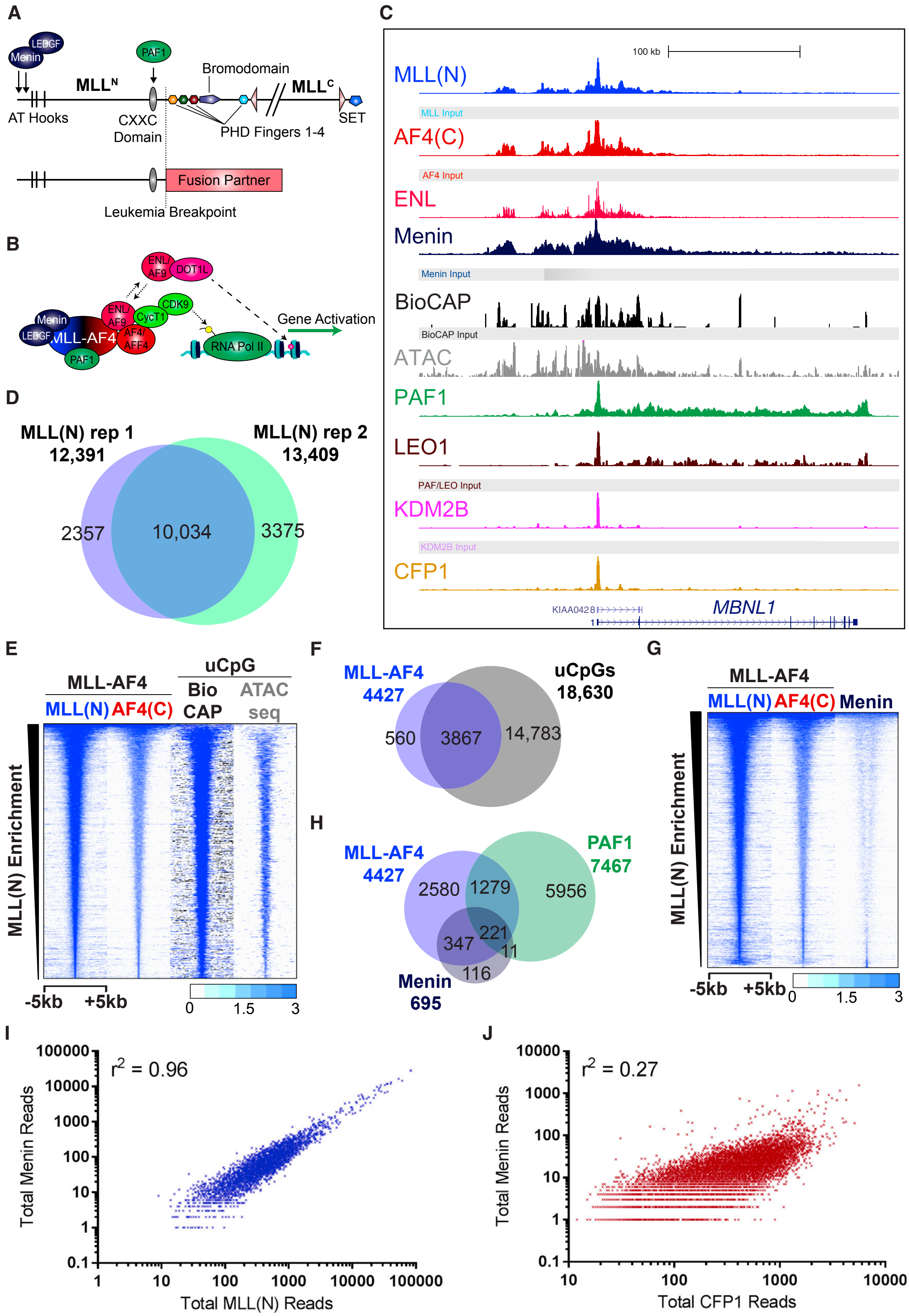

(legend on next page)

et al., 2011; Guenther et al., 2008; Krivtsov et al., 2008; Milne et al., 2005). H3K79Me2/3 levels are controlled by the disruptor of telomeric silencing 1-like (DOT1L) protein (Jones et al., 2008). In MLL-FP leukemias, DOT1L directly interacts with AF9 or ENL (Biswas et al., 2011; Mueller et al., 2007), and can be mis-targeted to MLL-FP-bound genes where it is associated with inappropriate activation of gene expression (Milne et al., 2005) (Figure 1B). A recent study analyzing MLL-ENL binding suggests that there are two distinct classes of binding: proximal (5′) or distal (3′) to the transcription start site, with proximal binding being particularly sensitive to DOT1L inhibition (Garcia-Cuellar et al., 2016). MLL-AF4 can also bind in broad regions of up to 100 kb that correlate with large domains of H3K4me3 (Guenther et al., 2008) and MLL-AF9 transformed mouse bone marrow cells display H3K79me2 peaks with a similar wide spatial distribution (Bernt et al., 2011). Despite all this work, there is no current consensus on whether the main activity of MLL-FPs is the recruitment of DOT1L or whether different binding patterns of MLL-FPs are associated with distinct functional outcomes.

Here, we reveal a strong co-dependent relationship between MLL-AF4 and Menin binding at a small number of target genes containing uCpGs. At a subset of these gene targets, we observe MLL-AF4 and Menin spreading that is bookended by uCpGs. These spreading targets are distinct from super-enhancers, are associated with high levels of gene transcription, have an aberrant H3K79me2/H3K36me3 signature, and are predictive of a poor overall survival in patients with acute lymphoblastic leukemia (ALL). These gene targets also display a remarkable dependence on H3K79me2 and the fusion protein for their sustained expression in leukemia. Together, this work shows that MLL-FP spreading occurs at genes important in MLL leukemogenesis and has the potential to act as a biomarker for therapeutic response.

RESULTS

MLL-AF4 Binds Exclusively to a Subset of uCpGs

Using MLL(N) and AF4(C) chromatin immunoprecipitation sequencing (ChIP-seq) in the human MLL-AF4 SEM cell line (Figure 1C), we identified 4,427 peaks and a gene set of 2,597 unique genes (Table S1). MLL(N) ChIP-seq replicates had 81% peaks in common (Figure 1D), which identified 96.4% promoter-bound MLL(N) gene targets from our original ChIP-seq dataset (Table S1, “Overlaps”). This gave us high confidence in the reproducibility of our gene target identification. To test the specificity of the MLL-AF4 target set, we performed MLL-AF4 small interfering RNA (siRNA) knockdowns coupled with nascent RNA sequencing (RNA-seq) (Figure S1A) and MLL-AF4 ChIP-seq (Figure S1B). Our MLL-AF4 gene target set was significantly down-regulated at most genes (Figure S1A, $p < 0.01$, Mann-Whitney U test) and lost MLL-AF4 ChIP-seq signal at 85% of target gene promoters (Figures S1B and S1C). Among the 15% of MLL-AF4 gene targets with no reduced ChIP-seq signal, one-third showed a significant change in gene expression following MLL-AF4 knockdown (Figure S1C). This suggests that these targets are also directly regulated by MLL-AF4, even though they consist primarily of promoters with a low MLL(N) ChIP-seq signal (Figure S1D).

If the CXXC domain is essential for MLL-AF4 recruitment, we would expect all MLL-AF4 binding sites to occur at regions of uCpGs. To test this, we used a biotinylated CXXC affinity purification (Bio-CAP) assay (Blackledge et al., 2012) for high-sensitivity detection of regions of uCpG dinucleotides in SEM cells, combined with an assay for transposase-accessible chromatin sequencing (ATAC-seq) (Buenrostro et al., 2013) to identify regions of open chromatin (Figure 1C). Similar to results using non-methylated CpG/methylated-CpG island recovery assay sequencing (CIRA/MIRA-seq) with MLL-AF6 (Okuda et al., 2014), all MLL-AF4 binding occurred at open uCpG regions (Figure 1E) with the highest uCpG enrichment occurring at the center of MLL-AF4 binding sites (Figure S1E). However, MLL-AF4 binding occurred at only 20% of uCpG sites (Figure 1F), indicating that open uCpG sites alone are not sufficient for MLL-AF4 recruitment. The Venn diagram (Figure 1F) shows a few MLL-AF4 sites that do not overlap with uCpGs, but it is clear from the heatmap that all MLL-AF4 binding sites occur at uCpG sites. The discrepancy is likely due to the reduced sensitivity of peak-calling programs used for the Venn diagram analysis.

To determine whether other CXXC domain-containing proteins (“CXXC proteins” from now on) are also restricted to only a proportion of uCpG sites, we performed ChIP-seq for CFP1 (a CXXC protein member of the SET1 complex associated with gene activation), and KDM2B (a CXXC protein involved in the recruitment of the polycomb group repressive complex [PRC] [Farcas et al., 2012]), in SEM cells (Figure 1C). In contrast to MLL-AF4, CFP1 and KDM2B bound more ubiquitously to uCpG sites, being found at 50% and 89% of all uCpG sites, respectively (Figure S1F). Because the CXXC domains of MLL-AF4, CFP1, and KDM2B are highly related (Long et al.,

Figure 1. MLL-AF4 Is Recruited Exclusively to uCpG Regions Bound by Menin

(A) Schematic showing MLL and MLL fusion protein interaction sites.

(B) Schematic showing the MLL-AF4 core complex.

(C) Example ChIP-seq, Bio-CAP-seq, and ATAC-seq tracks in SEM cells.

(D) Venn diagram showing overlap between two biological replicates of MLL(N) ChIP-seq.

(E) Heatmap showing ChIP-seq, Bio-CAP-seq, and ATAC-seq reads at all 4,427 MLL-AF4 binding sites in SEM cells. Scale bar represents tags per base pair (bp) per 10^7 reads.

(F) Venn diagram showing overlap between MLL-AF4 binding sites and uCpG regions (Bio-CAP-seq and ATAC-seq) in SEM cells.

(G) Heatmap showing MLL(N), AF4(C), and Menin ChIP-seq reads at all MLL-AF4 binding sites in SEM cells. Scale bar as in (E).

(H) Venn diagram showing overlap between MLL-AF4, PAF1, and Menin binding sites in SEM cells.

(I and J) Scatterplot showing a strong correlation ($r^2 = 0.96$) between MLL(N) and Menin ChIP-seq signal at all MLL-AF4 peaks (I) in SEM cells and a weak correlation between Menin and CFP1 ($r^2 = 0.27$) at all CFP1 peaks (J) in SEM cells.

See also Figure S1.

2013), the differences in the number of bound uCpGs may be due to other protein interactions influencing recruitment.

Genome-wide Recruitment of Menin Mirrors that of MLL-AF4

To investigate whether MLL-AF4-specific interactions contribute to uCpG binding, we analyzed two complexes thought to be involved in MLL-FP recruitment: Menin/LEDGF and PAFc (Figures 1A–1C). Except for a very few Menin binding sites (Figure 1G, very bottom of heatmap), we found that almost all MLL-AF4 binding sites overlap with detectable Menin binding (Figure 1G). Many MLL-AF4 binding sites had only low levels of detectable Menin (Figure 1G), and thus strict peak-calling parameters produce an MLL-AF4/Menin overlap at only a subset of binding sites (Figure 1H). However, when MLL-AF4 binding sites were separated into either high or low Menin binding, we saw a direct relationship between levels of Menin binding and levels of MLL-AF4 binding (Figure S1G). Furthermore, a direct comparison of MLL(N) and Menin ChIP-seq reads at MLL-AF4 binding sites showed a significantly strong positive correlation ($r^2 = 0.96$) (Figure 1I), whereas neither KDM2B nor CFP1 binding correlated with Menin (Figures 1J and S1H). Thus, an association with Menin represents a feature that may serve to restrict MLL-AF4 recruitment to a particular subset of uCpG sites.

ChIP-seq on two members of PAFc, PAF1 and LEO1 (Figure 1C), overlapped with less than one-half of MLL-AF4 binding sites, and 4,892 (78%) of PAFc binding sites had no MLL-AF4 binding (Figure S1I). Thus, compared to Menin binding, there is very little evidence for an MLL-AF4:PAF1 association genome-wide (Figure 1H), but it is possible that PAFc is necessary for recruitment only at select sites.

The Menin:MLL-AF4 Interaction Is Sufficient for Recruitment

To directly test the functionality of the interactions between Menin, MLL-AF4, and potentially PAFc, we used a Tet-repressor (TetR) system (see Figure 2A legend) previously designed to investigate the recruitment of PRC proteins (Blackledge et al., 2014). Using ChIP-qPCR, we detected binding of Menin but not PAF1 in the presence of TetR-MLL-AF4 but not the TetR-only control (Figure 2B, left versus right panel), and recruitment was lost upon treatment with doxycycline (Figure 2B, left panel, red line). Reciprocal experiments using TetR-Menin- and TetR-PAF1-expressing mESC lines transiently transfected with MLL-AF4 produced equivalent results (Figures S2A and S2B). Despite being able to recruit other members of PAFc, TetR-PAF1 was not sufficient to recruit MLL-AF4 (Figures S2B and S2C). Expression of different constructs was confirmed with either western blot or qPCR (Figures S2D and S2E). It has been recently shown that knockdowns of LEDGF sometimes lead to an increase in MLL-FP binding (Zhu et al., 2016). Similar to Zhu et al., we noticed a slight increase in MLL(N) ChIP at TetO in the presence of *Ledgf* siRNA (Figures S2F and S2G), although we were only able to achieve a 30% *Ledgf* knockdown at the RNA level (Figure S2F).

Our results so far support previous models suggesting that Menin recruits MLL-FPs (Yokoyama et al., 2005, 2010), and contrasts with previous reports that suggest that PAFc can recruit MLL-FPs (Milne et al., 2010; Muntean et al., 2010) (Figure 2C). The TetR assay does not establish directionality of these interactions; thus, it is also possible that MLL-FPs can recruit Menin (Caslini et al., 2007), or that the two proteins co-stabilize each other, as has recently been suggested for LEDGF and wild-type MLL (Zhu et al., 2016). In addition, it is possible that a relatively weak MLL-AF4:PAFc interaction is stabilized by other interactions when it occurs at active genes. To explore these issues further, we performed MLL-AF4, PAF1, or Menin siRNA knockdowns in SEM cells (Figures 2D–2H). MLL-AF4 knockdowns have a strong effect on the binding of Menin to gene targets (Figure 2Gi) and a moderate but detectable effect on PAF1 binding (Figure 2Hi). Menin knockdowns reduce both MLL-AF4 and PAF1 binding to gene targets (Figures 2E–2Hii), whereas two different PAF1 siRNAs produce a similar result in that they reduce Menin binding slightly but have little effect on MLL-AF4 except at the *HOXA9* locus (Figures 2E–2Hiii and S2H). Together, these data show that there is a complex co-recruitment relationship between MLL-AF4 and Menin, and that PAF1 does not have a major role in recruiting MLL-AF4 to most gene targets. However, MLL-AF4 either directly or indirectly, has a role in maintaining stable PAF1 binding at specific gene targets. To analyze MLL-AF4 function in further detail, we next tried to determine whether MLL-AF4 displayed distinct binding profiles at different subsets of genes.

Spreading of MLL-AF4 Marks a Subset of Highly Expressed Genes

An analysis of MLL-AF4 binding profiles revealed two patterns of binding. The majority of MLL-AF4 binding sites displayed narrow binding at the promoter and a normal pattern of H3K79me2 and H3K36me3 (Figure 3A). We also occasionally observed MLL-AF4 spreading greater than 4 kb into the gene body without exceeding the end of the gene, and this was associated with H3K79me2 spreading and a reduction or loss of H3K36me3 throughout the gene body (Figure 3B). Spreading was observed at 149 (3.4%) MLL-AF4 gene target isoforms (117 unique gene targets) in SEM cells (Figure S3A; Table S2). A Gene Ontology analysis revealed that spreading occurred at genes involved in hematopoiesis as well as lymphocyte activation and differentiation, showing that it could have a role in leukemia initiation or maintenance (Figure S3B). We confirmed that spreading was specific to MLL-AF4 using *MLL-AF4* siRNA knockdowns followed by ChIP-qPCR in regions of spreading at specific targets (Figure S3C). Spreading is reminiscent of broad MLL-AF4 binding domains at sites of broad H3K4me3 (Guenther et al., 2008), although we found that there was less than a 50% overlap between our spreading dataset and the MLL-AF4 target set originally identified by Guenther et al. (Figure S3D).

To test whether MLL-AF4 spreading was a marker of significant functional activity, we analyzed nascent RNA-seq data and found that spreading MLL-AF4 targets showed significantly higher expression compared to targets of non-spreading MLL-AF4, or active gene targets bound by CFP1 ($p < 0.0001$, two-tailed Mann-Whitney U test; Figure 3C). Spreading targets were also highly enriched for H3K79me2 at the 5′ end of the gene compared to non-spreading MLL-AF4 or non-MLL-AF4 targets (Figure 3D). The increased enrichment of H3K79me2 along with its spread into the gene body, strongly suggests

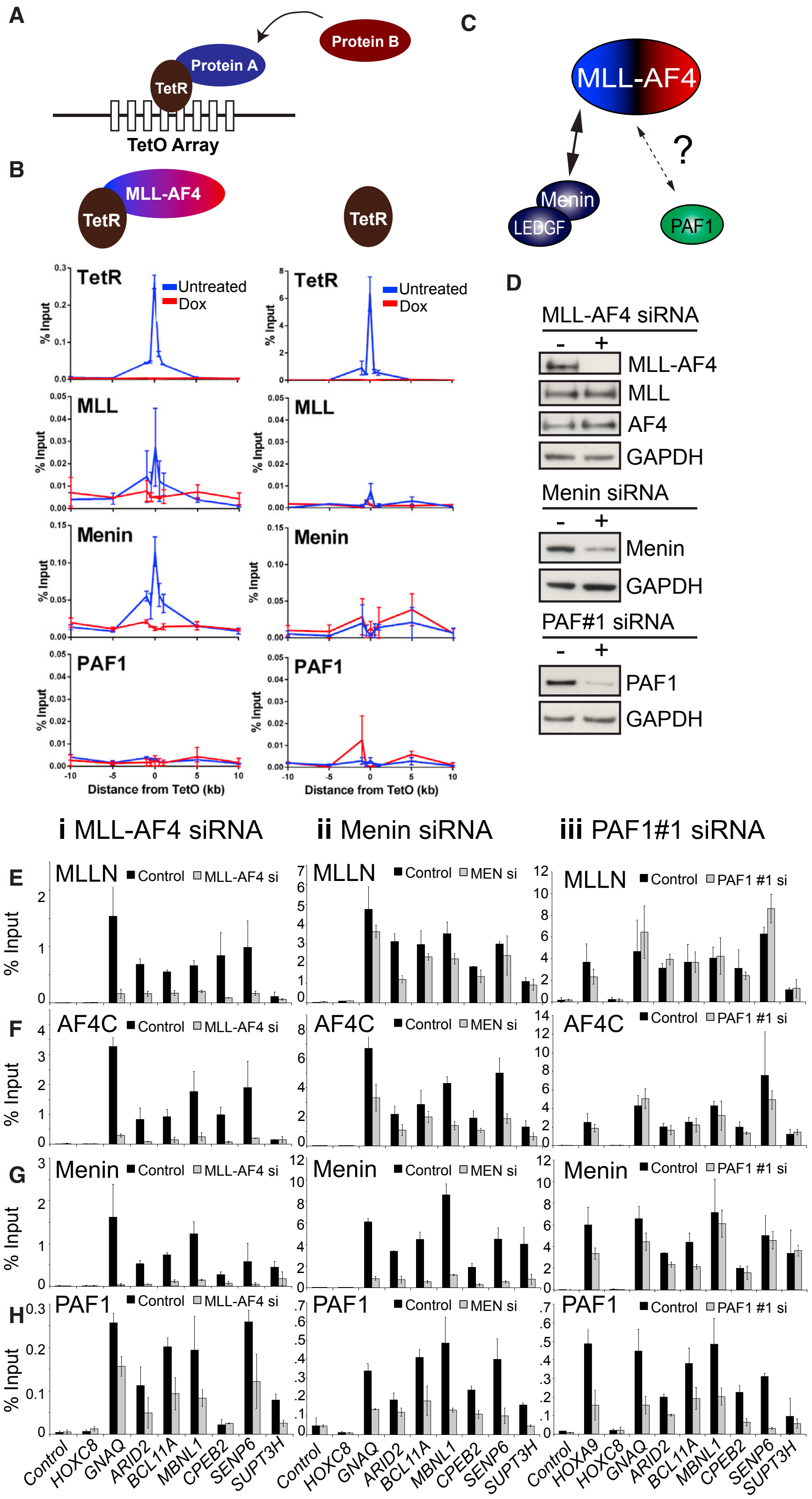

Figure 2. The MLL-AF4:Menin Interaction Is Sufficient but Not Necessary for Recruitment

(A) The Tet-repressor (TetR) recruitment system. An array of Tet-operator (TetO) sequences was centrally inserted into a BAC lacking known promoter, enhancer, or uCpG features, and the BAC was inserted into chromosome 8 of mouse embryonic stem cells (mESCs) (Blackledge et al., 2014). Proteins of interest fused to the TetR can be anchored at the TetO array. The TetR-TetO interaction can be disrupted with doxycycline treatment, allowing one to test whether recruitment of a specific protein is dependent on the continuous presence of a particular TetR fusion.

(B) ChIP-qPCR showing the binding of TetR-MLL-AF4 (using FS2 [TetR] and MLL(N) antibody), Menin, and PAF1 in TetO mESCs transfected with TetR-MLL-AF4 (left panel) and in TetR-only control mESCs (right panel). Error bars represent the SD of two biological replicates. Red line, with doxycycline.

(C) The TetR experiments indicate that there is a strong interaction between MLL-AF4 and Menin and an undetectable interaction between MLL-AF4 and PAF1.

(D) SEM cells were treated with MLL-AF4, Menin, or PAF1 siRNAs, and individual representative western blots from the experiments in E–H are shown.

(E–H) MLL-N (E), AF4-C (F), Menin (G), and PAF1 (H) ChIP in control (black bars) and siRNA-treated (gray bars) SEM cells as follows: column i, MLL-AF4 siRNA; column ii, Menin siRNA; and column iii, PAF1 siRNA. Note that the control samples are the same between PAF1#1 siRNA and PAF1#2 siRNA (see Figure S2H) experiments as these were performed in parallel. Error bars represent the SD of at least three biological replicates.

See also Figure S2.

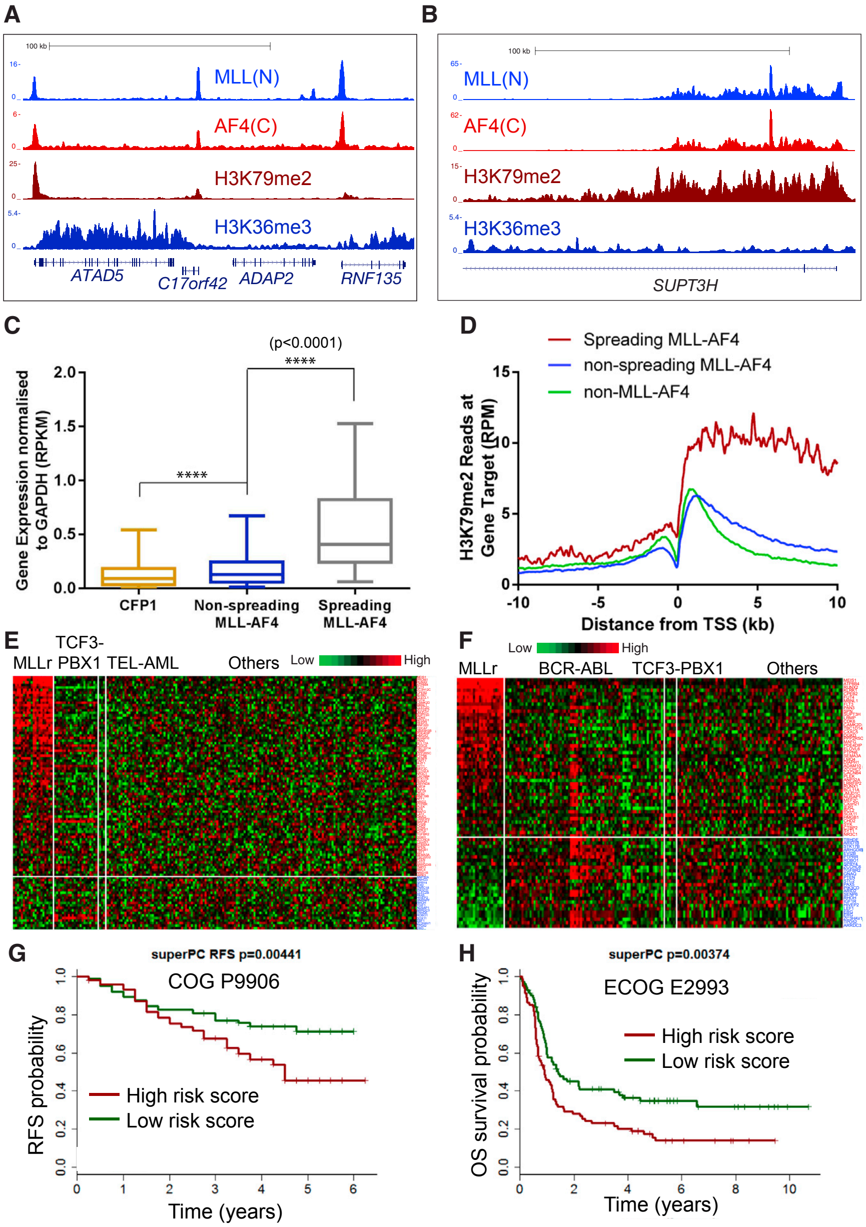

Figure 3. MLL-AF4 Spreading Marks a Subset of Highly Expressed Genes

(A and B) Example ChIP-seq tracks showing promoter-restricted (A) or spreading (B) of MLL-AF4, H3K79me2, and H3K36me3 in SEM cells.

(C) Box-and-whisker plot showing the median and interquartile (IQ) range of gene expression of spreading MLL-AF4 gene targets (n = 149) compared to non-spreading MLL-AF4 targets (n = 2,878) and CFP1 targets (n = 6,147). Gene expression, normalized to *GAPDH* expression, is derived from four biological replicates of nascent RNA-seq in SEM cells. ****p < 0.0001, two-tailed Mann-Whitney U test.

(D) Composite binding plot of H3K79me2 ChIP-seq reads at the TSS of gene targets of spreading MLL-AF4 (red), non-spreading MLL-AF4 (blue), and non-MLL-AF4 targets that are marked by H3K79me2 (green).

(E and F) Heatmap expression data showing overexpression of 79% (E, COG P9906 patients [Harvey et al., 2010]) or 64% (F, ECOG 2993 patients [Geng et al., 2012]) of SEM spreading targets in MLL patients (MLLr) compared to the ALL patient subsets indicated.

(G and H) Super-PC analysis (Bair and Tibshirani, 2004) using the spreading-gene target list showing relapse-free survival (RFS) of ALL patients (G, COG P9906 [Harvey et al., 2010]) and overall survival (OS) of ALL patients (H, ECOG 2993 [Geng et al., 2012]) classified by either high- or low-risk scores computed using the spreading MLL-AF4 gene targets in a super-PC model; see Supplemental Experimental Procedures, Survival Analysis, for details.

See also Figure S3.

that the H3K79me2 pattern observed is a consequence of spreading MLL-AF4.

Because broad MLL-FP binding domains have been observed previously (Bernt et al., 2011; Guenther et al., 2008), one possibility is that spreading identifies bona fide MLL-AF4 target genes, whereas non-spreading peaks represent wild-type MLL and AF4 co-bound sites. To test this, we separated the MLL-AF4 siRNA nascent RNA-seq and ChIP-seq datasets (Figures S1A–S1D) into spreading and non-spreading target sets. We found that almost all spreading and non-spreading targets are bound by MLL-AF4, but spreading targets are more likely to be downregulated by a loss of MLL-AF4 (Figures S3E and S3F). A recently generated FLAG tagged MLL-Af4 ChIP-seq experiment in $CD34^+$ cord blood cells (Lin et al., 2016) allowed us to unambiguously identify MLL-Af4 binding sites in a primary transformed cell. FLAG-MLL-Af4 ChIP-seq identified almost 3,000 MLL-Af4 gene targets, similar to the number we obtained in SEM cells (Lin et al., 2016). FLAG-MLL-Af4 binding could be divided into both spreading and non-spreading targets, about 40%–50% of which overlapped with MLL-AF4 targets in SEM cells (Figures S3G–S3I). Taken together, this suggests that MLL-AF4 can display both spreading and non-spreading binding patterns, but spreading gene targets are less common and are more significantly associated with a dependence on MLL-AF4 for their activation.

In order to better understand the significance of our spreading target set, we analyzed the expression profile of SEM spreading targets in two different patient cohorts and found that 64%–79% of SEM spreading targets are overexpressed in MLLr ALL patients (Figures 3E and 3F). Using a super-PC analysis (Bair and Tibshirani, 2004), we also found that there is a signature within the spreading target set that is predictive of a poor prognosis in patients (Figures 3G and 3H). Thus, MLL-AF4 spreading targets also have clinical significance in patients.

Spreading Is Common among MLL Fusion Proteins but Not Wild-Type MLL

Because spreading is an important feature of MLL-AF4 binding, we investigated how common spreading is for other MLL-FPs. MLL(N) ChIP-seq in the MLL-AF6 cell line ML-2 detects the fusion protein unambiguously due to a deletion of the wild-type *MLL* allele. Spreading for MLL-AF6 was observed at 47 (43.1%) gene target isoforms (Figure 4A; Table S3), and similar to MLL-AF4, these spreading targets displayed a significant increase in H3K79Me2 compared to non-spreading MLL-AF6 targets (Figure 4B). The high percentage of spreading peaks within the MLL-AF6 set is due to the low number (109) of total MLL-AF6 binding events in ML-2 cells. Using MLL(N) ChIP-seq, spreading was also observed in MV4;11 (MLL-AF4), KOPN-8 (MLL-ENL), and THP-1 (MLL-AF9) MLLr cell lines (Figures 4C and 4D; Table S1). ER-tagged MLL-ENL (Garcia-Cuellar et al., 2016), biotin-tagged MLL-AF9 (Bernt et al., 2011), MLL-AF4 in MV4;11 cells (Zhu et al., 2016), MLL-AF4 in patient cells (this study), and FLAG-MLL-Af4 in $CD34^+$ cells (Lin et al., 2016) also displayed spreading (Figures 4E, 4F, and S4A–S4E). The spreading pattern of MLL-AF4 in SEM cells often closely resembles the spreading pattern of MLL-AF4 in patient cells or in FLAG-MLL-Af4 cells at common gene targets (Figures S4D and S4E), suggesting there may be a common mechanism for spreading among these diverse samples.

Importantly, wild-type MLL spreading is not observed in non-MLLr leukemia cell lines (RCH-ACV or CCRF-CEM), at wild-type MLL binding sites in SEM cells (Figures 4C and 4D), or for wild-type MLL(C) in MV4;11 cells (Zhu et al., 2016) (Figure S4C). MLL-AF4 displays spreading in primary patient cells, but there was no wild-type MLL spreading in the relevant normal human hematopoietic cells from either cord blood (CB) or second-trimester fetal bone marrow (FBM) (Figures 4E, 4F, S4A, and S4D). These results show spreading is specifically associated with MLL-FPs.

Some Individual Spreading Gene Targets Have Altered Gene Expression, Reduced DNA Methylation Patterns, and Individual Poor Prognoses in Patients

To better understand the clinical significance of individual spreading targets, we analyzed nine common targets from five MLLr leukemia cell lines (Figure 4G; Tables S1 and S4). High expression of *ARID2*, *JMJD1C*, *MBNL1*, *MEF2C*, or *RUNX2* alone is associated with at least one indicator of poor prognosis in ALL patients (Table S4), and *CPEB2*, *MBNL1*, *RUNX2*, and *ZEB2* are all specifically overexpressed in MLL-FP leukemias (Figures 4H and S5A; Table S5). Interestingly, *RUNX2*, *MBNL1*, *JMJD1C*, *SENP6*, *MEF2C*, and *ZEB2* are also hypomethylated in MLL-FP samples compared to either normal cells or other leukemias (Figure S5B; not shown). Although these data show that some MLL-FP spreading targets can individually have an important role in human leukemias, there does not seem to be a single key set of spreading targets that are necessarily found in all MLL-FP samples. However, taken in total, the data show that MLL-FP spreading is an indicator of particularly significant MLL-FP activity.

Spreading Correlates with Menin and MLL-AF4 Complex Components

To better understand MLL-FP spreading, we analyzed whether spreading is related to other MLL-AF4 complex components (Figure 5A). When all MLL-AF4 spreading targets were sorted by length, the ChIP-seq signal of MLL(N) and AF4(C) generated a characteristic curve shape (Figure 5B, panels 1 and 2). Interestingly, MLL-AF4 spreading is punctuated by uCpG sites, with the beginning and end of spreading domains demarcated by uCpG sites (Figures 5A and 5B, panel 3). This indicates a role for the CXXC domain in stabilizing spreading and agrees with the hypomethylation observed at spreading MLL-AF4 targets in patients (Figure S5B). This is an important role for the CXXC domain within the context of MLL-AF4 because neither KDM2B nor CFP1 showed the same spreading pattern, even though they both bind to uCpGs (Figures 5A and S6A). The majority of uCpG regions under spreading peaks were within 1–2 kb of each other and rarely exceeded 4 kb, with 7 kb being the greatest distance observed (Figure S6B). Therefore, the proximity of uCpG sites to each other under the spreading peaks appears to be important and may be a limiting factor in determining the degree of spreading. If true, this also suggests that spreading may be non-random, and only genes with a clustered uCpG landscape downstream of their promoter are amenable to spreading.

Spreading was not simply a result of an association with basal transcription factors because neither RNAPII nor members of PAFc showed the same spreading pattern; instead, they extended beyond the spreading domain to the end of the gene (Figures 1C, 5A, and S6C). Conversely, both Menin and ENL displayed identical spreading patterns to MLL-AF4 (Figures 1C, 5A and 5B, panels 4 and 5). Our observation that Menin knockdowns reduce MLL-AF4 binding at spreading gene targets (Figures 2E–2G) supports the idea that there is a role for Menin in stabilizing spreading. In conclusion, we envisage a model whereby CXXC-mediated weak binding of the fusion protein at low-CG density uCpG sites in the gene body can be stabilized by CXXC-mediated recruitment to the CG-rich uCpGs at promoters (Figure 5C). However, this depends on the weak binding sites occurring in close proximity to the promoter and each other, with Menin or ENL facilitating stabilization through a bridging mechanism (Figure 5C).

Spreading MLL-AF4 Represents a Subset of Broad H3K4me3 Distinct from Super-Enhancers

Several recent studies have characterized broad binding chromatin domains as markers of functional significance, including super-enhancers (Lovén et al., 2013; Whyte et al., 2013) and broad regions of H3K4me3 (Benayoun et al., 2014). Whereas genes associated with super-enhancers were shown to correlate with increased expression, genes marked by broad H3K4me3 showed an increase in transcriptional consistency, i.e., less variation in transcription rate between replicates as determined by RNA-seq and nascent RNA-seq, as well as an increase in gene expression (Benayoun et al., 2014). Here, we wanted to determine whether MLL-AF4 spreading domains were related to either super-enhancers or broad H3K4me3 peaks.

First, we characterized super-enhancers and broad H3K4me3 domains in SEM cells using the same criteria as the original studies (Figures S6D–S6G). Almost all regions of spreading MLL-AF4 were distinct from super-enhancers, but the majority (87%) of spreading MLL-AF4 gene targets were a subset of

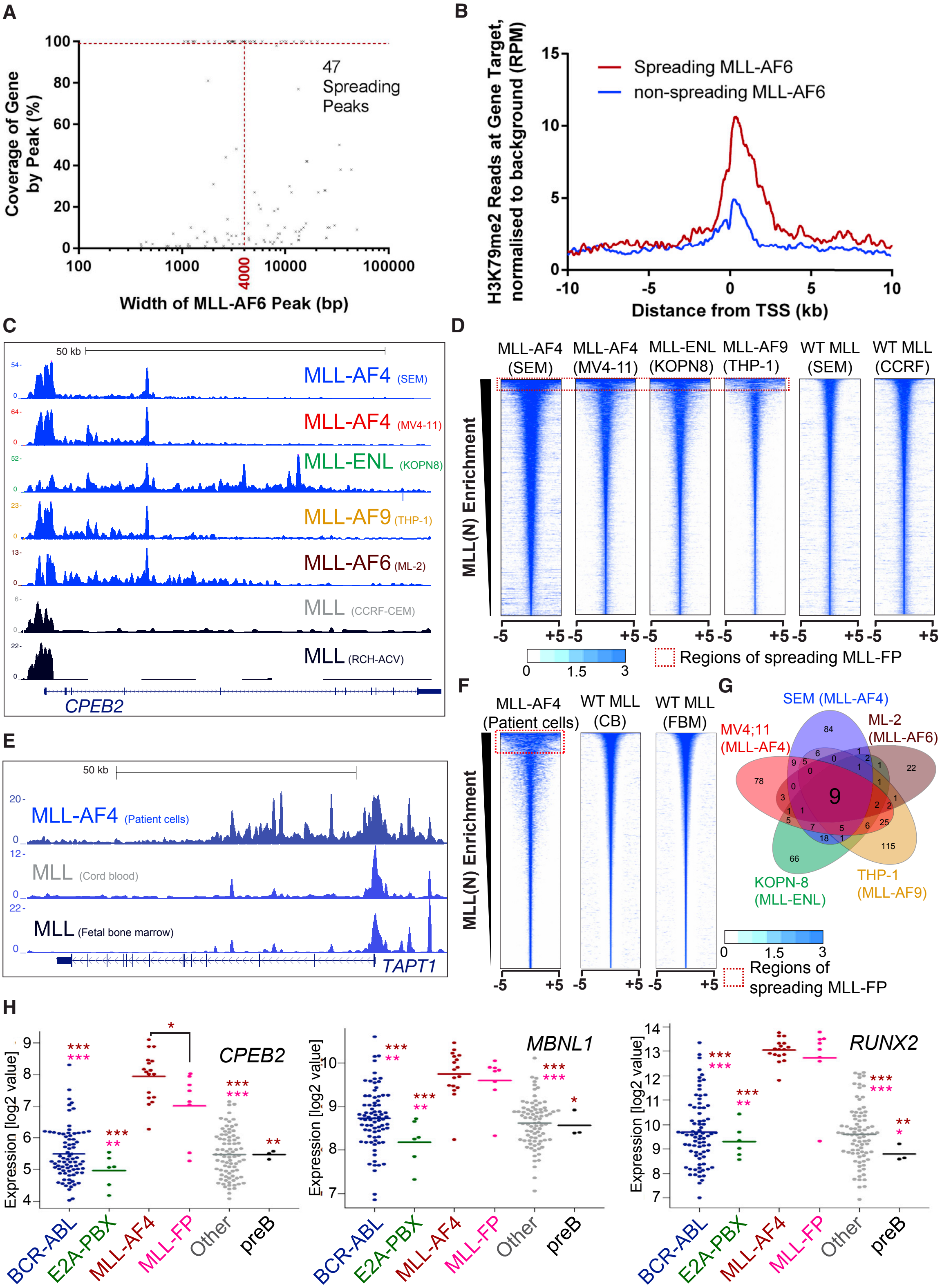

Figure 4. MLL-FP Spreading Occurs in Multiple in MLL Leukemias

(A) Spreading MLL-AF6 peaks were defined as peaks that extend greater than 4 kb from the TSS into the gene body without going beyond the end of the gene. Using these criteria, 47 spreading MLL-AF6 peaks were identified in ML-2 cells (Table S2).

(legend continued on next page)

broad H3K4me3 gene targets (Figure 5D). Despite being distinct from super-enhancers, spreading MLL-AF4 correlated with MED1 and BRD4 binding as well as H3K27Ac (Figure 5E, panels 1–3). The major difference between MLL-AF4 spreading domains and super-enhancers was the lack of H3K4me1 enrichment; instead, spreading overlaps with H3K4me3 and H3K79me2 (Figure 5E, panels 4–6).

Similar to past work (Benayoun et al., 2014), gene targets of the 5% broadest H3K4me3 peaks in SEM cells showed a significant increase in transcriptional consistency compared to genes marked by the remaining 95% of H3K4me3 peaks ($p < 0.0001$, Mann-Whitney U test, Figure S6H). As a whole, gene targets of MLL-AF4 showed a significant increase in transcriptional consistency compared to H3K4me3-marked genes ($p < 0.0001$, Mann-Whitney U test; Figure S6I), suggesting that maintaining gene regulation within narrow limits could be an important property of MLL-AF4 controlled gene expression crucial for the leukemia. However, spreading MLL-AF4 gene targets did not show increased transcriptional consistency compared to all MLL-AF4 gene targets (Figure S6I); thus, this was not a feature specific to spreading. Based on the signature of histone marks and protein associations, spreading MLL-AF4 represents a hybrid of broad H3K4me3 domains and super-enhancers, with transcriptional properties such as high expression (Figure 3C) more similar to super-enhancers.

Gene Targets of Spreading MLL-AF4 Display Increased Sensitivity to DOT1L Inhibition

If it is possible to target spreading MLL-AF4 target genes, it is likely that this would have a strong and specific effect on the inhibition of leukemia maintenance. Because spreading MLL-AF4 targets are marked with high levels of H3K79me2 (Figure 3D), we wanted to determine whether they are particularly sensitive to the DOT1L inhibitor EPZ-5676 (Daigle et al., 2013). Treatment of SEM cells with 2 μM EPZ-5676 for 7 days produced an almost complete loss of the H3K79me3 mark for all genes tested (Figure S7A). Using nascent RNA-seq, we identified 2,462 downregulated genes, 84% of which were marked by H3K79me2 (Figure 6A) and that included a number of spreading targets (e.g., *CDK6*; Figure 6B). As a group, over 50% of spreading targets were downregulated following EPZ-5676 treatment compared to only 16% of non-spreading MLL-AF4 targets and 23% of H3K79me2-marked genes ($p < 0.0001$, two-tailed Fisher's exact test; Figure 6C). Furthermore, spreading MLL-AF4 targets were among those that showed the greatest downregulation, even compared to genes that had similar levels of high expression (Figure 6D). Spreading MLL-AF4 gene targets were also significantly more downregulated in response to EPZ-5676 compared to non-spreading MLL-AF4 gene targets ($p < 0.0001$, Mann-Whitney U test; Figure 6E). Therefore, spreading MLL-AF4 targets are among the most sensitive to treatment with EPZ-5676 when compared to all other genes. Using a randomly selected group of genes with levels of H3K79me2 similar to those of spreading MLL-AF4 targets (Figure S7B), we also found that spreading MLL-AF4 targets were significantly more downregulated after EPZ-5676 treatment (Figure S7C, top and bottom; $p < 0.0001$ and $p < 0.01$, respectively; Mann-Whitney U test). Therefore, genes marked by spreading MLL-AF4 show increased sensitivity to EPZ-5676 through a mechanism not simply determined by high levels of H3K79me2. Interestingly, spreading targets that overlapped with either broad H3K4me3 or super-enhancers were significantly more sensitive to DOT1L inhibition than spreading MLL-AF4 targets alone (Figure S7D). This indicates that there are further subdivisions of activity within spreading targets themselves, something that may explain recent results looking at DOT1L and BRD4 cooperation (Gilan et al., 2016).

As well as being particularly sensitive to a loss of H3K79me2/3, spreading MLL-AF4 gene targets were significantly downregulated compared to non-spreading and non-MLL-AF4 targets by MLL-AF4 siRNA treatment ($p < 0.0001$, two-tailed Mann Whitney U test; Figure 6F). Therefore, the increased gene expression observed at MLL-AF4 spreading targets is significantly linked to both MLL-AF4 and H3K79me2 and is more likely to be downregulated by DOT1L inhibition.

Sensitivity of Spreading Gene Targets Provides a Rationale for Combination Therapy Using DOT1L Inhibitors

It seems unlikely that a single drug alone will be effective in treating MLL-AF4 leukemias. Even among MLL-AF4 spreading targets, some gene targets have an increased sensitivity to a loss of H3K79me2 (Figures 7A and 7B; Table S6). We recently showed that the BCL-2-specific protein inhibitor ABT-199 synergizes with DOT1L inhibitors (Benito et al., 2015), potentially because BCL-2 protein levels are not strongly affected by DOT1L inhibitor concentrations that affect more sensitive targets such as CDK6 or BCL11A (Figure 7B). Because Menin is partly responsible for spreading (see Figures 2E–2G and 5A–5C), the

(B) Composite binding plot of H3K79me2 ChIP-seq reads at the TSS of gene targets of spreading MLL-AF6 (red) and non-spreading MLL-AF6 (blue) in ML-2 cells.
(C) Example ChIP-seq tracks of MLL(N) in MLL-FP and germline MLL cell lines.
(D) Heatmaps of MLL(N) ChIP-seq reads from different MLL-FP cell lines as well as wild-type MLL in SEM cells and in non-MLLr cell lines. Red dotted line indicates spreading across a 10-kb window. Scale bar represents tags per base pair per 10^7 reads.
(E) Example ChIP-seq tracks of MLL(N) showing spreading in MLL-AF4 patient cells (top) compared to wild-type MLL in mononuclear cells derived from cord blood (middle) and fetal bone marrow (bottom).
(F) Heatmaps showing MLL(N) ChIP-seq reads from the experiments in (E); scale and red line as in (D).
(G) Venn diagram showing the overlap between gene targets of spreading MLL(N) ChIP-seq several MLLr cell lines.
(H) *CPEB2*, *MBNL1*, and *RUNX2* are overexpressed in MLL-AF4 and other MLL-FP patients compared to different patient samples and normal pre-B cells. Each dot indicates an individual patient sample. Data are taken from an ECOG E2993 clinical trial (Geng et al., 2012). Dark red asterisk (*) indicates a significant difference compared to MLL-AF4, and pink asterisk (*) indicates a significant difference compared to the MLL-FP group (which includes MLL-ENL [6], MLL-AF9 [1], and MLL-EPS15 [1]). ***$p < 0.001$, **$p < 0.01$, *$p < 0.05$. A two-tailed Wilcoxon test was used to calculate p values, and p values for the different comparisons are listed in Table S5.
See also Figures S4 and S5.

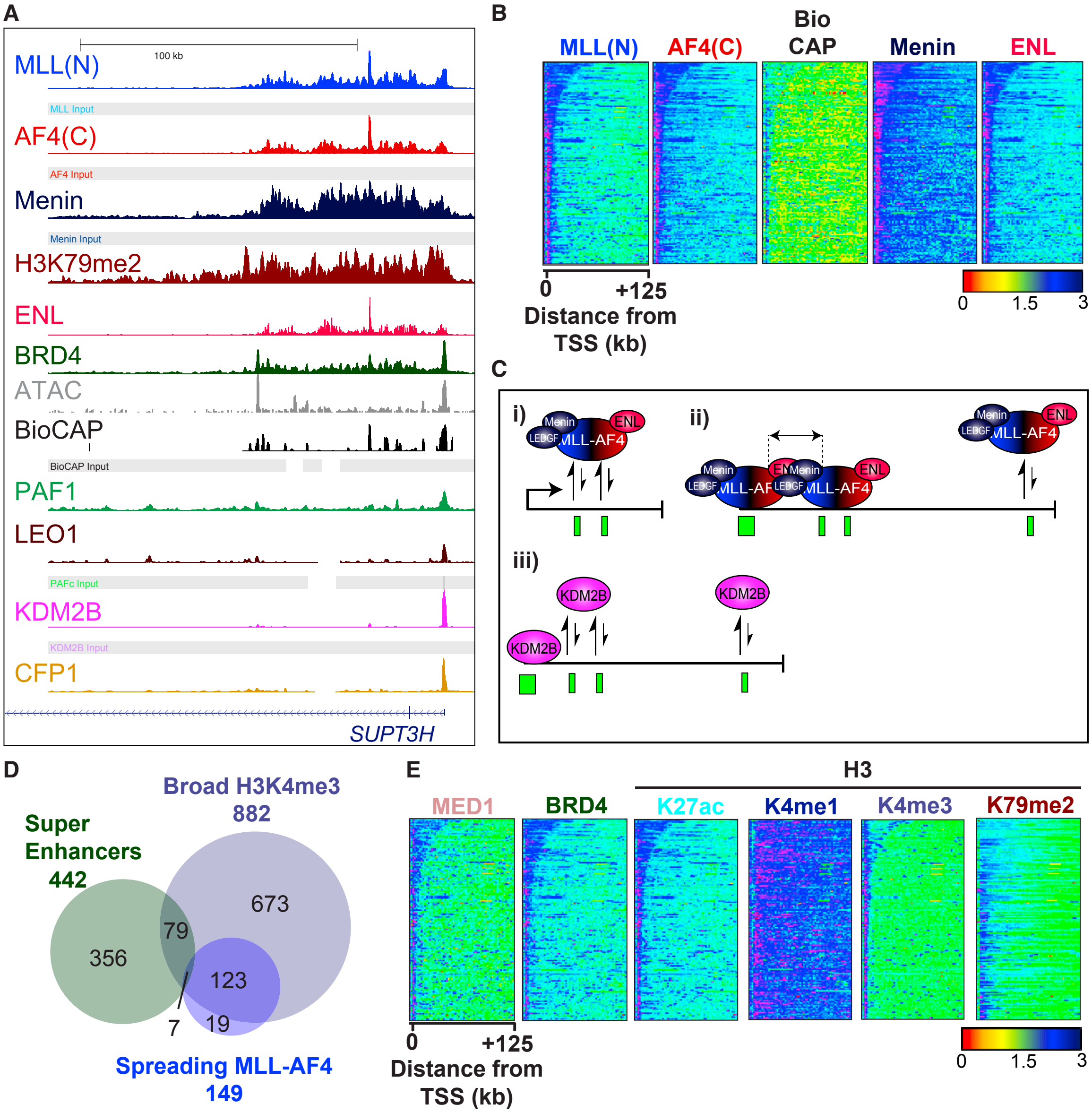

Figure 5. Spreading Correlates with Members of the Menin:LEDGF and Super-Elongation Complexes

(A) Example ChIP-seq tracks at *SUPT3H* in SEM cells.

(B) Heatmap of MLL-AF4, Bio-Cap, Menin, and ENL signal at all 149 spreading MLL-AF4 targets, ordered by length of spreading peak. Scale bar represents tags per bp per 10^7 reads.

(C) Schematic showing a proposed model for spreading across uCpG regions by MLL-FPs. (i) In the absence of promoter-bound MLL-AF4, CXXC-mediated recruitment of the fusion protein to uCpG-poor regions in the gene body are not stabilized. (ii) Stable CXXC-mediated recruitment to uCpG-rich promoter regions can stabilize nearby MLL-AF4 recruitment at gene body uCpG regions due to common interactions with complex members such as Menin and ENL, whereas distal recruitment events remain unstable. (iii) Because other CXXC proteins, such as KDM2B, do not interact with complex member such as Menin or ENL, promoter-bound KDM2B is not sufficient to stabilize neighboring CXXC-mediated recruitment to CpG-poor uCpG regions in the gene body.

(D) Venn diagram showing the overlap between gene targets of super-enhancers, broad H3K4me3 peaks, and spreading MLL-AF4, in SEM cells.

(E) Heatmap showing ChIP-seq reads of the components indicated at all 149 spreading MLL-AF4 gene targets in SEM cells; scale bar as in (B).

See also Figure S6.

use of Menin inhibitors represents another way to target sensitive MLL-AF4 spreading genes (see Figure S7E). In an extension of our earlier work, we find that there is a strong synergy between ABT-199 and the DOT1L inhibitors EPZ5676 and SGC0946, as well as a strong synergistic interaction between the Menin inhibitor MI-503 (Borkin et al., 2015) and ABT-199 (Figures 7C–7F; combination index [CI] < 1; calculations as described in Milella et al. [2002]). Thus, carefully choosing different drug combinations may increase their effectiveness at disrupting MLL-FP leukemic growth.

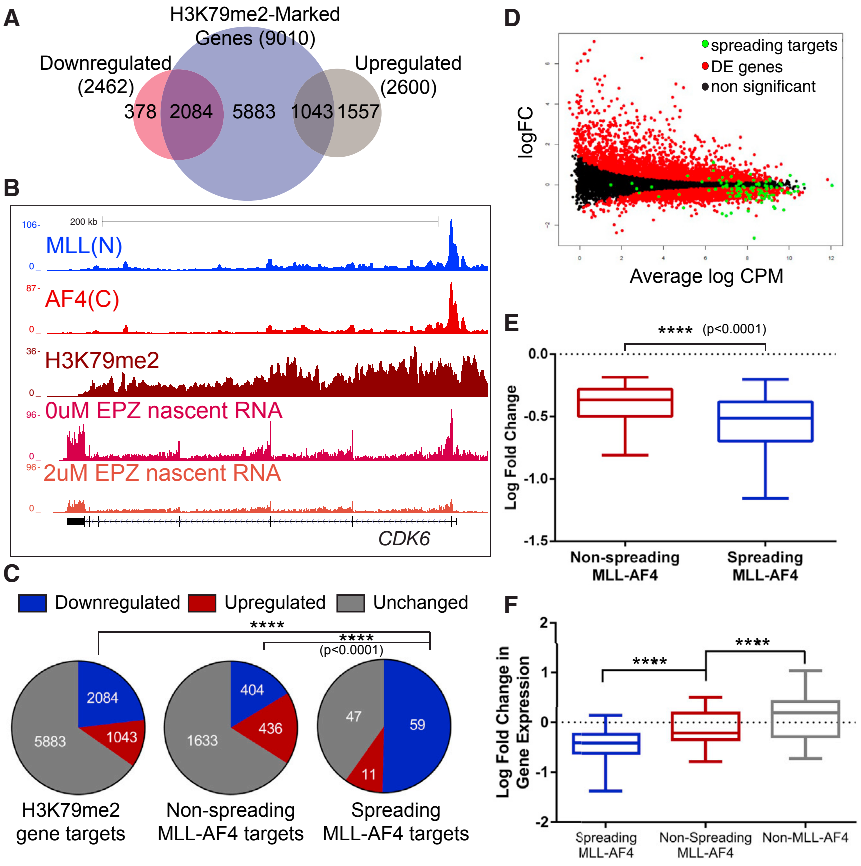

Figure 6. Spreading MLL-AF4 Targets Show Increased Sensitivity to DOT1L Inhibition

(A) Venn diagram showing an overlap between H3K79me2-marked genes and upregulated and downregulated genes in SEM cells following treatment with 2 μM EPZ-5676.

(B) Example ChIP-seq tracks at *CDK6* and nascent RNA-seq in control (0 μM) and 2 μM EPZ-5676-treated SEM cells.

(C) Pie charts showing the proportion of genes that are significantly downregulated (blue), upregulated (red), or remain unchanged (gray), among H3K79me2-marked genes (left), non-spreading MLL-AF4 gene targets (center), and spreading MLL-AF4 gene targets (right), following treatment of SEM cells with 2 μM EPZ-5676. ****$p < 0.0001$, Fisher's exact test.

(D) Smear plot showing the fold change in gene expression of all genes in SEM cells following treatment with 2 μM EPZ-5676 compared to their expression level (CPM). Black, non-significant change in gene expression; red, differentially expressed gene; green, spreading MLL-AF4 gene targets.

(E) Box-and-whisker plot showing the median and IQ range of fold change in expression of all significantly downregulated gene targets of non-spreading MLL-AF4 (red) compared to spreading MLL-AF4 (blue), after 2 μM EPZ-5676 treatment in SEM cells. ****$p < 0.0001$, Mann-Whitney U test.

(F) Box-and-whisker plot showing the median and IQ range of fold change in expression of all significantly affected spreading MLL-AF4 (blue), non-spreading MLL-AF4 (red), and non-MLL-AF4 gene targets following siRNA-mediated knockdown of *MLL-AF4* in SEM cells. ****$p < 0.0001$, Mann-Whitney U test.

See also Figure S7.

DISCUSSION

In this study, we have found that uCpGs strongly correlate with the highest occupancy of MLL-AF4 binding and that MLL-AF4 and Menin co-stabilize each other's binding to gene targets. This mirrors recent findings of co-dependent stabilization between wild-type MLL and LEDGF (Zhu et al., 2016), which would suggest an independent role for LEDGF in wild-type MLL function, as previous reports indicate that Menin and wild-type MLL regulate distinct gene targets (Li et al., 2013). Zhu et al. have also shown that loss of LEDGF actually increases MLL-FP recruitment (Zhu et al., 2016), which is partly supported by our *Ledgf* knockdown experiment (Figure S2G). We have also shown that there is no direct connection between MLL-AF4 recruitment and PAFc, suggesting that past observations of MLL-FP dependence on PAFc (Milne et al., 2010; Muntean et al., 2010) may have been due to indirect effects, or perhaps PAFc is only required for binding of MLL-FPs at specific gene targets such as *HOXA9*.

Previous studies have indicated that MLL-FP binding can be associated with broad chromatin domains (Bernt et al., 2011; Guenther et al., 2008) or divided into two classes, with 5′ binding indicating a dependence on H3K79me2 (Garcia-Cuellar et al., 2016). Our results suggest that it is not the presence of MLL-AF4 and H3K79me2/3 that is most predictive of a dependence on H3K79me2, but the presence of MLL-AF4 spreading. Our observations also show that spreading strongly correlates with Menin and ENL binding and occurs across uCpG landscapes in the gene body that are within close proximity to the gene promoter. Unmethylated CpG regions in gene bodies typically display a relatively low CG density, which is possibly why we do not observe other CXXC proteins binding in the gene body. Therefore, spreading of MLL-FPs may be made possible by Menin/ENL-mediated stabilization at gene body uCpG regions where uCpG density is too low for a strong CXXC-uCpG interaction. Although wild-type MLL can also interact with Menin, a co-operation with fusion partner proteins such as ENL may generate complexes that permit MLL-FP dimerization (Mueller et al., 2007; Yokoyama et al., 2010) and through this mechanism create a spreading domain of MLL-FP that is anchored by CXXC-uCpG interactions in close proximity to each other, something that is unavailable to wild-type MLL.

It is unknown whether an ability to spread into the body of particular gene targets drives higher expression and initiates progression of leukemia or whether these gene targets are

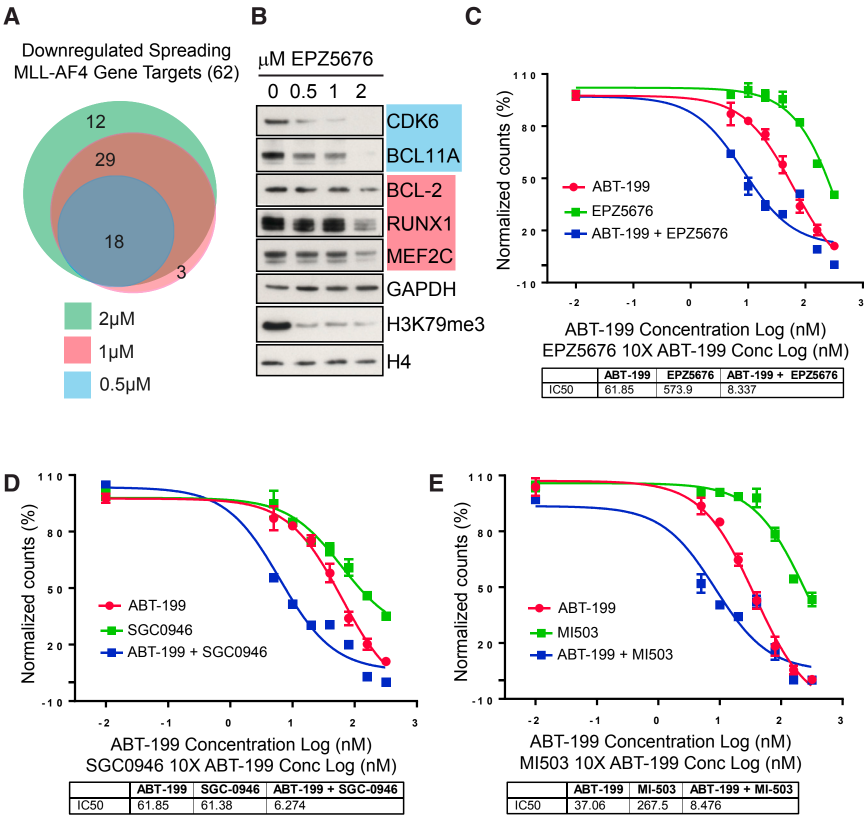

F

Drug	ED50	ED75	ED90	CI
ABT-199 + EPZ5676	0.25	0.31	0.38	0.31
ABT-199 + SGC0946	0.21	0.21	0.21	0.21
ABT-199 + MI503	0.21	0.20	0.19	0.20

Figure 7. Sensitivity of Spreading Gene Targets Provides a Rationale for Combined Therapy Using DOT1L Inhibitors

(A) Venn diagram showing the overlap of spreading MLL-AF4 gene targets that are downregulated as measured by nascent RNA-seq following 0.5 μM (blue), 1 μM (red), and 2 μM (green) EPZ-5676 treatment.

(B) Western blot showing the protein expression of several spreading MLL-AF4 targets and controls in the presence of control, 0.5, 1, or 2 μM EPZ-5676 treatments. Blue and red boxes relate to treatment colors in (A) that led to the lowest level of treatment that resulted in reduced gene transcription.

(C–E) A cell viability assay of SEMK2 cells treated with a DMSO control, different concentrations of ABT-199 (320, 160, 80, 40, 20, 10, and 5 nM, and DMSO control) alone, or in combination with a 1:10 ratio of either EPZ5676 (C), SGC0946 (D), or MI503 (E) (3,200, 1,600, 800, 400, 200, 100, and 50 nM, and DMSO control).

(F) A tabular summary of the combination index for the different drug treatments calculated as in Milella et al. (2002).

already highly expressed and the active chromatin landscape is simply a pre-requisite for facilitating spreading. Nevertheless, in the context of these remaining questions, our study has revealed that spreading of MLL-AF4 defines the expression of a subset of genes that are important for leukemia and are characterized by gene activation that is predictive of a poor prognosis. These target genes are particularly sensitive to DOT1L inhibition, which provides a new molecular rationale for the specificity of DOT1L or Menin inhibition in MLL-AF4 leukemias, and the possibility of combining this with drugs that target less sensitive spreading targets such as those that target BCL-2.

EXPERIMENTAL PROCEDURES

Cell Lines, Cultures, and Drug Treatment Studies

Cell lines, culture methods, and drug treatment protocols used in this study are listed in Supplemental Experimental Procedures. CB was collected under the auspices of a National Research Ethics Service-approved study with written informed consent. Human fetal bone samples were obtained through the Human Development Biology Resource (http://www.hdbr.org).

Western Blot Analysis

Western blot analysis was performed as previously described (Wilkinson et al., 2013). Antibodies used for western blot analysis are listed in Supplemental Experimental Procedures.

ChIP Assays and ChIP-Seq

ChIP and ChIP-seq experiments were performed as described in Supplemental Experimental Procedures and as previously described (Benito et al., 2015; Wilkinson et al., 2013).

TetR Recruitment System

For the TetR recruitment assay, we used the previously engineered Tet-operon (TetO) mESC line (Blackledge et al., 2014). *MLL-AF4*, *Menin*, and *PAF1* cDNA were inserted downstream of the FS2-TetR coding sequence in the original pCAGFS2TetR vector, by ligation-independent cloning (LIC). Plasmids were transfected into TetO mESCs using Lipofectamine 2000, and clones stably expressing TetR fusions were selected using puromycin (1 μg/mL), or *MLL-AF4* cDNA was transiently transfected into mESCs at 60%–70% confluency using Lipofectamine 2000. Cells were collected 24 hr after transfection.

Gene Targets and Spreading Peaks

ChIP-seq peaks were called as described in Supplemental Experimental Procedures. Gene targets were defined as any gene where the transcription start site (TSS) overlapped directly with a peak. A peak was classed as spreading if it overlapped the TSS of a gene and extended over 4 kb from the TSS into the gene body without going beyond the transcription end site (TES) of the gene. If a peak exhibited spreading at two different genes (or isoforms with different TSS co-ordinates), both spreading-gene pairs were kept.

Nascent RNA-Seq

Nascent RNA-seq and gene expression analysis was performed as described in Supplemental Experimental Procedures.

Survival Analysis

Clinical datasets and survival analyses are detailed in Supplemental Experimental Procedures.

Statistics

Data were analyzed using Fisher's exact test, Wilcoxon test, and Mann-Whitney U test, where appropriate. Results were deemed significant if $p < 0.05$. Unless otherwise indicated, data are shown as mean ± SD. This paper analyzed

datasets from GEO: GSE13313, GSE28460, GSE29130, GSE34861, GSE73528, GSE74812, and GSE84116; and ArrayExpress: E-MTAB-3593 (for a detailed list of datasets, see Table S7).

ACCESSION NUMBERS

The accession number for the datasets reported in this paper is GEO: GSE83671.

SUPPLEMENTAL INFORMATION

Supplemental Information includes Supplemental Experimental Procedures, seven figures, and seven tables and can be found with this article online at http://dx.doi.org/10.1016/j.celrep.2016.12.054.

AUTHOR CONTRIBUTIONS

Conceptualization, T.A.M. and J.K.; Formal analysis, J.K., E.R., H.G., and H.M.; Investigation, J.K., L.G., M.T., N.P.B., H.M., E.B., T.A.M.; Resources, S.O., F.P., O.H., A.R., I.R.; Writing – Original draft, T.A.M. and J.K.; Writing – Review & Editing, T.A.M., J.K., O.H., A.R., I.R., M.K., R.J.K., H.G.; Visualization, T.A.M. and J.K.; Supervision, T.A.M., M.K., and R.J.K.; Funding Acquisition, T.A.M., O.H., A.R., I.R., R.J.K., M.K.

ACKNOWLEDGMENTS

T.A.M., J.K., L.G., M.T., and E.B. were funded by Medical Research Council (MRC, UK) Molecular Haematology Unit Grant MC_UU_12009/6. The research was also supported by the National Institute for Health Research (NIHR) Oxford Biomedical Research Centre (BRC) Programme. H.G. is supported by a Junior Investigator Award from the UCSF Academic Senate. R.J.K. and N.P.B. are funded by the European Research Council Consolidator Grant 681440 and Wellcome Trust Senior Research Fellowship 098024/Z/11/Z. A.R. is supported by a Bloodwise Clinician Scientist Fellowship 14041 and EHA-ASH Translational Research Training in Hematology Fellowship. I.R. is supported by the University of Oxford, Bloodwise Programme 13001, and the NIHR Oxford BRC. O.H. is supported by a Cancer Research UK programme grant C27943/A12788. The views expressed are those of the author(s) and not necessarily those of the NHS, the NIHR, or the Department of Health. We thank the High-Throughput Genomics Group at the Wellcome Trust Centre for Human Genetics (funded by Wellcome Trust Grant Reference 090532/Z/09/Z) and the Computational Biology Research Group (CBRG), Radcliffe Department of Medicine, at the University of Oxford, supported by the MRC Strategic Award to the Weatherall Institute of Molecular Medicine. The human fetal material was provided by the Joint MRC/Wellcome Trust Grant 099175/Z/12/Z Human Developmental Biology Resource (http://hdbr.org). We thank Nathan Rose for providing us with the purified CXXC protein used for Bio-CAP. We would also like to acknowledge Yale Michaels and Skirmantas Kriaucionis for a critical reading of the manuscript. T.A.M. is one of the founding shareholders of Oxstem Oncology (OSO), a subsidiary company of OxStem Ltd.

Received: June 22, 2016
Revised: October 31, 2016
Accepted: December 16, 2016
Published: January 10, 2017

REFERENCES

Bair, E., and Tibshirani, R. (2004). Semi-supervised methods to predict patient survival from gene expression data. PLoS Biol. *2*, E108.

Ballabio, E., and Milne, T.A. (2012). Molecular and epigenetic mechanisms of MLL in human leukemogenesis. Cancers (Basel) *4*, 904–944.

Benayoun, B.A., Pollina, E.A., Ucar, D., Mahmoudi, S., Karra, K., Wong, E.D., Devarajan, K., Daugherty, A.C., Kundaje, A.B., Mancini, E., et al. (2014). H3K4me3 breadth is linked to cell identity and transcriptional consistency. Cell *158*, 673–688.

Benito, J.M., Godfrey, L., Kojima, K., Hogdal, L., Wunderlich, M., Geng, H., Marzo, I., Harutyunyan, K.G., Golfman, L., North, P., et al. (2015). MLL-rearranged acute lymphoblastic leukemias activate BCL-2 through H3K79 methylation and are sensitive to the BCL-2-specific antagonist ABT-199. Cell Rep. *13*, 2715–2727.

Bernt, K.M., Zhu, N., Sinha, A.U., Vempati, S., Faber, J., Krivtsov, A.V., Feng, Z., Punt, N., Daigle, A., Bullinger, L., et al. (2011). MLL-rearranged leukemia is dependent on aberrant H3K79 methylation by DOT1L. Cancer Cell *20*, 66–78.

Birke, M., Schreiner, S., García-Cuéllar, M.P., Mahr, K., Titgemeyer, F., and Slany, R.K. (2002). The MT domain of the proto-oncoprotein MLL binds to CpG-containing DNA and discriminates against methylation. Nucleic Acids Res. *30*, 958–965.

Biswas, D., Milne, T.A., Basrur, V., Kim, J., Elenitoba-Johnson, K.S., Allis, C.D., and Roeder, R.G. (2011). Function of leukemogenic mixed lineage leukemia 1 (MLL) fusion proteins through distinct partner protein complexes. Proc. Natl. Acad. Sci. USA *108*, 15751–15756.

Blackledge, N.P., Long, H.K., Zhou, J.C., Kriaucionis, S., Patient, R., and Klose, R.J. (2012). Bio-CAP: a versatile and highly sensitive technique to purify and characterise regions of non-methylated DNA. Nucleic Acids Res. *40*, e32.

Blackledge, N.P., Farcas, A.M., Kondo, T., King, H.W., McGouran, J.F., Hanssen, L.L., Ito, S., Cooper, S., Kondo, K., Koseki, Y., et al. (2014). Variant PRC1 complex-dependent H2A ubiquitylation drives PRC2 recruitment and polycomb domain formation. Cell *157*, 1445–1459.

Borkin, D., He, S., Miao, H., Kempinska, K., Pollock, J., Chase, J., Purohit, T., Malik, B., Zhao, T., Wang, J., et al. (2015). Pharmacologic inhibition of the Menin-MLL interaction blocks progression of MLL leukemia in vivo. Cancer Cell *27*, 589–602.

Buenrostro, J.D., Giresi, P.G., Zaba, L.C., Chang, H.Y., and Greenleaf, W.J. (2013). Transposition of native chromatin for fast and sensitive epigenomic profiling of open chromatin, DNA-binding proteins and nucleosome position. Nat. Methods *10*, 1213–1218.

Caslini, C., Yang, Z., El-Osta, M., Milne, T.A., Slany, R.K., and Hess, J.L. (2007). Interaction of MLL amino terminal sequences with menin is required for transformation. Cancer Res. *67*, 7275–7283.

Daigle, S.R., Olhava, E.J., Therkelsen, C.A., Basavapathruni, A., Jin, L., Boriack-Sjodin, P.A., Allain, C.J., Klaus, C.R., Raimondi, A., Scott, M.P., et al. (2013). Potent inhibition of DOT1L as treatment of MLL-fusion leukemia. Blood *122*, 1017–1025.

Farcas, A.M., Blackledge, N.P., Sudbery, I., Long, H.K., McGouran, J.F., Rose, N.R., Lee, S., Sims, D., Cerase, A., Sheahan, T.W., et al. (2012). KDM2B links the polycomb repressive complex 1 (PRC1) to recognition of CpG islands. eLife *1*, e00205.

Garcia-Cuellar, M.P., Büttner, C., Bartenhagen, C., Dugas, M., and Slany, R.K. (2016). Leukemogenic MLL-ENL fusions induce alternative chromatin states to drive a functionally dichotomous group of target genes. Cell Rep. *15*, 310–322.

Geng, H., Brennan, S., Milne, T.A., Chen, W.Y., Li, Y., Hurtz, C., Kweon, S.M., Zickl, L., Shojaee, S., Neuberg, D., et al. (2012). Integrative epigenomic analysis identifies biomarkers and therapeutic targets in adult B-acute lymphoblastic leukemia. Cancer Discov. *2*, 1004–1023.

Gilan, O., Lam, E.Y., Becher, I., Lugo, D., Cannizzaro, E., Joberty, G., Ward, A., Wiese, M., Fong, C.Y., Ftouni, S., et al. (2016). Functional interdependence of BRD4 and DOT1L in MLL leukemia. Nat. Struct. Mol. Biol. *23*, 673–681.

Guenther, M.G., Lawton, L.N., Rozovskaia, T., Frampton, G.M., Levine, S.S., Volkert, T.L., Croce, C.M., Nakamura, T., Canaani, E., and Young, R.A. (2008). Aberrant chromatin at genes encoding stem cell regulators in human mixed-lineage leukemia. Genes Dev. *22*, 3403–3408.

Harvey, R.C., Mullighan, C.G., Wang, X., Dobbin, K.K., Davidson, G.S., Bedrick, E.J., Chen, I.M., Atlas, S.R., Kang, H., Ar, K., et al. (2010). Identification of novel cluster groups in pediatric high-risk B-precursor acute lymphoblastic leukemia with gene expression profiling: correlation with genome-wide DNA copy number alterations, clinical characteristics, and outcome. Blood *116*, 4874–4884.

Jones, B., Su, H., Bhat, A., Lei, H., Bajko, J., Hevi, S., Baltus, G.A., Kadam, S., Zhai, H., Valdez, R., et al. (2008). The histone H3K79 methyltransferase Dot1L is essential for mammalian development and heterochromatin structure. PLoS Genet. *4*, e1000190.

Krivtsov, A.V., Feng, Z., Lemieux, M.E., Faber, J., Vempati, S., Sinha, A.U., Xia, X., Jesneck, J., Bracken, A.P., Silverman, L.B., et al. (2008). H3K79 methylation profiles define murine and human MLL-AF4 leukemias. Cancer Cell *14*, 355–368.

Li, B.E., Gan, T., Meyerson, M., Rabbitts, T.H., and Ernst, P. (2013). Distinct pathways regulated by menin and by MLL1 in hematopoietic stem cells and developing B cells. Blood *122*, 2039–2046.

Lin, S., Luo, R.T., Ptasinska, A., Kerry, J., Assi, S.A., Wunderlich, M., Imamura, T., Kaberlein, J.J., Rayes, A., Althoff, M.J., et al. (2016). Instructive role of MLL-fusion proteins revealed by a model of t(4;11) pro-B acute lymphoblastic leukemia. Cancer Cell *30*, 737–749.

Long, H.K., Blackledge, N.P., and Klose, R.J. (2013). ZF-CxxC domain-containing proteins, CpG islands and the chromatin connection. Biochem. Soc. Trans. *41*, 727–740.

Lovén, J., Hoke, H.A., Lin, C.Y., Lau, A., Orlando, D.A., Vakoc, C.R., Bradner, J.E., Lee, T.I., and Young, R.A. (2013). Selective inhibition of tumor oncogenes by disruption of super-enhancers. Cell *153*, 320–334.

Meyer, C., Hofmann, J., Burmeister, T., Gröger, D., Park, T.S., Emerenciano, M., Pombo de Oliveira, M., Renneville, A., Villarese, P., Macintyre, E., et al. (2013). The MLL recombinome of acute leukemias in 2013. Leukemia *27*, 2165–2176.

Milella, M., Estrov, Z., Kornblau, S.M., Carter, B.Z., Konopleva, M., Tari, A., Schober, W.D., Harris, D., Leysath, C.E., Lopez-Berestein, G., et al. (2002). Synergistic induction of apoptosis by simultaneous disruption of the Bcl-2 and MEK/MAPK pathways in acute myelogenous leukemia. Blood *99*, 3461–3464.

Milne, T.A., Martin, M.E., Brock, H.W., Slany, R.K., and Hess, J.L. (2005). Leukemogenic MLL fusion proteins bind across a broad region of the Hox a9 locus, promoting transcription and multiple histone modifications. Cancer Res. *65*, 11367–11374.

Milne, T.A., Kim, J., Wang, G.G., Stadler, S.C., Basrur, V., Whitcomb, S.J., Wang, Z., Ruthenburg, A.J., Elenitoba-Johnson, K.S., Roeder, R.G., and Allis, C.D. (2010). Multiple interactions recruit MLL1 and MLL1 fusion proteins to the HOXA9 locus in leukemogenesis. Mol. Cell *38*, 853–863.

Mueller, D., Bach, C., Zeisig, D., Garcia-Cuellar, M.P., Monroe, S., Sreekumar, A., Zhou, R., Nesvizhskii, A., Chinnaiyan, A., Hess, J.L., and Slany, R.K. (2007). A role for the MLL fusion partner ENL in transcriptional elongation and chromatin modification. Blood *110*, 4445–4454.

Muntean, A.G., Tan, J., Sitwala, K., Huang, Y., Bronstein, J., Connelly, J.A., Basrur, V., Elenitoba-Johnson, K.S., and Hess, J.L. (2010). The PAF complex synergizes with MLL fusion proteins at HOX loci to promote leukemogenesis. Cancer Cell *17*, 609–621.

Okuda, H., Kawaguchi, M., Kanai, A., Matsui, H., Kawamura, T., Inaba, T., Kitabayashi, I., and Yokoyama, A. (2014). MLL fusion proteins link transcriptional coactivators to previously active CpG-rich promoters. Nucleic Acids Res. *42*, 4241–4256.

Pui, C.H., Carroll, W.L., Meshinchi, S., and Arceci, R.J. (2011). Biology, risk stratification, and therapy of pediatric acute leukemias: an update. J. Clin. Oncol. *29*, 551–565.

Risner, L.E., Kuntimaddi, A., Lokken, A.A., Achille, N.J., Birch, N.W., Schoenfelt, K., Bushweller, J.H., and Zeleznik-Le, N.J. (2013). Functional specificity of CpG DNA-binding CXXC domains in mixed lineage leukemia. J. Biol. Chem. *288*, 29901–29910.

Whyte, W.A., Orlando, D.A., Hnisz, D., Abraham, B.J., Lin, C.Y., Kagey, M.H., Rahl, P.B., Lee, T.I., and Young, R.A. (2013). Master transcription factors and mediator establish super-enhancers at key cell identity genes. Cell *153*, 307–319.

Wilkinson, A.C., Ballabio, E., Geng, H., North, P., Tapia, M., Kerry, J., Biswas, D., Roeder, R.G., Allis, C.D., Melnick, A., et al. (2013). RUNX1 is a key target in t(4;11) leukemias that contributes to gene activation through an AF4-MLL complex interaction. Cell Rep. *3*, 116–127.

Yokoyama, A., and Cleary, M.L. (2008). Menin critically links MLL proteins with LEDGF on cancer-associated target genes. Cancer Cell *14*, 36–46.

Yokoyama, A., Somervaille, T.C., Smith, K.S., Rozenblatt-Rosen, O., Meyerson, M., and Cleary, M.L. (2005). The menin tumor suppressor protein is an essential oncogenic cofactor for MLL-associated leukemogenesis. Cell *123*, 207–218.

Yokoyama, A., Lin, M., Naresh, A., Kitabayashi, I., and Cleary, M.L. (2010). A higher-order complex containing AF4 and ENL family proteins with P-TEFb facilitates oncogenic and physiologic MLL-dependent transcription. Cancer Cell *17*, 198–212.

Zhu, L., Li, Q., Wong, S.H., Huang, M., Klein, B.J., Shen, J., Ikenouye, L., Onishi, M., Schneidawind, D., Buechele, C., et al. (2016). ASH1L links histone H3 lysine 36 dimethylation to MLL leukemia. Cancer Discov. *6*, 770–783.

Cell Reports, Volume *18*

Supplemental Information

MLL-AF4 Spreading Identifies Binding Sites that Are Distinct from Super-Enhancers and that Govern Sensitivity to DOT1L Inhibition in Leukemia

Jon Kerry, Laura Godfrey, Emmanouela Repapi, Marta Tapia, Neil P. Blackledge, Helen Ma, Erica Ballabio, Sorcha O'Byrne, Frida Ponthan, Olaf Heidenreich, Anindita Roy, Irene Roberts, Marina Konopleva, Robert J. Klose, Huimin Geng, and Thomas A. Milne

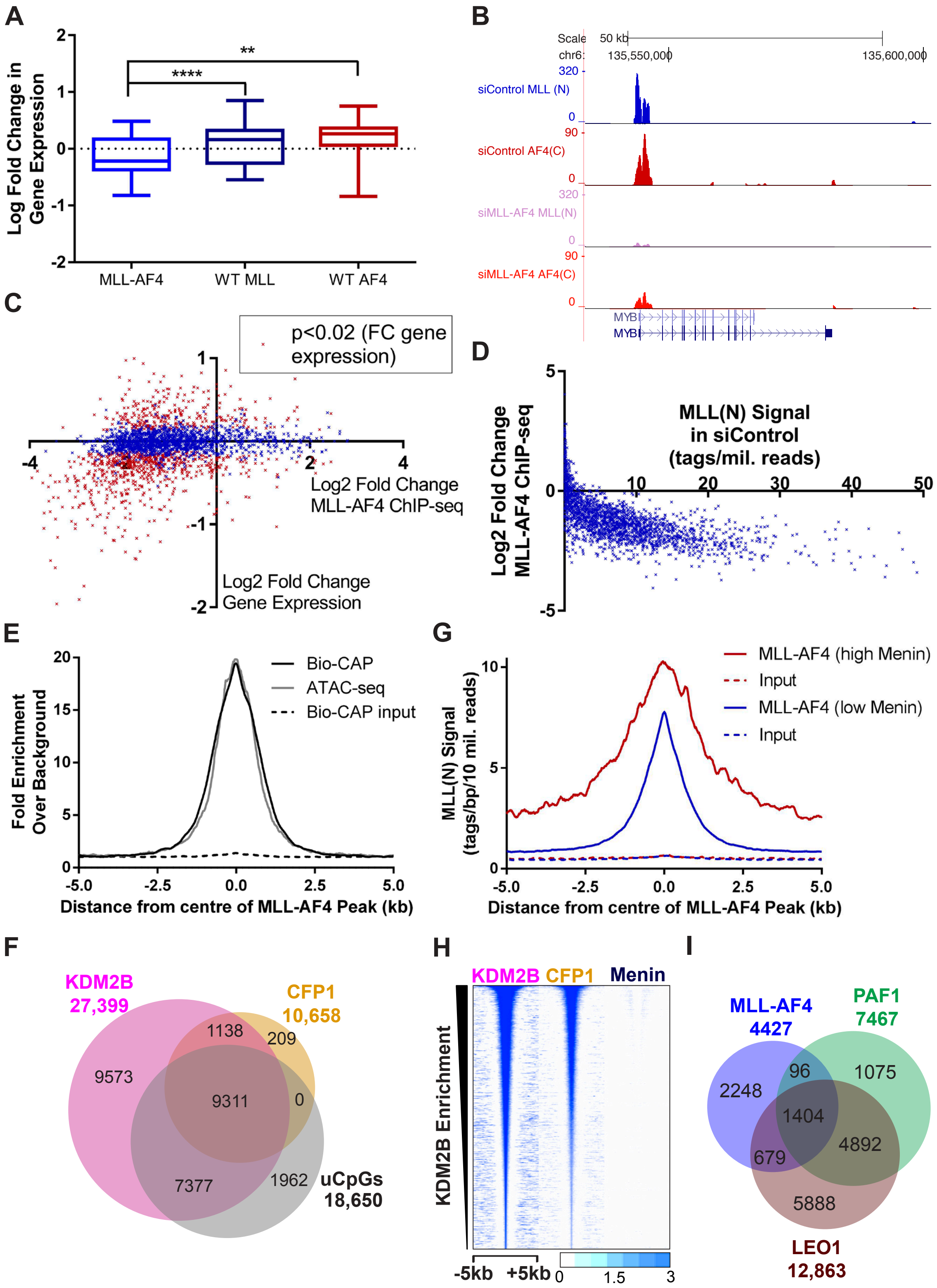

Figure S1, MLL-AF4 recruitment, Related to Figure 1. (**A**) Box and whisker plot showing the median and IQ range of fold change in gene expression, measured by nascent RNA-seq, of all significantly affected gene targets of MLL-AF4 (MLL(N) ChIP-seq peaks that overlap with AF4(C) peaks), wild-type MLL1 (MLL(N) peaks that do not overlap with AF4(C)) and wild-type AF4 (AF4(C) peaks that do not overlap with MLL(N)), following treatment with an MLL-AF4-specific siRNA in SEM cells. (**B**) Example ChIP-seq tracks showing MLL(N) and AF4(C) in SEM cells treated with either control siRNA (top two lanes), or MLL-AF4-specific siRNA (bottom two lanes). (**C**) Scatter plot showing the log2 fold-change in MLL-AF4 ChIP-seq (mean average fold-change of MLL(N) + AF4(C)) and log2 fold change in gene expression following treatment of SEM cells with MLL-AF4-specific siRNA, at all MLL-AF4 gene targets. Red points show genes that had a significant ($p<0.02$) fold-change in gene expression. (**D**) Scatter plots showing how log2 fold-change in MLL-AF4 ChIP (same data as in (C)) is related to the amount of MLL(N) signal at MLL-AF4 gene targets in SEM cells treated with control siRNA. (**E**) Composite binding plot of uCpG (Bio-CAP and ATAC) reads at MLL-AF4 binding sites in SEM cells. (**F**) Venn diagram showing the overlap between KDM2B and CFP1 binding sites with uCpG regions (Bio-CAP/ATAC-seq), in SEM cells. (**G**) Composite binding plot showing MLL(N) ChIP-seq reads at MLL-AF4 binding sites that overlap with high levels of Menin ChIP-seq reads (red) or low levels of Menin ChIP-seq reads (blue), in SEM cells. (**H**) Heat-map showing ChIP-seq reads of KDM2B, CFP1 and Menin at all 27,399 KDM2B binding sites in SEM cells. Scale bar represents tags per bp per 107 reads. (**I**) Venn diagram showing the overlap between MLL-AF4 and PAFc (PAF1 and LEO1) binding sites .

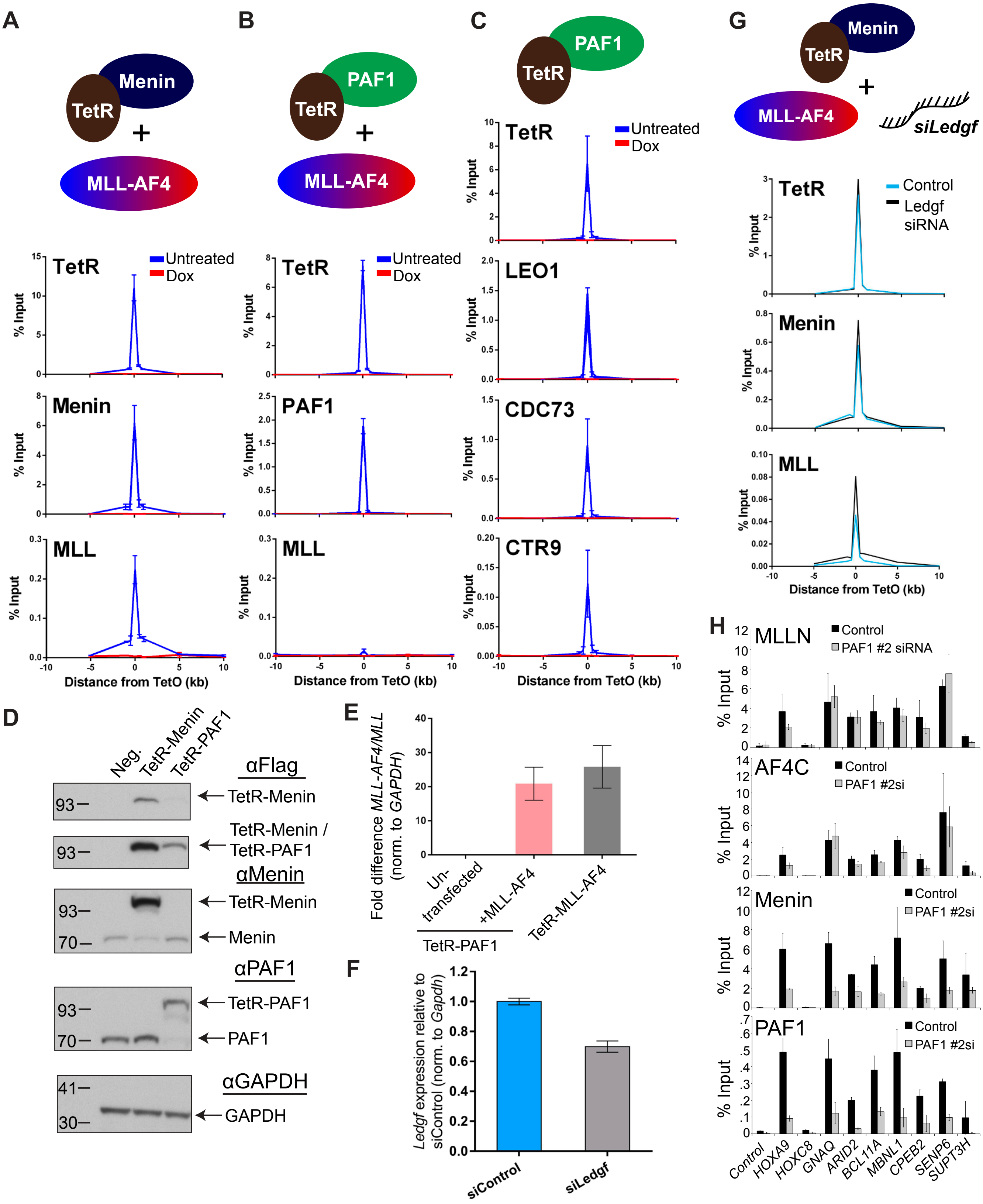

Figure S2, Menin but not PAF1 recruits MLL-AF4, Related to Figure 2. (**A**) ChIP-qPCR showing the binding of Menin and MLL-AF4 (MLL(N)) at the TetO array in TetR-Menin mES cells transfected with MLL-AF4, in the presence (red) or absence (blue) of doxycycline. (**B**) ChIP-qPCR showing the binding of PAF1 and MLL-AF4 in TetR-PAF1 mES cells transfected with MLL-AF4. (**C**) ChIP-qPCR showing the binding of the PAFc members LEO1, CDC73 and CTR9 at the TetO array in TetR-PAF1 mES cells. (**D**) Western blot showing the expression of TetR-Menin and TetR-PAF1 in TOT2N mES cells, stably expressing each protein. (**E**) cDNA levels of the human MLL-AF4 fusion gene after transfection of TOT2N mES cells with either MLL-AF4 or TetR-MLL-AF4, compared to untransfected cells. Error bars represent the standard deviation of 2 biological replicates. (**F**) cDNA levels of mouse Ledgf after transfection of TetR-Menin mES cells with either control siRNA or Ledgf siRNA, normalized to control. Error bars represent the standard deviation of 2 technical replicates. (**G**) ChIP-qPCR showing the binding of TetR-Menin and MLL-AF4 at the TetO array in TetR-Menin mES cells transfected with MLL-AF4 and Ledgf-specific siRNA (related to (F)). (H) MLL(N), AF4(C), Menin and PAF1 ChIP in SEM cells treated with either control (black bars) or PAF1#2 siRNAs (gray bars). Bars represent the average of two independent experiments and error bars = S.D. between experiments.

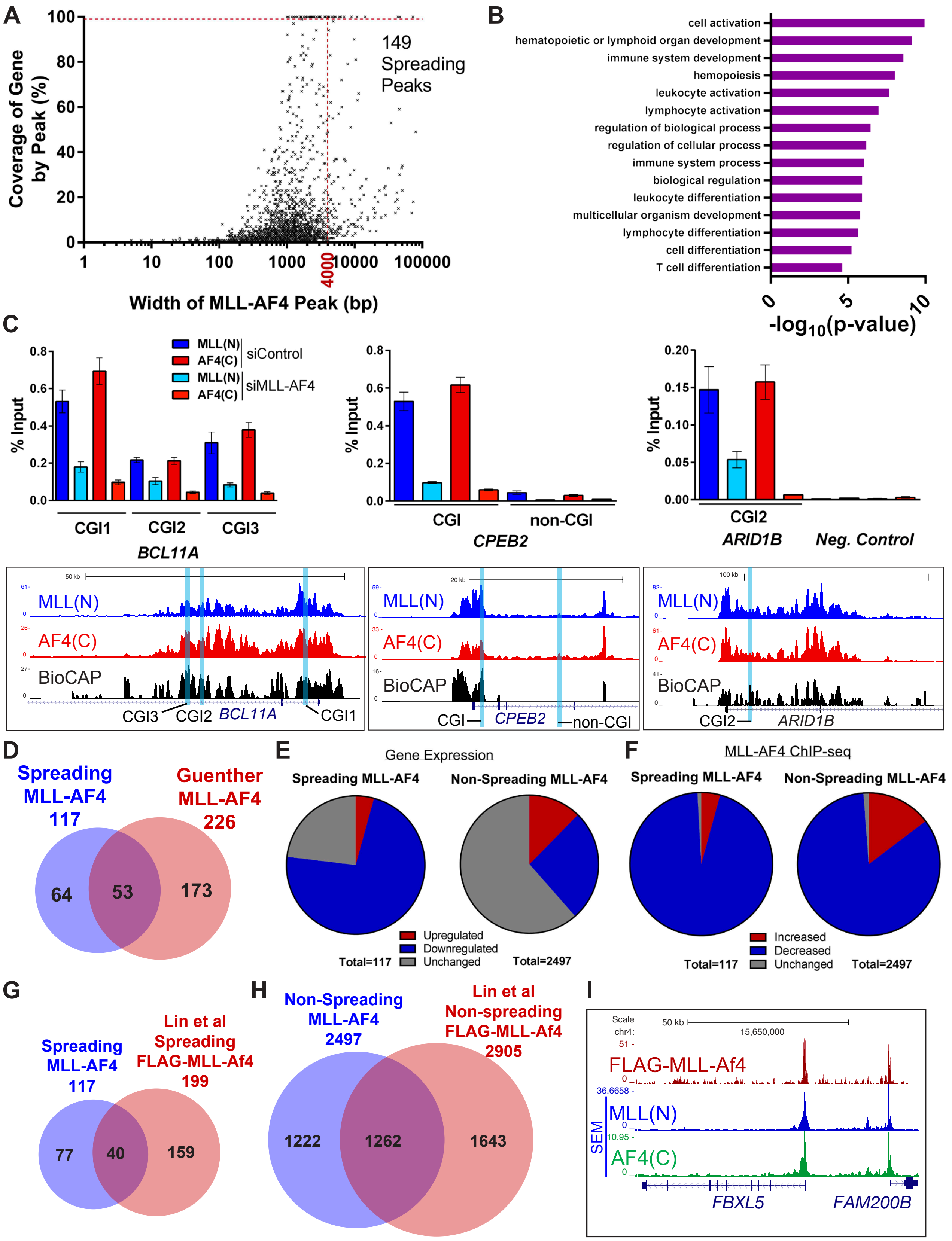

Figure S3, MLL-AF4 binding shows spreading, Related to Figure 3. (**A**) Spreading MLL-AF4 peaks were defined as peaks that extend greater than 4kb from the TSS into the gene body without going beyond the end of the gene. Using these criteria, 149 spreading MLL-AF4 peaks were identified in SEM cells. (**B**) Gene ontology analysis for all gene targets of spreading MLL-AF4 in SEM cells. (**C**) Spreading MLL-AF4 at specific gene targets confirmed using MLL-AF4 siRNA treatment followed by ChIP-qPCR of MLL(N) and AF4(C) (top). ChIP-seq tracks (bottom) show locations of the primers used. (**D**) Venn diagram showing the overlap between spreading MLL-AF4 gene targets in SEM cells and all MLL-AF4 targets identified in SEM cells by Guenther et al. (Guenther et al., 2008). (**E**) Pie charts showing the proportion of spreading MLL-AF4 and non-spreading MLL-AF4 gene targets in SEM cells that are either significantly ($p<0.02$) upregulated (red), downregulated (blue) or unchanged (grey), following treatment with MLL-AF4-specific siRNAs. Data represents nascent RNA-seq from 3 biological replicates. (**F**) Pie charts showing the proportion of MLL-AF4 peaks at spreading and non-spreading gene target promoters in SEM cells that show an increase (red), decrease (blue) or no change (grey) in MLL-AF4 ChIP-seq signal following MLL-AF4c siRNA treatment. (**G-H**) Venn diagrams showing the overlap between spreading and non-spreading MLL-AF4 gene targets in SEM cells compared to a FLAG-MLL-AF4 ChIP-seq dataset from CD34+ cord blood cells (Lin et al., 2016). (**I**) Example ChIP-seq tracks showing non-spreading at FBXL5 and FAM200B in both MLL-AF4 SEM cells and FLAG-MLL-Af4 cord blood cells (Lin et al., 2016).

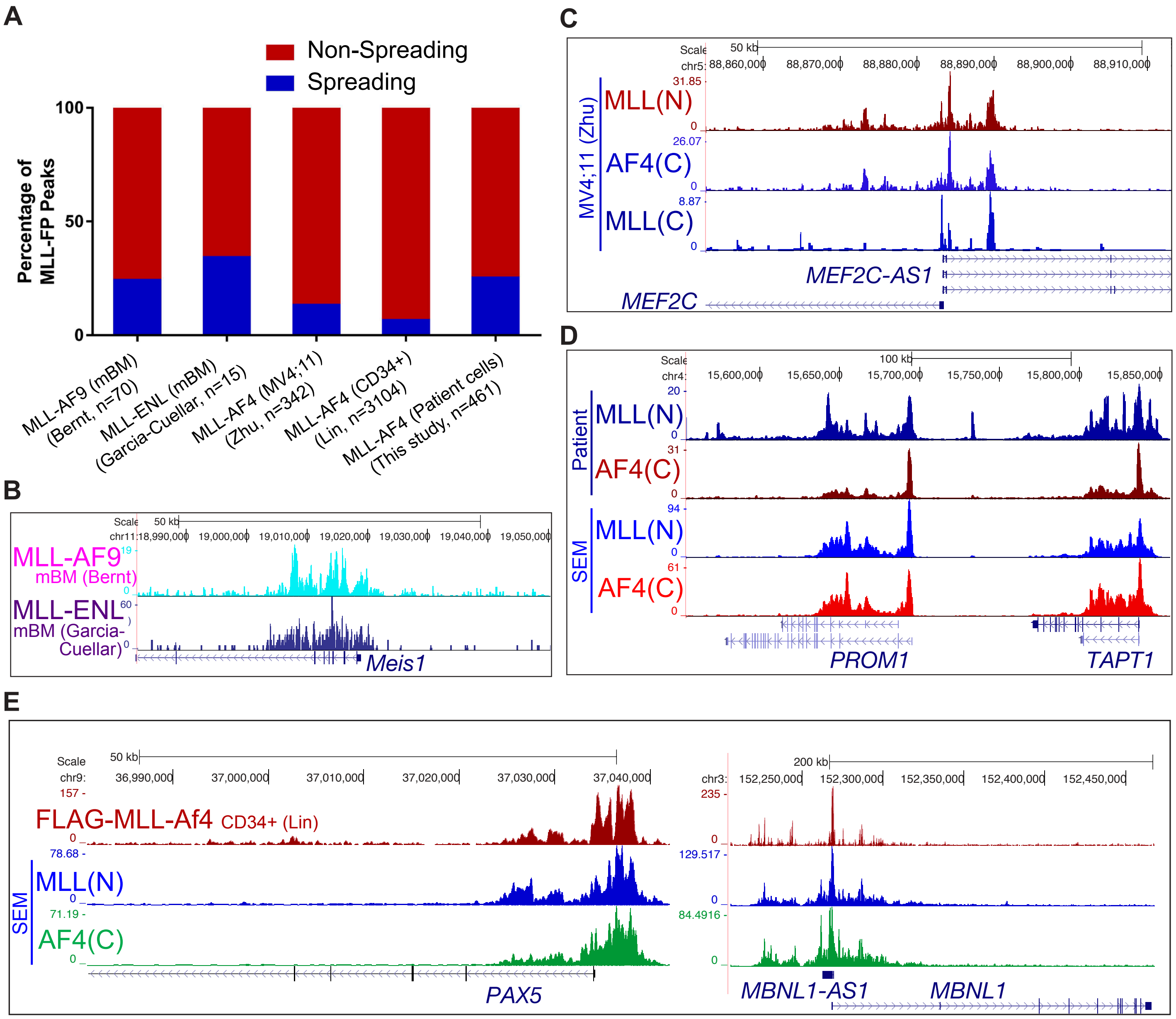

Figure S4, MLL-FPs display spreading but wild type MLL does not, Related to Figure 4. (**A**) Bar plot showing the percentages of spreading and non-spreading peaks within MLL-FP protein peaks in previously published datasets: MLL-AF9 (Bernt et al., 2011), MLL-ENL (Garcia-Cuellar et al., 2016) and MLL-AF4 (Zhu et al., 2016), as well as amongst MLL-AF4 peaks in patient cells. (**B**) Example ChIP-seq tracks showing spreading MLL-AF9 and spreading MLL-ENL at Meis1 in mouse bone marrow cells (datasets same as in (A)). (**C**) Example ChIP-seq tracks showing spreading MLL-AF4 (MLL(N) and AF4(C)) at MEF2C in MV4;11 cells (dataset same as in (A)). MLL(C) was used to show the corresponding lack of spreading by wild-type MLL. (**D**) Example ChIP-seq tracks showing spreading MLL-AF4 (MLL(N) and AF4(C)) at PROM1 and TAPT1 in both MLL-AF4 patient cells and SEM cells. (**E**) Example ChIP-seq tracks comparing spreading MLL-AF4 (MLL(N) and AF4(C)) at PAX5 and MBNL1 in both MLL-AF4 SEM cells and FLAG-MLL-Af4 cord blood cells (Lin et al., 2016).

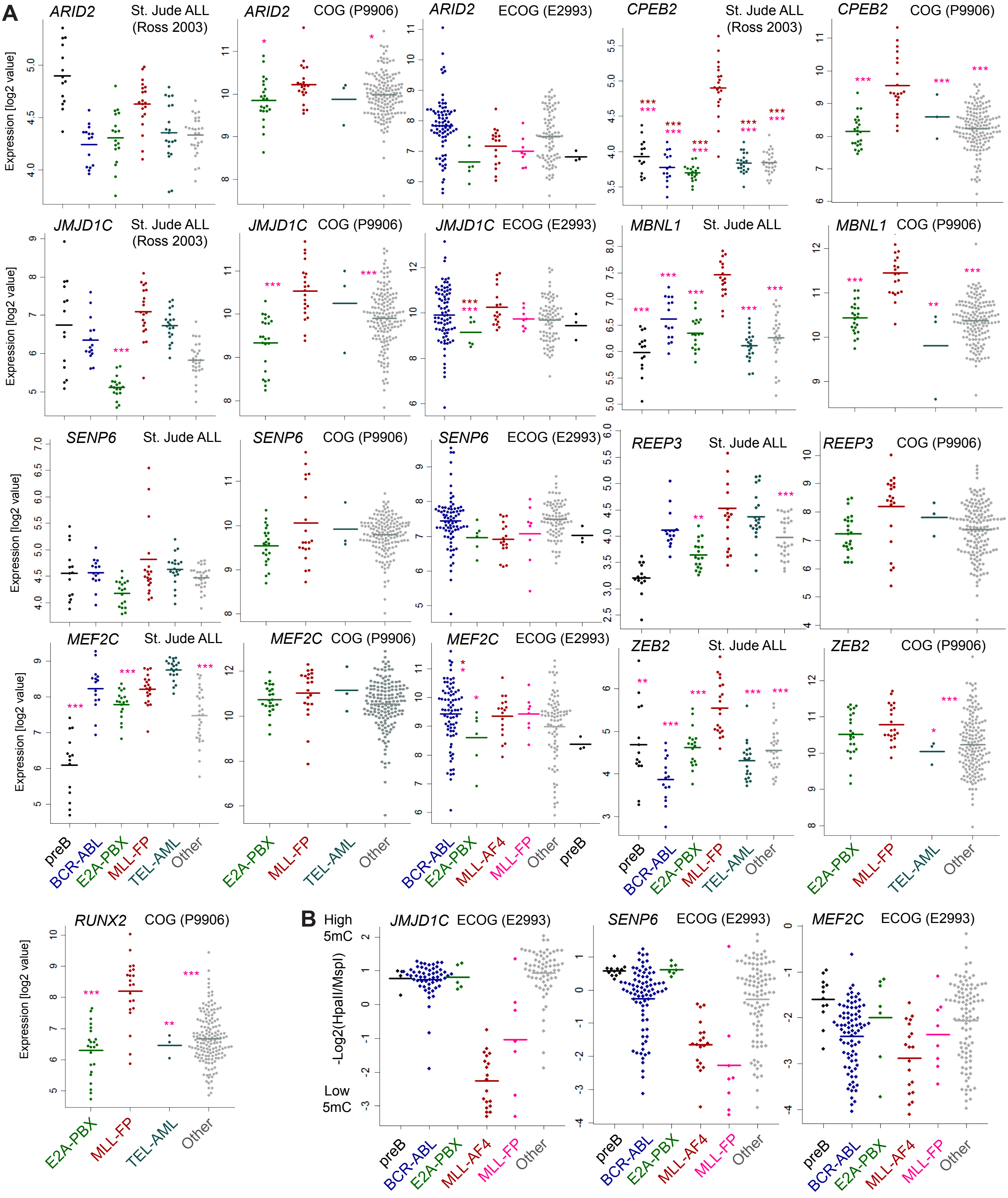

Figure S5, Expression and DNA methylation of spreading targets in patients, Related to Figure 4. (**A**) Expression of the genes indicated in patient samples from three large cohorts of patients with ALL including the Eastern Cooperative Oncology Group (ECOG) Clinical Trial E2993, the Children's Oncology Group (COG) Clinical Trial P9906 and the St. Jude Research Hospital pediatric ALL clinical trial cohort. Details of each trial are listed in supplemental methods. Dark red * indicates a significant difference compared to MLL-AF4, pink * indicates a significant difference compared to the MLL-FP group, no * indicates that the gene is not significantly upregulated in MLL-FP samples. *** = $p<0.001$, ** = $p<0.01$, * = $p<0.05$. All p-values are listed in Table S5. (**B**) DNA methylation data (collected using the HELP assay) for JMJD1C, SENP6 and MEF2C from the (ECOG) Clinical Trial E2993.

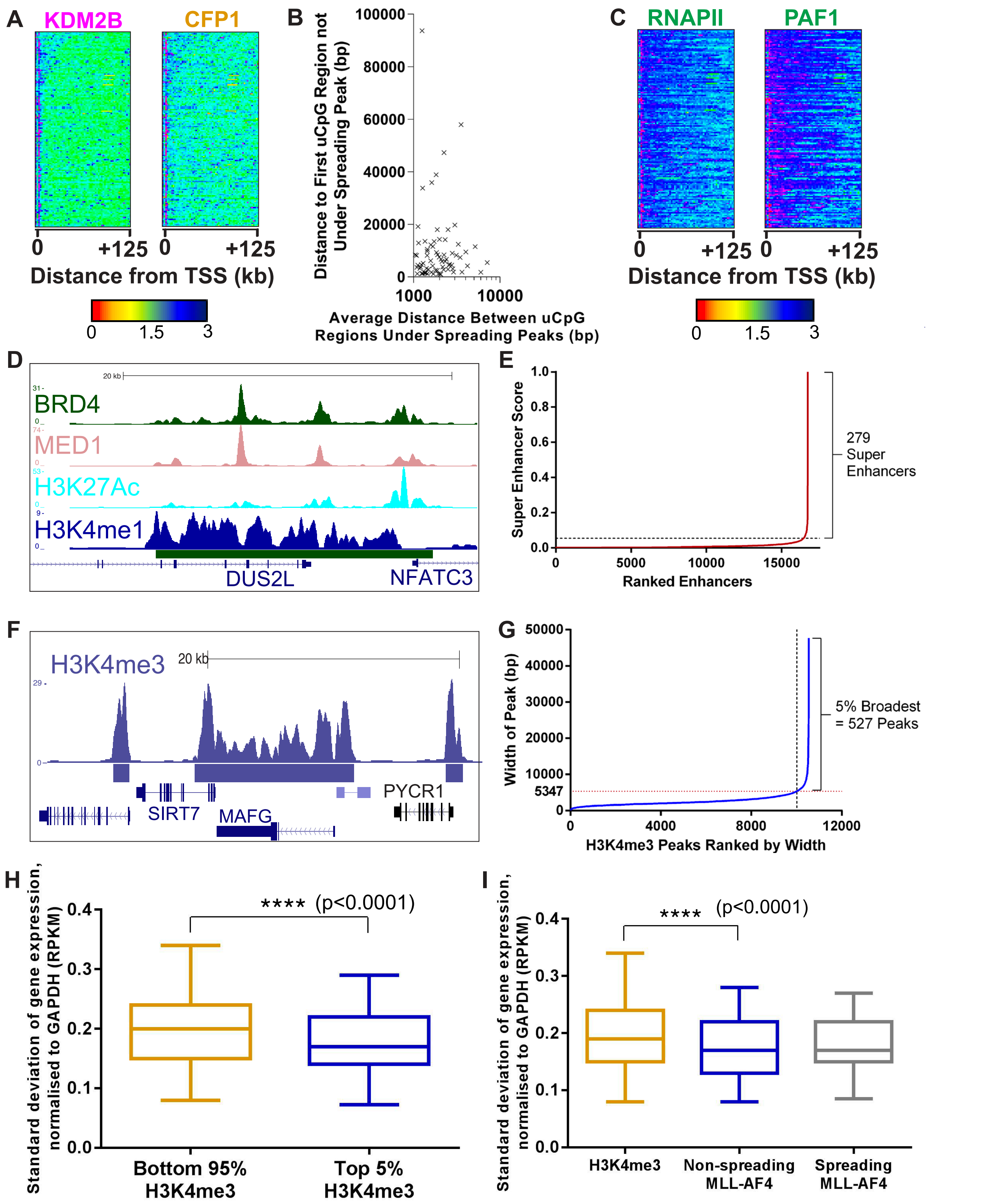

Figure S6, Super enhancers and Broad H3K4Me3 domains, Related to Figure 5. (**A**) Heat-map showing KDM2B (left) and CFP1 (right) ChIP-seq reads at all 149 spreading MLL-AF4 peaks in SEM cells. Scale bar represents tags per bp per 107 reads. (**B**) Scatter plot showing the distance in bp between uCpG regions (Bio-CAP-seq peaks) found under spreading MLL-AF4 peaks against the distance to the first adjacent uCpG region that is not under the spreading peak but found within the body of the same gene. (**C**) Heat-map showing RNA polymerase II (RNAPII) and PAF1 ChIP-seq reads at all 149 spreading MLL-AF4 peaks in SEM cells. Scale bar as in (A). (D) Example ChIP-seq track of a super enhancer at DUS2L in SEM cells. (E) Enhancers characterized by BRD4, MED1, H3K27ac and H3K4me1 in SEM cells were ranked by their super enhancer score. In total, 279 were classed as super enhancers. (F) Example ChIP-seq track of a broad H3K4me3 peak (i.e. one of the top 5% broadest H3K4me3 peaks) spanning SIRT7 and MAFG, in SEM cells. Two normal (non-broad) H3K4me3 peaks are shown either side. (G) All H3K4me3 peaks in SEM were ranked based on length and the top 5% were classified as “broad” H3K4me3. This identified 527 broad H3K4me3 peaks. (H) Box and whisker plot showing the median and IQ range of transcriptional consistency of genes marked by the 5% broadest H3K4me3 peaks (blue) compared to genes marked by the remaining 95% of H3K4me3 peaks (orange). ****=p<0.0001, two-tailed Mann-Whitney U test. (I) Box and whisker plot showing the median and IQ range of transcriptional consistency of gene targets of non-spreading MLL-AF4 (blue,) spreading MLL-AF4 (gray) as well as all genes marked by H3K4me3 (orange). ****=p<0.0001, two-tailed Mann-Whitney U test

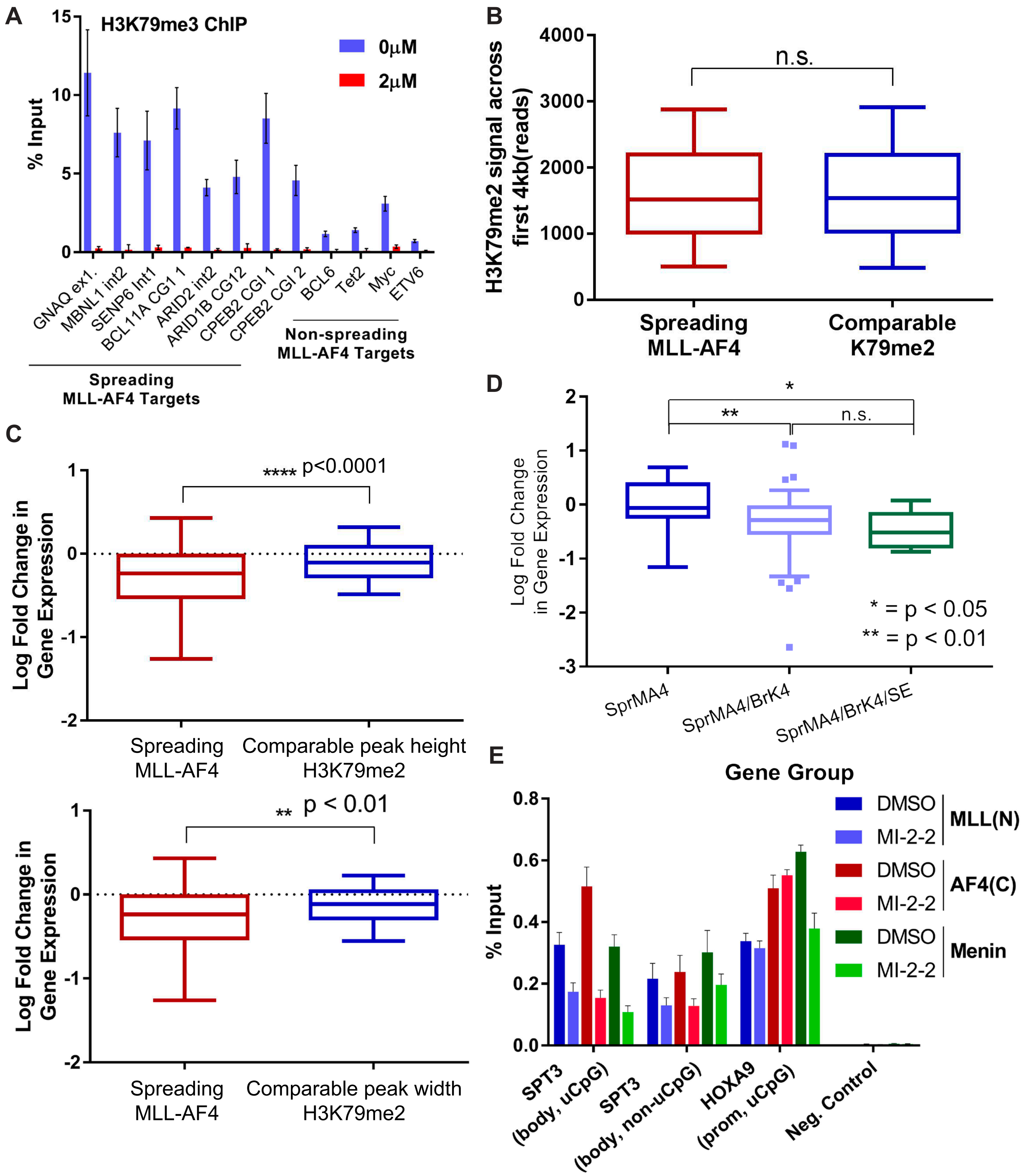

Figure S7, Spreading predicts sensitivity to loss of H3K79me2/3, Related to Figure 6. (**A**) ChIP-qPCR of H3K79me3 at spreading and non-spreading MLL-AF4 gene targets in SEM cells treated with either DMSO control or 2μM EPZ-5676, for 7 days. (**B**) Box and whisker plot showing the median and IQ ranges of H3K79me2 reads across the first 4kb of the gene body of (i) spreading MLL-AF4 targets (n = 117) and (ii) randomly selected genes with comparable levels of H3K79me2 to that of spreading MLL-AF4 targets (n =117). Spreading MLL-AF4 targets were omitted from the selection of genes for group (ii). N.s. = not significant, Mann-Whitney U test. (**C**) Box and whisker plot showing the median and IQ range of fold change in gene expression, measured by nascent RNA-seq, of genes in group (i) and (ii) from (B) following a 7-day treatment of EPZ-5676 (top) and comparing to similar peak width rather than peak height (bottom). ****=p<0.0001,**=p<0.01, Mann-Whitney U test. (**D**) Box and whisker plot showing the median and IQ range of fold change in gene expression, measured by nascent RNA-seq, for gene targets of just spreading MLL-AF4 (SprMA4), both spreading MLL-AF4 and broad H3K4me3 (SprMA4/BrK4), and spreading MLL-AF4, broad H3K4me3 and Super Enhancers (SprMA4/BrK4/SE) following a 7-day treatment with 2μM EPZ-5676. *=p<0.05, **=p<0.01, Mann-Whitney U test. (**E**) ChIP-qPCR of MLL(N), AF4(C) and Menin in SEM cells following treatment with either DMSO or 12.5μM MI-2-2 (Shi et al., 2012) for 48 hours at two loci in the MLL-AF4 spreading domain at SPT3 (one a uCpG region, left, and the other a non-uCpG region, right) and the uCpG promoter of HOXA9. Error bars represent the standard deviation of PCR replicates.

Table S1, (excel file) MLL-FP gene target lists, related to Figure 1 and Figure 4

Table S2, Spreading MLL-AF4 gene targets, Related to Figure 3 and Figure S3A.

Gene Name (117)	Gene Isoforms (149), RefSeq ID
ADAM10	NM_001110
AFF1	NM_001166693
ANP32A	NM_006305
APOLD1	NM_001130415
ARHGDIB	NM_001175
ARID1B	NM_020732
ARID2	NM_152641
ARPP21	NM_001267617; NM_016300; NM_001267619
ARRDC3	NM_020801
ATP8B4	NM_024837; NR_073598
BCL11A	NM_022893
BCL2	NM_000633
C5orf56	NR_045116
CACNB4	NM_001005746; NM_000726
CAMK2D	NM_172127
CCDC162P	NR_028595
CDCA7	NM_031942
CDK14	NM_012395
CDK6	NM_001145306; NM_001259
CELF2	NM_001083591
CHD2	NM_001271
CLEC2B	NM_005127
CLEC2D	NM_001197319
CLECL1	NM_001253750
CNST	NM_001139459
CPEB2	NM_001177383
CTBP2	NM_001083914; NM_001329
CXorf21	NM_025159
CXXC5	NM_016463
CYTIP	NM_004288
DGKD	NM_152879
DIAPH1	NM_005219
EBF1	NM_024007
ELOVL6	NM_024090; NM_001130721
ERG	NM_001136155
EVI2B	NM_006495
FLT3	NM_004119
FUT4	NM_002033
GNAQ	NM_002072
HIPK3	NM_001278163; NM_005734; NM_001278162
HIVEP2	NM_006734
HMGA2	NM_003483

HMGB1	NM_002128
HNRNPF	NM_001098205; NM_004966; NM_001098204
IER2	NM_004907
IGF1R	NM_000875
IKZF1	NM_001220767
JMJD1C	NM_032776; NM_004241
KLRC4-KLRK1	NM_001199805
KLRK1	NM_007360
LAT2	NM_032464
LEF1	NM_016269; NM_001166119
LEF1-AS1	NR_029374
LMO4	NM_006769
LOC728175	NR_040108
LRMP	NM_001204126
LRRFIP2	NM_001134369
MAFG	NM_032711
MAP3K1	NM_005921
MBNL1	NM_207297; NM_207292
MEF2C	NM_001193349; NM_002397
MEIS1	NM_002398
NR3C1	NM_001018076; NM_001204259
NUSAP1	NM_018454
PAN3	NM_175854
PAX5	NM_016734
PDE4DIP	NM_001002811
PHLPP1	NM_194449
PIK3CD	NM_005026
PKM	NM_001206798
PLEK	NM_002664
PPP2R5C	NM_001161726; NM_178586
PPP6R1	NM_014931
PROM1	NM_001145848; NM_001145847
PTEN	NM_000314
PTPN6	NM_002831
PTPRR	NM_001207015; NR_073474; NM_001207016
RCC1	NM_001048199
REEP3	NM_001001330
RHOH	NM_001278368; NM_004310; NM_001278367; NM_001278369
RNF19B	NM_153341
RNF220	NM_018150
ROBO1	NM_133631
RPSAP52	NR_026825
RUNX1	NM_001754; NM_001122607
RUNX2	NM_001015051; NM_001278478; NM_004348
SEMA3A	NM_006080

SENP6	NM_001100409
SMC4	NM_005496; NM_001002800
SOX11	NM_003108
SPEN	NM_015001
SPN	NM_001030288
SPRY4	NM_030964
ST8SIA4	NM_175052; NM_005668
STC2	NM_003714
STK17B	NM_004226
SUPT3H	NM_003599
SYK	NM_001135052; NM_001174168
SYT1	NM_001135805
TAPT1	NM_153365
TAPT1-AS1	NR_027697
TBC1D14	NM_001113361; NM_020773
TGFBR2	NM_001024847
TGIF1	NM_173209; NM_173208; NM_173207
TMPO	NM_001032284
TNRC18	NM_001080495
TPD52	NM_001025253
TRHDE	NM_013381
TRHDE-AS1	NR_026837
TSC22D2	NM_014779
UBASH3B	NM_032873
ZC3H12C	NM_033390
ZC3HAV1	NM_020119
ZCCHC6	NM_001185059
ZCCHC7	NM_032226
ZEB2	NM_014795
ZMYND8	NM_012408

Table S3, Spreading MLL-AF6 gene targets, Related to Figure 4A.

Gene Name (38)	Gene Isoforms (47), RefSeq ID
ADAMTS19	NM_133638
APOLD1	NM_001130415
ARID2	NM_152641
CD69	NM_001781
CDK13	NM_031267
CLEC2A	NM_001130711
CLEC2B	NM_005127
CPEB2	NM_001177382
DACH1	NM_004392
DLX6-AS1	NR_015448
EMB	NM_198449
FAM169A	NR_046462; NM_015566
FOXP1	NM_001012505; NM_001244808
FRY	NM_023037
JMJD1C	NM_032776
LOC646762	NR_024278
MBNL1	NM_021038
MEF2C	NM_001193349; NM_001193350
MYB	NM_001161659
MYO6	NM_004999
NPAS3	NM_001164749
PARP8	NM_001178055; NM_024615
PTPRK	NM_002844; NM_001135648
RBMS1	NM_002897
REEP3	NM_001001330
RNF220	NM_018150
RUNX2	NM_004348; NM_001278478
SATB1	NM_001131010; NM_002971
SENP6	NM_015571
SSPN	NM_005086; NM_001135823
SUPT3H	NM_181356
SYDE2	NM_032184
TAPT1-AS1	NR_027697
TCF4	NM_001243226; NM_001243230
TCTEX1D1	NM_152665
TRPS1	NM_014112
ZEB2	NM_014795
ZNF521	NM_015461

Table S4, Gene targets common to 5 spreading MLL-FPs, Related to Figure 4G.

Gene Name (9)	Gene Isoforms (10), RefSeq ID	MRD (COG)	OS (COG or ECOG)	Relapse
ARID2	NM_152641		poor	
CPEB2	NM_001177382			
JMJD1C	NM_032776			✔
MBNL1	NM_021038	✔		✔
MEF2C	NM_001193349			
REEP3	NM_001001330			
RUNX2	NM_004348; NM_001278478	✔		✔
SENP6	NM_015571			
ZEB2	NM_014795			

Table S5, (excel file) P-values for patient gene expression data, Related to Figure 4 and Figure S5

Table S6, Spreading MLL-AF4 gene targets downregulated at different concentrations of EPZ-5676, Related to Figure 7A.

1µM unique	2µM unique	1µM & 2µM overlap	05µM, 1µM & 2µM overlap
TPD52	STC2	LEF1	CPEB2
SENP6	CNST	RUNX1	PROM1
LRRFIP2	PHLPP1	SPRY4	AFF1
	RNF220	JMJD1C	ERG
	TNRC18	LEF1-AS1	ARHGDIB
	ANP32A	BCL2	HMGA2
	SOX11	CDCA7	DIAPH1
	PPP6R1	ZMYND8	BCL11A
	PKM	CACNB4	ZC3HAV1
	APOLD1	ZC3H12C	UBASH3B
	PAX5	ELOVL6	ARPP21
	IER2	HIPK3	CTBP2
		TSC22D2	HIVEP2
		SUPT3H	TGFBR2
		TRHDE	ARID1B
		SYT1	CDK6
		PPP2R5C	IKZF1
		SPEN	IGF1R
		HNRNPF	
		MEF2C	
		ARID2	
		TBC1D14	
		CAMK2D	
		CELF2	
		TAPT1	
		TRHDE-AS1	
		MAFG	
		ZCCHC7	
		CDK14	

Table S7, Datasets from previous publications, Related to Figures 1 and 3-6

ChIP-seq		
**Cell Line**	**Antibody**	**Accession Number**
SEM	MLLN	GEO: GSE74812
	AF4	
	H3K4me3	
	H3K4me1	
	H3K27ac	
	H3K79me2	
	CFP1	
	ENL	
	ATAC-seq	
	(MLL-AF4 gene list)	GEO: GSE13313
MV4;11	MLLN	GEO: GSE73528
	MLLC	
	AF4C	
Human CD34+	FLAG (MLL-Af4)	GEO: GSE84116
mES	Streptavidin (MLL-AF9)	GEO: GSE29130
	ER (MLL-ENL)	ArrayExpress: E-MTAB-3593

Patient data		
**Clinical Trial**	**Patient Type**	**Accession Number**
ECOG E2993	Various ALL subtypes	GEO: GSE34861
COG P9906	Various ALL subtypes	GEO: GSE28460
St Jude's Research Hospital	Various pediatric ALL subtypes	http://www.stjuderesearch.org/site/data/ALL3/

Supplemental Experimental Procedures

Cell lines and culture
SEM, (Greil et al., 1994) ML-2 (Ohyashiki et al., 1986), RCH-ACV (Jack et al., 1986) and KOPN-8 (Matsuo and Drexler, 1998) were purchased from DSMZ (www.cell-lines.de) and cultured in IMDM or RPMI 1640 supplemented with 10% FCS. THP1, MV4-11 and CCRF-CEM cells were purchased from ATCC (www.lgcstandards-atcc.org) and cultured in RPMI 1640 supplemented with 10% FCS. SEM-K2 were kindly provided by Dr. Carolyn Felix (University of Pennsylvania, Philadelphia, PA). SEM-K2 cells are a subclone of SEM cells with identical features (Zweidler-McKay et al., 2005). HEK293T cells were cultured in DMEM supplemented with 10% FCS. Mouse ES cells with a TetO-array (TOT2N mESC) were kindly provided by Dr. Rob Klose (University of Oxford) and were grown in DMEM supplemented by with 10% FCS, LIF and β-mercaptoethanol. TOT2N mESCs were treated with doxycycline for 6 hours when appropriate for the ChIP studies. For the nascent RNA-seq studies, SEM cells were treated with the DOT1L inhibitor EPZ-5676 (Epizyme) for 7 days, with fresh inhibitor added on days 0, 3 and 6.

Drug Treatment Studies
SEMK2 cells were seeded at 40,000 cells /well in a 96 well plate. The cells were treated with a DMSO control, ABT-199 (320, 160, 80, 40, 20, 10, 5 nM and DMSO control) alone, or in combination with a 1:10 ratio of either EPZ5676, SGC0946 or MI503 (3200, 1600, 800, 400, 200, 100, 50 nM and DMSO control). Media was changed once at 72 hr, cells were split and kept at about 40,000 cells/well, CellTiter-Glo® Luminescent Cell Viability Assay was run at 7days. Calcusyn 2.0 software (Biosoft, Great Shelford, UK) was used to calculate IC_{50} values and combination index.

Human progenitor cell isolation
Samples: Mononuclear cells (MNC) from term cord blood (CB) and second trimester human fetal bone marrow (FBM) were used for the experiments. CB was collected under the auspices of a National Research Ethics Service-approved study with written informed consent. Human fetal bone samples were obtained through the Human Developmental Biology Resource (www.hdbr.org).
Sample preparation: FBM was obtained by repeated flushing of the fetal long bones with DMEM (Gibco). CB and FBM samples were red cell and granulocyte depleted by density gradient separation with Ficoll-Paque Premium (GE Healthcare) to isolate MNC. MNC were either fixed fresh or following thawing after cryopreservation.

Patient-derived MLL-AF4 primograft cells
L826 patient cells were obtained from Newcastle Haematological BioBank and as such are covered by a generic approval given by the Newcastle & North Tyneside Ethics Committee (REC reference number: 07/H0906/109+5). The mouse transplantation experiments are covered by a Home Office Project licence PPL 60/4552. The L826 cells were originally derived from a diagnostic ALL patient sample, which have been passed through an mouse prior to ChIP-seq analysis. To that end, L826 patient cells were engrafted in NSG mice and the primograft was harvested from a mouse spleen. Cells were thawed and maintained in RPMI with 20% serum before they were fixed for ChIP-seq experiments.

siRNA experiments
Briefly, using a rectangle pulse EPI 2500 electroporator (Fischer, Heidelberg), 7 X 10^7 SEM cells were subjected to a 10msec 350V (SEM) electroporation in the presence of 300 pmol siRNA. MLLAF4 siRNA sequences were obtained from (Thomas et al., 2005) and are the following: siMA6 (sense, AAGAAAAGCAGACCUACUCCA; antisense, UGGAGUAGGUCUGCUUUUCUUUU), targeting the MLL exon 9 and AF4 exon 4 MLL-AF4 fusion site present in SEM cells. As control siRNAs we used the mismatch control siMM (sense,AAAAGCUGACCUUCUCCAAUG; antisense, CAUUGGAGAAGGUCAGCUUUUCU). For Menin and PAF1 siRNA experiments in SEM cells, we used Ambion Silencer Select Negative Control #2 (catalog AM4613), PAF1#1 (4392420, s29267), PAF1#2 (4392420, s29269) and Menin (4392420, s8682). Electroporation conditions were the same as for MLL-AF4 siRNAs. For testing MLL-AF4 recruitment by Menin and PAF1, MLL-AF4 cDNA was transfected into TetR-Menin and TetR-PAF1 mES cells at 60-70% confluency, either alone or with mouse Ledgf (Psip1) siRNA (Dharmacon SMARTpool, L-056571-01-0005), using Lipofectamine 2000. Cells were collected 24 hours after transfection.

Antibodies used for western blot analysis
αMenin (Bethyl, A300-105A), αGAPDH (Bethyl, A300-641A), αFlag (Sigma, F1804), αPAF1 (Bethyl, A300-172A), αCDK6 (Cell Signaling, 3136), αBCL11A (Bethyl, A300-382A), αBCL2 (Cell Signaling, 2870),

αRUNX1 (Cell Signaling, 4334), αMEF2C (Cell Signaling, 5030), αH3K79me3 (Diagenode, C15410068), αH4 (Abcam, ab7311)

Chromatin immunoprecipitation assays

For ChIP and ChIP-seq, fixed samples of up to 10^8 cells were sonicated on a Covaris (Woburn, MA) according to the manufacturers' recommendations. Ab:chromatin complexes were collected with a mixture of Protein A and Protein G Dynabeads (Life Technologies, Grand Island, NY) by using a magnet and were then washed three times with a solution of 50mM Hepes-KOH, pH 7.6, 500mM LiCl, 1mM EDTA, 1% NP-40, and 0.7% Na-deoxycholate. After a Tris-EDTA wash, samples were eluted, treated with RNAse and proteinase K, and purified by using a Qiagen PCR purification kit. ChIP samples were quantified relative to inputs (Milne et al., 2009). Briefly, the amount of genomic DNA co-precipitated with antibody was calculated as a percentage of total input using the following formula: $\Delta CT = CT$ (input) – CT (ChIP), total percentage = $2^{\Delta CT} \times 5.0\%$. A 50-μL aliquot taken from each of 1 mL of sonicated, diluted chromatin before Ab incubation served as the input, and thus the signal from the input samples represents 5% of the total chromatin used in each ChIP. CT values were determined by choosing threshold values in the linear range of each PCR reaction. TetR fusion proteins were detected in ChIP using an FS2 antibody.

ChIP sequencing

ChIP samples were submitted to the Wellcome Trust Centre for Human Genetics for library preparation (Lamble et al., 2013) and sequencing. Samples were sequenced using a HiSeq 2000, a HiSeq 2500 and 50bp paired-end sequencing. Data were mapped to the *Homo sapiens* hg18 or *Mus musculus* mm10 genome using Bowtie. Conversion to bam files was done using samtools. Duplicate reads were removed and data was normalized to an input track, in SeqMonk. Peaks were called using the probe generator in SeqMonk and filtered to retain only those that showed a >3-fold enrichment over the input track. MLL-AF4 peaks were defined as any MLL(N) peaks that overlapped with at least one AF4(C) peak. Wild-type MLL peaks in SEM cells were defined as any MLL(N) peaks that did not overlap with an AF4(C) peak. Broad H3K4me3 peaks were defined as the top 5% widest peaks (kb) of H3K4me3, as in the original study (Benayoun et al., 2014). Super enhancers were defined using the HOMER (http://homer.salk.edu/homer/index.html) *findPeaks –style super* command, based on the algorithm of the original study (Whyte et al., 2013), with a combined BRD4, MED1, H3K27ac and H3K4me1 dataset. Data for heat maps and meta gene plots were generated using the HOMER *annotatePeaks* command. Heat maps of spreading peaks in Figure 5 were generated by using the Samtools *view –c* command to get the reads under the peaks in 2.5kb bins. The R package *ggplot2* was used to generate the heat map.

Antibodies used for ChIP and ChIP-seq assays

αH3K36me3 (Diagenode, pAb-192-050), αH3K27ac (Diagenode, C15410196), αH3K4me3 (Diagenode, pAb-003-050), αH3K4me1 (Diagnode, pAb-194-050), αH3K79me2 (Active Motif, 39143), αH3K79me3 (Diagenode, pAb-068-050), αMLL1 (Bethyl, A300-086A), αAF4 (abcam, 31812), αKDM2B (gift from Dr Rob Klose (University of Oxford)), αBRD4 (Bethyl, A301-985A), αMED1 (Bethyl, A300-793A), αMenin (Bethyl, A300-105A), αPAF1 (Bethyl, A300-172A), αLEO1 (A300-175A), αCFP1 (Bethyl, A303-161A), αENL (A302-268A), αFS2 (gift from Dr Rob Klose).

Bio-CAP

Bio-CAP was performed as described previously (Blackledge et al., 2012). Briefly, 50μL biotinylated KDM2B CXXC domain (0.5μg/μL, a generous gift from Dr Rob Klose) was diluted with 425μL BC150 and added to 25μL BC150-rinsed magnetic neutravidin beads (Sera-Mag SpeedBeads NeutraAvidin Microparticles, Thermo Scientific). As a control, 25μL beads were also added to 475μL of BC150 alone. Tubes were rotated end-over-end at 4˚C for 1 hour. Beads were washed 3 times with 1M CAP Buffer (1000mM NaCl; 20mM HEPES pH7.9; 0.1% Triton X-100; 12.5% glycerol), using a magnet, and then 100mM CAP Buffer. 100μL DNA (100ng/μL) was diluted with 900μL BC150 (10μL DNA (10%) was diluted with 90μL BC150 for input). 500μL of DNA in BC150 was added to KDM2B-hybridised beads and the other 500μL was added to the beads-only control. Tubes were incubated end-over-end at 4˚C for 1 hour. After incubation, supernatant was removed from beads using a magnet – this was the flow-through material. Beads were washed twice with 100mM CAP Buffer. Material was then eluted from beads using increasing concentrations of salt: 300mM, 500mM, 700mM and 1M. This was done in 50μL CAP buffer at room temperature for 10 minutes, with gentle tapping, twice to give a total volume of 100μL for each elution. DNA was purified using a Qiagen PCR purification kit.

Nascent RNA-seq

10^8 Cells were treated with 500μM 4-thiouridine (4-SU) for 1 hour. Cells were lysed using trizol and RNA was precipitated with ethanol. 4-SU-incorporated RNA was biotinylated by labelling with 1mg/ml Biotin-HPDP for 90 minutes at room temperature. Following chloroform extraction, labelled RNA was separated using magnetic

streptavidin beads. Beads were washed using a magnetic µMACS stand before RNA was eluted in two rounds of elution with 100µL 100mM DTT. RNA was purified using a Qiagen RNeasy MinElute kit. Samples were sequenced using a NextSeq 500 and 40bp paired-end sequencing.

Nascent RNA-seq and Gene Expression Analysis
Following QC analysis with the fastQC package (http://www.bioinformatics.babraham.ac.uk/projects/fastqc), reads were aligned using STAR (Dobin et al., 2013) against the human genome assembly (NCBI build36 (hg18) UCSC transcripts). Reads that were identified as PCR duplicates using Samtools (Li et al., 2009) were discarded. For experiments that did not involve inhibitor or siRNA treatments, gene expression was measured by calculating the nascent RNA-seq reads over exons for each gene and normalizing to reads per sample and kb of exons and subsequently normalized to GAPDH expression. For inhibitor and siRNA experiments, gene expression levels were quantified as read counts using the featureCounts function (Liao et al., 2014) from the Subread package (Liao et al., 2014) with default parameters. The read counts were used for the identification of global differential gene expression between specified populations using the edgeR package (Robinson et al., 2010). RPKM values were also generated using the edgeR package. Genes were considered differentially expressed between populations if they had an adjusted *p*-value (FDR) of less than 0.05.

Transcriptional Variability
Transcriptional variability of a gene was measured as the standard deviation of expression across four biological replicates of nascent RNA-seq

PCR Primers for RT-PCR
The mouse Mll1 Taqman primer-probe set was as follows: Mll1 primer For: CTGAATGACCTCTCTGACTGTGAAGA, Mll1 probe: ACTCTTTCCTATTGGATACCAGTGTTCTCGGG, Mll1 primer Rev: GGCATCTGTGGTGCTCCAGTA. MLL-AF4 SYBR Green primers were as follows: MLL-AF4 For: AGGTCCAGAGCAGAGCAAAC, MLL-AF4 Rev: CGGCCATGAATGGGTCATTTC. Mouse GAPDH SYBR Green primers were as follows: GAPDH For: GTCTCCTGCGACTTCAGC, GAPDH Rev: TCATTGTCATACCAGGAAATGAGC. Mouse Ledgf For: CGATCAAGAGGGTGAAAAGAA, Ledgf Rev: TTGGCCTTTTAGCATGTTCC

PCR Primers for ChIP
Negative control For: GGCTCCTGTAACCAACCACTACC, Negative control Rev: CCTCTGGGCTGGCTTCATTC; HOXA9 For: ATGCTTGTGGTTCTCCTCCAGTTG, HOXA9 Rev: CCGCCGCTCTCATTCTCAGC; HOXC8 For: AGACTTCTTCCACCACGGCAC; HOXC8 Rev: TAAGCGAGCACGGGTTCTGC; BCL11A CGI1 For: ACACCCAGTGCCCAGAATTG, BCL11A CGI1 Rev: CGCGGGTCCTGAGATTCATT; BCL11A CGI2 For: AGGCTGAGTGTTGTGGAGAC, BCL11A CGI2 Rev: CGTTGGAAGCTGCCTTTGTT; BCL11A CGI3 For: GCCAGGCTAACATAGACCTCC, BCL11A CGI3 Rev: GAGGCCAACTCTTCCACTCC; ARID1B CGI2 For: AAGTGCCGTGACCTTCACAT, ARIB1B CGI2 Rev: CCTTGCATTACCTCTGCCCA; CPEB2 CGI For: ATTGCTGGAGAAGGTGCGTG, CPEB2 CGI Rev: TCCCTCATTGGACAGCGAGA; CPEB2 non-CGI For: CATATGGGCCATCCTATTCTCTG, CPEB2 non-CGI Rev: GCTCTGGTTCTAACTCCTAGAAA; BCL6 For: ACAGAGGCTCAAAGGAAAACAA; BCL6 Rev: GCCTTAACTCCACAAGTTTGA; ARID2 For: GGGACTTGCGATTGGTTATTG; ARID2 Rev: CACCAGAGAGCCCTGTATTT; TET2 For: CCTCAAGGCAGCAACTAAGAAC; TET2 Rev: GAGTCACTCCCAGAGGTCCT; GNAQ For: GTCCATCATGGCGTGCT; GNAQ Rev: CGGACGGTACTCACCGA; MBNL1 For: CAGTCCTTTCACTGCATGTTT; MBNL1 Rev: CAGACAGACACTTTGCCATTAC; SENP6 For: GAGAAGGGAGGGTATACTGGAA; SENP6 Rev: TCGCTCATCCCTCGTACTT; ETV6 For: TTCCTGGTGGCTCCTTTAAGAG; ETV6 Rev: ACTGACGTGAATTCCCAGCA; MYC For: GAGCAGCAGAGAAAGGGAGA; MYC Rev: CAGCCGAGCACTCTAGCTCT; SPT3 uCpG For: CATGTTTTACAGTGCCTGGTACG; SPT3 uCpG Rev: AGACTGCCCTCCTACGCTA; SPT3 non-uCpG For: GCTGAACCCAAATTTATATTGCCC; SPT3 non-uCpG Rev: AGCTCTGAGGGTTACTGACCA

Patient Datasets and gene expression microarray data
Microarray gene expression data from three large cohorts of patients with ALL were analyzed. These cohorts included the Eastern Cooperative Oncology Group (ECOG) Clinical Trial E2993 (GEO#: GSE34861) cohort: 191 total samples comprising 78 BCR-ABL1 patients, 6 E2A-PBX1 patients, 25 MLLr patients (t(4;11): 17, other MLLr: 8), and 82 other B-ALL patients; (Geng et al., 2012) the Children's Oncology Group (COG) Clinical Trial P9906 (GEO#: GSE28460) cohort: 207 total samples, 23 E2A-PBX1 patients, 21 MLLr patients,

3 RUNX1-ETV6 patients, 155 other B-ALL patients (trisomy 4 or 10 patients); (Harvey et al., 2010) and the St. Jude Research Hospital pediatric ALL clinical trial cohort: 132 total samples, 15 BCR-ABL1 patients, 18 E2A-PBX1 patients, 20 MLLr patients, 20 RUNX1-ETV6 patients, 17 hyperdiploid patients, 28 other B-ALL patients, 14 T-ALL patients (Ross et al., 2003). This last cohort has no GEO number, but raw data can be downloaded from the following site: http://www.stjuderesearch.org/site/data/ALL3/. The microarray data was normalized with RMA method (Bolstad et al., 2003) using Expression ConsoleTM software (Version 1.1, Affymetrix, Santa Clara, CA) for the Affymetrix arrays HG-U133 plus2 (COG data, n=207) or NimbleScan software (version 2.5, Roche NimbleGen, Madison, WI) for the NimbleGen arrays HG18 60mer expression 385K platform (ECOG data, n=191). The patients in each clinical trial were grouped into subtypes according to their cytogenetic features: BCR-ABL, TCF3-PBX1, MLLr (MLL rearranged), TEL-AML1, or other ALLs which are negative to the above translocations. The downstream microarray analysis was performed using R version 2.14.0 (R Development Core Team. R: A Language and Environment for Statistical Computing. 2009, http://www.R-project.org). The heatmap was generated with Cluster Cluster/TreeView3.0 software (http://bonsai.hgc.jp/~mdehoon/software/cluster/software.htm).

Survival analysis

In order to test if the "spreading" MLL-AF4 targets (n=117) consist of a prognostic biomarker model predictive of ALL survival we used the **s**upervised **p**rincipal **c**omponents (superPC) algorithm developed by Bair and Tibshirani (Bair and Tibshirani, 2004) in two ALL clinical datasets: COG P9906 (n=207) and ECOG E2993 (n=165). Univariate Cox scores, which measure the correlation between gene expression levels and overall survival (OS) or relapse free survival (RFS) of the ALL patients, were computed for each gene in the gene lists (such as a total of N genes). Only genes with absolute Cox scores of greater than a specific threshold were retained for subsequent prediction (such as M genes, $M<=N$). This threshold was determined using a 10-fold cross-validation procedure that evaluated the prognostic significance of different thresholds within the dataset. A principal component analysis (PCA) was performed using the expression levels of the M genes as features. Then the first three principal components were used in a regression model to compute a prognostic score for each patient. According to these predicted prognostic scores, the patients were divided into two groups: 50% high-risk (> the median score) and 50% low-risk (<= the median score). Not necessarily all the genes get used in the list, this analysis just allows us to conclude that there is a signature within the set that is predictive of a poor prognosis. The procedure was applied in both ALL cohorts. The Kaplan-Meier method was used to estimate overall survival (OS) and relapse-free survival (RFS). Log-rank test was used to compare survival differences between patient groups with high or low risk. The R packages 'Superpc' (Bair and Tibshirani, 2004) and 'survival' version 2.35-8 (Therneau, 2015) was used for the survival analysis.

Supplemental References

Bolstad, B.M., Irizarry, R.A., Astrand, M., and Speed, T.P. (2003). A comparison of normalization methods for high density oligonucleotide array data based on variance and bias. Bioinformatics *19*, 185-193.

Dobin, A., Davis, C.A., Schlesinger, F., Drenkow, J., Zaleski, C., Jha, S., Batut, P., Chaisson, M., and Gingeras, T.R. (2013). STAR: ultrafast universal RNA-seq aligner. Bioinformatics *29*, 15-21.

Greil, J., Gramatzki, M., Burger, R., Marschalek, R., Peltner, M., Trautmann, U., Hansen-Hagge, T.E., Bartram, C.R., Fey, G.H., Stehr, K., *et al.* (1994). The acute lymphoblastic leukaemia cell line SEM with t(4;11) chromosomal rearrangement is biphenotypic and responsive to interleukin-7. Br J Haematol *86*, 275-283.

Jack, I., Seshadri, R., Garson, M., Michael, P., Callen, D., Zola, H., and Morley, A. (1986). RCH-ACV: a lymphoblastic leukemia cell line with chromosome translocation 1;19 and trisomy 8. Cancer Genet Cytogenet 19, 261-269.

Lamble, S., Batty, E., Attar, M., Buck, D., Bowden, R., Lunter, G., Crook, D., El-Fahmawi, B., and Piazza, P. (2013). Improved workflows for high throughput library preparation using the transposome-based Nextera system. BMC Biotechnol *13*, 104.

Li, H., Handsaker, B., Wysoker, A., Fennell, T., Ruan, J., Homer, N., Marth, G., Abecasis, G., Durbin, R., and Genome Project Data Processing, S. (2009). The Sequence Alignment/Map format and SAMtools. Bioinformatics *25*, 2078-2079.

Liao, Y., Smyth, G.K., and Shi, W. (2014). featureCounts: an efficient general purpose program for assigning sequence reads to genomic features. Bioinformatics *30*, 923-930.

Matsuo, Y., and Drexler, H.G. (1998). Establishment and characterization of human B cell precursor-leukemia cell lines. Leuk Res *22*, 567-579.

Milne, T.A., Zhao, K., and Hess, J.L. (2009). Chromatin immunoprecipitation (ChIP) for analysis of histone modifications and chromatin-associated proteins. Methods Mol Biol 538, 409-423.

Ohyashiki, K., Ohyashiki, J.H., and Sandberg, A.A. (1986). Cytogenetic characterization of putative human myeloblastic leukemia cell lines (ML-1, -2, and -3): origin of the cells. Cancer Res *46*, 3642-3647.

Robinson, M.D., McCarthy, D.J., and Smyth, G.K. (2010). edgeR: a Bioconductor package for differential expression analysis of digital gene expression data. Bioinformatics *26*, 139-140.

Ross, M.E., Zhou, X., Song, G., Shurtleff, S.A., Girtman, K., Williams, W.K., Liu, H.C., Mahfouz, R., Raimondi, S.C., Lenny, N., *et al.* (2003). Classification of pediatric acute lymphoblastic leukemia by gene expression profiling. Blood *102*, 2951-2959.

Shi, A., Murai, M.J., He, S., Lund, G., Hartley, T., Purohit, T., Reddy, G., Chruszcz, M., Grembecka, J., and Cierpicki, T. (2012). Structural insights into inhibition of the bivalent menin-MLL interaction by small molecules in leukemia. Blood *120*, 4461-4469.

Therneau, T.M. (2015). A Package for Survival Analysis in S. version 2.35-8. http://cranr-projectorg/package=survival.

Thomas, M., Gessner, A., Vornlocher, H.P., Hadwiger, P., Greil, J., and Heidenreich, O. (2005). Targeting MLL-AF4 with short interfering RNAs inhibits clonogenicity and engraftment of t(4;11)-positive human leukemic cells. Blood *106*, 3559-3566.

Zweidler-McKay, P.A., He, Y., Xu, L., Rodriguez, C.G., Karnell, F.G., Carpenter, A.C., Aster, J.C., Allman, D., and Pear, W.S. (2005). Notch signaling is a potent inducer of growth arrest and apoptosis in a wide range of B-cell malignancies. Blood *106*, 3898-3906.
